# Standard Single Antigen HLA Luminex Panels Predict Most, but Not All, Antibody Reactivity Against Alleles in Extended Panels

**DOI:** 10.1111/tan.70797

**Published:** 2026-07-15

**Authors:** Gerard J. Chu, Rachel L. Cassar, Petrina Guthrie, Anne Taverniti, David Quach, Louise Goddard, Shane Kelly, Federico Gutierrez, Lucy C. Sullivan, Robert P. Carroll

**Affiliations:** ^1^ New South Wales Transplantation and Immunogenetics Services Australian Red Cross Lifeblood Sydney New South Wales Australia; ^2^ Faculty of Medicine and Health The University of Sydney Sydney New South Wales Australia; ^3^ South Australia Transplantation and Immunogenetics Services Australian Red Cross Lifeblood Sydney New South Wales Australia; ^4^ Lung Transplant Service, Department of Respiratory Medicine Alfred Hospital and Monash University Melbourne Victoria Australia; ^5^ Department of Health Sciences University of South Australia Adelaide South Australia Australia

**Keywords:** crossmatch, Explex, Luminex, supplemental, transplantation

## Abstract

Current Luminex platforms cannot represent the totality of polymorphism in human HLA. Although supplemental, extended panels are available, they are not universally applied and their diagnostic value is uncertain. It is unclear whether standard bead kits can accurately predict the reactivity seen in extended panels. This study investigated, when using the standard panel alone, whether a closest related bead strategy or an eplet pattern strategy might predict reactivity seen in an extended panel kit (Explex). In the closest related bead strategy, each bead in the extended panel was compared to a standard kit bead in the same serotype and/or had the fewest amino acid differences. Using the closest bead method the standard bead panel predicts reactivity for the majority of beads in the Explex panel (30/54 for Class I and 16/24 for Class II). In the eplet pattern strategy, the reactivity of beads in the Explex panel was predicted from proposed eplet assignment of the standard panel. This strategy showed modest accuracy for predicting positive Explex beads, with greater accuracy for Class II (25/26) compared to Class I (49/62). This strategy had similar accuracy for highly sensitised cohorts (19/22 for Class II, and 27/32 for Class I) when patterns of eplet reactivity could be identified. We found that closely‐related beads and eplet patterns (when identifiable) can predict positive reactivity in unrepresented alleles with moderate success. However, we detail several exceptions where additional panel testing was found to be helpful. Our findings support selective use, rather than universal application of extended panels in our local population.

**Abbreviations**
AFA: African Americans
API: Asian and Pacific Islander
AXE: absorption with crossmatch cells and elution
CIWD: common intermediate and well‐documented
HIS: Hispanic
MECA: Middle Eastern and North Coast of Africa
MFI: median fluorescent intensity
NAM: Native American
NPV: negative predictive value
PPV: positive predictive value
*R*
: pearson correlation coefficient
*R* ^2^
: square of the pearson correlation coefficient

## 1 Introduction

Virtual crossmatch is commonly performed with multiplex single antigen bead Luminex technology, whereby HLA molecules are conjugated to fluorescent Luminex beads and anti‐HLA antibodies are detected with fluorescent anti‐human secondary antibodies. When combined with HLA typing of the organ donor and recipient, this is an efficient method of assessing histocompatibility. One limitation of multiplex Luminex bead assays is the restricted number of alleles that are represented in current standard bead kits which cannot cover the vast polymorphism present in HLA alleles. Although up to 98 different HLA antigens each for Class I and Class II are included in standard bead kits, several HLA alleles common in various populations are not included in standard kits. A comparison of standard bead kits revealed that 7.9%–11.8% of various ethnic populations did not have their HLA‐C typing represented on standard kits [1]. In addition, 4.0%–8.2% of HLA‐B alleles present in Middle Eastern and North Coast of Africa (MENA), Asian and Pacific Islander (API), and Native American (NAM) populations are not represented in standard kits [1]. Moreover, HLA‐DRB1 alleles were not represented in a significant minority of African Americans (AFA), NAM and Hispanic (HIS) populations [1].

The alleles that are not included in the standard single antigen bead kit are represented at a serological or allele group level by other beads in the kit. For example, while the HLA‐B*38:02 is not included in the One Lambda standard Class I single antigen bead Luminex kit, the HLA‐B*38:01 is included. The HLA‐B*38:02 allele is not common in the European Caucasoid populations but is present in approximately 5.8% of the Vietnamese, 4.0% of the Chinese populations in the National Marrow Donor Program registry, and in 1.8% of API populations in the Common Intermediate and Well‐Documented (CIWD) HLA alleles data set [2, 3]. If an antibody is directed to the entire B38 allele group, this would be detected by the HLA‐B*38:01 bead. However, on occasion, antibodies can be directed to epitopes that may not be shared by all alleles within an antigen group. Various methods have been applied to predict or explain these differences in immunogenicity, each with their own limitations. Amino acid alignment is commonly employed. For example, the B*38:01 allele has an isoleucine at the 80th amino acid residue, whereas the B*38:02 has a threonine. Another common approach is to evaluate eplets, or configurations of amino acids within a 3–3.5 Ångstrom radius, although many theoretical eplets remain unverified [4]. In this example, the antibody‐verified Bw4‐related 80I eplet [5] is present on HLA‐B*38:01 but not on HLA‐B*38:02. In contrast, the 80TLR antibody‐verified eplet [6] is present on the HLA‐B*38:02 but not on the HLA‐B*38:01. The 80I eplet is a frequent mismatch, occurring in 60.4% of mother–child pairs, and is also considered to be an immunogenic eplet, with antibodies formed in 33.3% of occasions when mother and child are eplet‐mismatched [7]. Individuals who develop an antibody to the 80I eplet would be predicted to react to HLA‐B*38:01, but not to HLA‐B*38:02. Therefore, a single antigen bead assay with only one of these beads may not distinguish between different patterns of reactivity for these two B38 alleles. However, a Luminex assay that contained both alleles could be of added value in this scenario, especially in ethnically diverse communities.

Currently, One Lambda offers an extended panel (Explex) with 54 additional Class I alleles and 24 additional Class II alleles which can be performed as a standalone assay or in combination with the standard bead panel. One Lambda also offers a supplementary kit which contains these additional alleles which can be run as a standalone assay. The addition of these beads has been found to increase the HLA‐C representation by 7%–12% in all ethnic groups [1]. It also increased the HLA‐B and HLA‐DR representation by 2%–8% and 3%–9%, respectively, for MENA, API, HIS and NAM ethnic groups [1]. Evaluation of supplemental kits has been previously performed by Oesegawa et al. to refine the definition of serotypes [8]. However, the focus of their study was in defining serotypes and crucial residues, and the correlation between supplemental beads and their standard bead counterparts was not delineated [8].

However, several questions remain unanswered. First, it is not known whether the additional alleles in the Explex kit are already adequately represented by their corresponding alleles in the same antigen group that are included in the standard kit. Second, it is also not known whether an eplet‐based evaluation of the standard bead panel would be adequate in predicting the reactivity to alleles in the Explex kit. If either of these strategies, or both strategies combined, were effective, it would make testing of these additional alleles unnecessary.

## 2 Material and Methods

### 2.1 Closest Related Bead Strategy

We selected a cohort of highly sensitised individuals for testing with a panel‐reactive antibody (PRA) > 95% that were on the renal transplant waiting list between November and December 2023 in New South Wales, Australia. This included patients from multiple ethnicities, including API and Aboriginal and Torres Strait Islander communities. This cohort was chosen based on the likelihood that this cohort would have antibodies to multiple targets, permitting more correlation data to be collected from the same number of specimens, as compared to unsensitised or lowly sensitised individuals. In addition, this cohort is likely to have real reactivity from sensitisation and benefit most from a refined assessment of additional alleles, especially if this led to new acceptable antigens that may permit transplantation. A highly sensitised cohort was also thought to have a greater chance of developing antibodies that might distinguish between alleles within antigen groups, and therefore we felt that this was the best population to evaluate first as proof‐of‐concept. For this group, we confined our analysis to paired standard and Explex specimens who had similar positive control/negative control ratios, excluding those with ratios that differed by greater than log2. This was done to minimise error that could be due to interassay variability. Finally, a Pearson Correlation *R* and *R* ^2^ were calculated for each pair of beads. In addition, calculations were performed to determine how well the standard bead predicted the result of the Explex bead, including statistics for sensitivity, specificity, negative predictive value and positive predictive value. Results were considered to be positive if the MFI was greater than 4000, a negative result was an MFI < 1000, and indeterminate results were results between 1000 and 4000 and were excluded from the analysis, in a strategy similar to that applied by Osoegawa et al. [8].

### 2.2 Eplet Pattern Strategy

Class I and Class II eplets were accessed from the HLA Eplet Registry http://www.epregistry.com.br on 31st August 2023 and reflect the eplet data uploaded on May 14, 2020. A short‐list of antibody‐verified eplets that split traditional allele groups was chosen for further evaluation. The 156DA was not included in our analysis as we and others have noted this reactivity could be attributed to denatured proteins [9]. All single antigen bead samples tested at the New South Wales Transplantation and Immunogenetics Laboratory between 1st May 2023 and 1st September 2023 that had reactivity to short‐listed antibody‐verified eplets were selected for further testing with Explex. We assessed the accuracy of possible eplet patterns derived from the standard kit in predicting bead reactivity on the Explex kit. We also assessed the ability to predict an unacceptable Explex allele at 2000 and 4000 MFI cutoffs, with an eplet considered positive at each threshold if the majority of beads were greater than either of these respective MFIs. Although these MFI cutoffs are higher than applied worldwide, these thresholds were chosen based on local Australian cutoffs for living and deceased donor renal transplantation. This strategy was also adapted for the highly‐sensitised cohort of patients on the renal transplant list, with eplets defined by the presence of positive beads and the absence of pertinent negative beads on the standard panel.

### 2.3 Luminex Testing

Single antigen bead testing was performed using LABScreen beads (LS1A04, SAG1 Lot 14; LS2A01 SAG2, Lot 16). Explex testing was performed separately using Explex beads (LS1AEX01 Lot 6; LS2AEX01 Lot 6). Both assays were performed according to modified manufacturer's specifications (One Lambda, Canoga Park, California, USA). In brief, 2.5 μL 50 mM EDTA was added to each well in a 96 well plate, with 20 μL of serum and 2.5 μL of LABScreen beads. The plates were sealed, incubated for 30 min at room temperature on a plate shaker. After incubation, the plates were washed 3 times with wash buffer. 100 μL of PE‐conjugated goat anti‐human IgG antibody was added to each well and incubated, sealed, and incubated in the dark for 30 min on a plate‐shaker. The plates were then washed twice before resuspending in 80 μL of PBS and run on the Luminex FlexMAP3D systems (Luminex Corporate, Austin, Texas, USA). Pre‐treatment of serum specimens with 2.5 μL of Adsorb (One Lambda) was used as required if the MFI of the negative control bead was higher than 500. Each specimen was assessed using HLA Fusion Software 4.6 (One Lambda). Standard quality control criteria with co‐efficient of variation (CV) check was performed for each bead and patient.

### 2.4 Absorption Elution Experiments

Absorption with crossmatch cells and elution experiments (AXE) were performed similar to prior publications [9]. Briefly, buffy coats were prepared from whole blood of previously typed individuals. Washed cells were incubated with 200 μL of EDTA‐treated patient sera. Cells and sera were vortexed gently and placed in a 37° heat block for 30 min. After 30 min, the cells were washed 6 times. 100 μL supernatant from the last wash was reserved for Luminex testing in parallel. With the dry cell pellet, 50 μL of acid solution was added (Gamma ELU‐KIT II, 0007861, Immucor, Norcross, Georgia, USA) and incubated for 1 min at room temperature, immediately spun at 1500G for 1 min, and the supernatant was removed. 50 μL of base solution (Gamma ELU‐KIT II) was added to the supernatant. 5 μL of either acid or base solution is added until pH is 6.8–7.2.

The typings for the target cells used in the AXE were as follows. For the 138K eplet, the target cell typing was A*32:01, B*14:01, C*08:02, DRB1*07:01, DQB1*02:02, DQA1*02:01, DPB1*04:01, DPB1*02:02, DPA1*01:03, DPA1*02:02 and DRB4*01:01. For the 80I eplet, the target cell typing was A*01:01, A*02:11, B*38:01, B*40:06, C*12:03, C*15:02, DRB1*14:01, DRB1*15:01,DQB1*05:03, DQB1*06:01, DQA1*01:03, DQA1*01:04, DPB1*02:01, DPB1*104:01, DPA1*01:03, DRB3*02:24 and DRB5*01:01. For the q57V eplet, the first target cell typing was A*29:01, A*33:03, B*07:05, B*37:01, C*06:02, C*15:05, DRB1*10:01, DQB1*05:01, DQA1*01:05, DPB1*02:01, DPA1*01:03. The second target cell typing was A*03:01, A*68:01, B*07:02, B*44:02, C*07:02, C*07:04, DRB1*01:01, DQA1*01:01, DQB1*05:01, DPA1*01:03, DPB1*04:01 and DPB1*04:02. For the 158T eplet, the first target cell typing was A*02:01, A*03:01, B*07:02, B*39:01, C*07:02, C*12:03, DRB1*01:01, DRB1*08:01, DQB1*04:02, DQB1*05:01, DQA1*01:01, DQA1*04:01, DPB1*02:01, DPB1*03:01, DPA1*01:03. The second target cell typing was A*03:01, A*11:01, B*39:06, B*55:01, C*01:02, C*12:03, DRB1*04:03, DRB1*07:01, DQB1*02:02, DQB1*03:02, DQA1*02:01, DQA1*03:01, DPB1*04:01, DPB1*104:01, DPA1*01:03, DRB4*01:03. For the 177DT eplet, the first target cell typing was A*30:04, A*32:01, B*41:01, B*50:01, C*06:02, C*07:01, DRB1*05:01, DRB1*10:01, DQB1*03:01, DQB1*05:01, DQA1*01:05, DQA1*05:05, DPB1*04:01, DPB1*19:01, DPA1*01:03, DPA1*02:07, DRB3*02:02. The second target cell typing was A*02:01, A*02:06, B*41:02, B*51:01, C*16:02, C*17:03, DRB1*04:01, DRB1*07:01, DQB1*02:02, DQB1*04:02, DQA1*02:01, DQA1*03:03, DPB1*02:01, DPB1*17:01, DPA1*01:03, DPA1*02:01, DRB4*01:01, DRB4*01:03. For the potential 11AV eplet, the target cell typing was A*11:01, B*13:01, B*15:01, C*04:03, DRB1*04:05, DRB1*16:02, DQB1*04:02, DQB1*05:02, DQA1*01:02, DQA1*03:03, DPB1*01:01, DPB1*05:01, DPA1*02:02, DRB4*01:03, DRB5*01:01. For the 55EAE, the target cell typing was A*02:01, A*24:02, B*38:02, B*40:06, C*07:02, C*08:01, DPB1*05:01, DPA1*02:01, DPA1*02:02, DQB1*03:01, DQA1*05:05, DQA1*06:01, DRB1*12:01, DRB3*02:02, DRB3*03:01. For the 131S/109L eplet, the target cell typing was A*24:02, A*30:02, B*07:02, B*55:01, C*03:03, C*15:02, DRB1*03:01, DRB1*08:01, DQB1*02:01, DQB1*04:02, DQA1*04:01, DQA1*05:01, DPB1*04:01, DPA1*01:03, DRB3*01:01.

### 2.5 Assessment of Antigen Density

The antigen density data was taken from worksheets for LS1A04 (SAG1 Lot 14), LS1AEX01 (Lot 6), LS2A01 SAG2 (Lot 16) and LS2AEX01 (Lot 06). Standard tests for normality (D'Agnostino & Pearson, Anderson‐Darling, Shapiro–Wilk and Kolmogorov–Smirnov) were performed. Mann–Whitney tests for non‐parametric data were used to compare the antigen density between corresponding Class I and Class II kits.

## 3 Results

Closest Related Bead Strategy: Predicting reactivity to alleles on the Explex kit based on the reactivity of the most closely related bead on the standard kit.

Our first approach was to determine whether reactivity to alleles in the Explex kits could be predicted by looking at their most closely related bead in the same antigen group or serotype that is already included in the standard bead kit. This was investigated in a group of 66 highly sensitised patients. To ensure that differences in MFI were not due to interassay variability, we excluded samples that had a greater than log2 difference in positive/negative control bead MFI ratios between the standard and Explex kit. This narrowed our correlation analysis to 48/66 samples for Class I, and 50/66 samples for Class II. The median age of the cohort was 51.5, with an interquartile range of 41–58, and age range between 23 and 74. In total, 51.52% were female, 36.36% were sensitised by prior pregnancy, 80.30% were sensitised by prior transplant, 65.15% had sensitisation by transfusion, and the sensitisation history was not known in 3.03% of patients (Tables S7 and S8). The standard bead kits predicted the reactivity of the majority (30/54 for Class I and 16/24 for Class II) beads in the Explex kit (Tables 1, 2, 3, 4, 5, 6, Tables S1–S6). Reviewing beads with lower correlation between beads on the standard and Explex panel revealed the following differences.

**TABLE 1:** Correlation between A locus Explex beads and their closest related bead on the standard panel.

Standard bead	Explex bead	Pearson correlation *R* ^2^	Sensitivity (%)	Specificity (%)	PPV (%)	NPV (%)	Indeterminate (%)
A*01:01	A*01:02	0.98	100	100	100	100	18.75
A*02:01	A*02:05	0.9	**94.74**	100	100	**96.15**	8.33
A*02:01	A*02:07	0.97	100	100	100	100	10.42
A*02:01	A*02:10	**0.86**	**94.44**	100	100	**96.3**	8.33
A*02:01	A*02:18	**0.79**	**88.89**	100	100	**92.31**	12.5
A*03:01	A*03:02	0.98	100	100	100	100	16.67
A*26:01	A*26:02	0.98	100	100	100	100	22.92
A*26:01	A*26:03	0.96	**92.31**	100	100	**96.43**	16.67

*Note:* Sensitivity, specificity, PPV and NPV values < 100%, and correlation values < 0.9 are indicated in bold.

Abbreviations: NPV, negative predictive value; PPV, positive predictive value.

**TABLE 2:** Correlation between B locus Explex beads and their closest related bead on the Standard Panel.

Standard bead	Explex bead	Pearson correlation *R* ^2^	Sensitivity (%)	Specificity (%)	PPV (%)	NPV (%)	Indeterminate (%)
B*07:02	B*07:14	0.99	100	100	100	100	18.75
B*15:01	B*15:04	0.96	100	100	100	100	18.75
B*15:01	B*15:06	0.98	100	100	100	100	16.67
B*15:01	B*15:07	0.97	100	100	100	100	16.67
B*15:16	B*15:17	0.96	100	100	100	100	29.17
B*15:10	B*15:18	0.95	100	100	100	100	22.92
B*15:01	B*15:20	0.92	**92.86**	100	100	**96.00**	20.83
B*15:02	B*15:21	0.97	100	100	100	100	18.75
B*15:01	B*15:24	**0.67**	**84.62**	100	100	**92.31**	22.92
B*15:01	B*15:27	0.99	100	100	100	100	16.67
B*27:05	B*27:04	0.94	100	100	100	100	29.17
B*27:05	B*27:06	0.94	100	100	100	100	22.92
B*35:01	B*35:02	0.96	100	100	100	100	14.58
B*35:01	B*35:03	0.99	100	100	100	100	12.50
B*35:01	B*35:08	0.99	100	100	100	100	14.58
B*35:01	B*35:12	0.96	100	100	100	100	14.58
B*38:01	B*38:02	**0.88**	100	**95.45**	**92.86**	100	27.08
B*39:01	B*39:02	**0.78**	100	100	100	100	25.00
B*39:01	B*39:04	0.94	**91.67**	100	100	**96.15**	22.92
B*39:01	B*39:05	0.94	100	100	100	100	25.00
B*39:01	B*39:06	**0.82**	100	100	100	100	31.25
B*39:01	B*39:13	**0.67**	100	**96.15**	**91.67**	100	22.92
B*40:02	B*40:03	0.98	100	100	100	100	18.75
B*40:02	B*40:04	0.95	100	100	100	100	18.75
B*50:01	B*40:05	0.92	100	**95.83**	**92.86**	100	22.92
B*41:01	B*41:02	0.93	**94.44**	100	100	**94.74**	25.00
B*42:01	B*42:02	0.96	100	100	100	100	25.00
B*15:03	B*48:02	0.90	**90.00**	**96.43**	**90.00**	**96.43**	20.83
B*45:01	B*50:02	0.97	100	100	100	100	10.42
B*55:01	B*55:02	0.95	100	100	100	100	29.17
B*55:01	B*55:04	**0.87**	**92.86**	100	100	**95.24**	29.17
B*56:01	B*56:03	0.93	100	**93.10**	**85.71**	100	14.58

*Note:* Sensitivity, specificity, PPV and NPV values < 100%, and correlation values < 0.9 are indicated in bold.

Abbreviations: NPV, negative predictive value; PPV, positive predictive value.

**TABLE 3:** Correlation between DR locus Explex beads and their closest related bead on the standard panel.

Standard bead	Explex bead	Pearson correlation *R* ^2^	Sensitivity (%)	Specificity (%)	PPV (%)	NPV (%)	Indeterminate (%)
DRB1*04:03	DRB1*04:06	0.95	100	100	100	100	28
DRB1*04:03	DRB1*04:07	0.96	100	100	100	100	28
DRB1*04:01	DRB1*04:10	0.96	100	100	100	100	26
DRB1*04:03	DRB1*04:11	0.92	100	100	100	100	26
DRB1*08:01	DRB1*08:02	0.98	100	100	100	100	14
DRB1*08:01	DRB1*08:03	0.98	100	100	100	100	18
DRB1*08:01	DRB1*08:07	0.98	100	100	100	100	14
DRB1*13:01	DRB1*13:02	0.96	100	100	100	100	14
DRB1*14:02	DRB1*14:03	**0.80**	**92.86**	**96.55**	**92.86**	**96.55**	14
DRB1*14:01	DRB1*14:04	0.94	100	**92.86**	**89.47**	100	10
DRB1*14:01	DRB1*14:05	**0.88**	100	**92.86**	**88.24**	100	14
DRB1*14:02	DRB1*14:06	0.97	100	100	100	100	12
DRB3*02:02	DRB3*02:01	0.99	100	100	100	100	14
DRB5*01:01	DRB5*01:02	0.96	100	100	100	100	12

*Note:* Sensitivity, Specificity, PPV and NPV values < 100%, and Correlation values < 0.9 are indicated in bold.

Abbreviations: NPV, negative predictive value; PPV, positive predictive value.

**TABLE 4:** Correlation between DQ locus Explex beads and their closest related bead on the standard panel.

Standard bead	Explex bead	Pearson correlation *R* ^2^	Sensitivity (%)	Specificity (%)	PPV (%)	NPV (%)	Indeterminate (%)
DQB1*03:01 DQA1*02:01	DQB1*03:19 DQA1*02:01	0.98	100	100	100	100	16
DQB1*05:01 DQA1*01:01	DQB1*05:03 DQA1*01:01	0.86	100	**96.88**	**90.91**	100	16

*Note:* Sensitivity, specificity, PPV and NPV values < 100%, and Correlation values < 0.9 are indicated in bold.

Abbreviations: NPV, negative predictive value; PPV, positive predictive value.

**TABLE 5:** Correlation between C locus Explex beads and their closest related bead on the standard panel.

Standard bead	Explex bead	Pearson correlation *R* ^2^	Sensitivity (%)	Specificity (%)	PPV (%)	NPV (%)	Indeterminate (%)
C*01:02	C*01:03	0.96	**85.71**	**96.88**	**85.71**	**96.88**	18.75
C*02:02	C*02:10	0.93	100	100	100	100	27.08
C*04:01	C*04:03	**0.84**	**90.91**	100	100	**96.97**	10.42
C*07:02	C*07:01	0.91	**87.50**	100	100	**96.77**	20.83
C*07:02	C*07:04	**0.85**	100	100	100	100	25.00
C*08:01	C*08:02	**0.52**	**60.00**	100	100	**89.19**	10.42
C*08:01	C*08:03	0.93	100	100	100	100	8.33
C*08:01	C*08:04	**0.49**	**54.55**	100	100	**86.49**	10.42
C*12:03	C*12:02	0.91	100	100	100	100	16.67
C*14:02	C*14:03	0.94	**75.00**	100	100	**93.33**	25.00
C*15:02	C*15:05	0.97	100	100	100	100	12.50
C*16:01	C*16:02	**0.28**	**50.00**	100	100	**83.33**	12.50
C*17:01	C*17:03	0.96	100	100	100	100	12.50
C*18:02	C*18:01	0.92	100	100	100	100	4.17

*Note:* Sensitivity, specificity, PPV and NPV values < 100%, and correlation values < 0.9 are indicated in bold.

Abbreviations: NPV, negative predictive value; PPV, positive predictive value.

**TABLE 6:** Correlation between DP locus Explex beads and their closest related bead on the standard panel.

Standard bead	Explex bead	Pearson correlation *R* ^2^	Sensitivity (%)	Specificity (%)	PPV (%)	NPV (%)	Indeterminate (%)
DPB1*02:01 DPA1*01:03	DPB1*02:02 DPA1*01:03	**0**	**0**	**90.00**	**0**	**97.30**	18
DPB1*01:01 DPA1*01:03	DPB1*26:01 DPA1*03:01	0.97	100	100	100	100	14
DPB1*13:01 DPA1*02:01	DPB1*30:01 DPA1*02:01	**0.83**	**80.00**	100	100	**97.56**	10
DPB1*01:01 DPA1*01:03	DPB1*31:01 DPA1*03:01	0.97	100	100	100	100	24
DPB1*04:01 DPA1*01:03	DPB1*40:01 DPA1*01:05	**0.11**	**0**	100	NA	**95.45**	12
DPB1*01:01 DPA1*01:03	DPB1*85:01 DPA1*01:03	0.97	100	100	100	100	18
DPB1*04:02 DPA1*01:03	DPB1*105:01 DPA1*02:01	**0.56**	**75.00**	**97.30**	**75.00**	**97.30**	18
DPB1*13:01 DPA1*02:01	DPB1*107:01 DPA1*02:01	0.98	100	100	100	100	10

*Note:* Differences between DPB1*04:02/DPA1*01:03 and DPB1*105:01/DPA1*02:01 are attributed to the difference in serological specificity between DPA1*01:03 and DPA1*02:01. Sensitivity, specificity, PPV and NPV values < 100%, and correlation values < 0.9 are indicated in bold.

Abbreviations: NPV, negative predictive value; PPV, positive predictive value.

**FIGURE 1:**
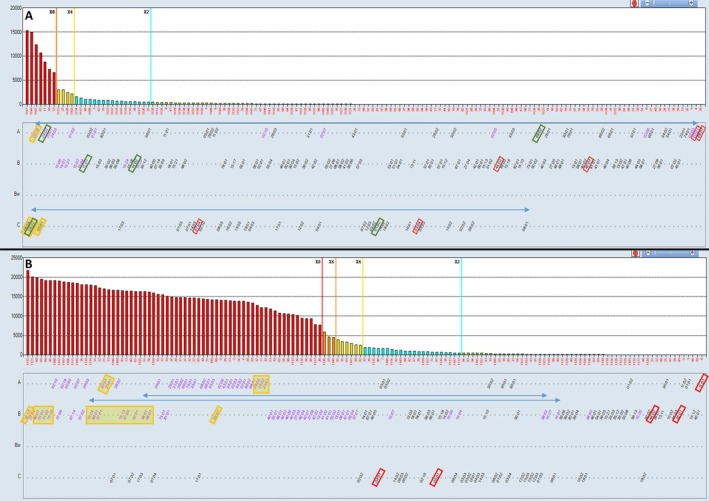
(A) Patient typed as A*02:01 was immunised against the C*08:02, and developed antibodies to the 138K antibody‐verified eplet (yellow). This is expressed on the Cw5 group that includes the C*05:01 on the standard panel, as well as the C*08:02, C*08:04 and A*02:18 (positive on the Explex panel). Self‐typing (red), typing of immuniser (green). The difference between the C*08:01 (negative on standard panel) and C*08:02 (positive on Explex panel) is indicated with a blue arrow. (B) Patient typed as B*44:02 (Bw4 80T expressing allele) and B*35:01 (Bw6), has developed antibodies to the Bw4 80I expressing group (yellow). This led to differing reactivity to B*38:01 (positive on the standard panel) and B*38:02 (negative on Explex panel), and B*15:24 (positive on Explex panel) and B*15:01 (negative on standard panel) with differences indicated by blue arrows. Self‐typing (red).

At the A‐locus, the most common difference was between the A*02:18 and the A*02:01, where the A*02:18 was positive and the A*02:01 was negative (*n* = 2), or when the difference in MFI between the two alleles was over 20,000 MFI (*n* = 1) (Figure S1). The difference between these two alleles is a lysine as opposed to a methionine in the exposed 138AA residue (K138 vs. M138). Although the A*02:18 is not frequent, the K138 is expressed by common HLA‐C alleles, particularly the Cw5 group, C*08:02 and C*08:04. In all three cases the differences between the A*02:18 and A*02:01 was explained by the patient being immunised to the C*08:02 either from a pregnancy (*n* = 1), prior transplant (*n* = 1), or immunised to the C*05:01 due to a prior transplant (*n* = 1) (Figure 1A, Figure S10). In 2 of 3 cases, the A*02:01 was also self‐typing. Another individual who was A*02:01‐positive also developed antibodies to A*02:05 and A*02:10 (Figure S11). An absorption elution experiment (AXE) was performed against the C*08:02, which confirm the 138K eplet and the differences in the MFI and elution efficiency for the alleles in the A2 and Cw8 antigen group, with elution MFIs ranging from 2667 to 5336 and elution efficiencies ranging from 21.5% to 42.3% (Figure S19). The only other difference in the A locus was an individual who developed differing reactivity between A*26:01 (negative) and A*26:03 (positive), which appeared to be due to an antibody to an unverified eplet (76VDT) shared with an A*02:05 prior transplant mismatch (Figure S11).

**FIGURE 2:**
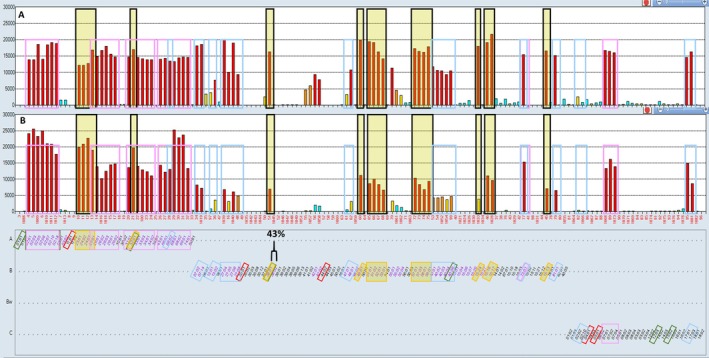
AXE confirms 80I antibody‐verified eplet from Figure 1B. 80I is shown in yellow and adsorbed on surrogate target cell (typed as B*38:01). The self‐typing (red) and the typing of the target cell used for the AXE protocol (green) are indicated. The remaining reactivity in the eluate is explained by a second antibody‐verified eplet (253Q, pink) shared with the target cell A*02:11, and a third antibody‐verified eplet (163EW, blue), shared with the target cell B*40:06 typing. Of note, the B*38:01 only expresses the 80I, and not the 253Q and 163EW. The 80I includes the B*38:01 (positive on standard panel) but not the B*38:02 (negative on Explex panel) and this difference is indicated with a bracket. Although antibody binding was noted to the B*27:04, B*27:05 and B*27:06 which express the 80T eplet, binding to the 80T eplet was not evident on other beads (A*01:01, A*01:02, A*11:01 (self), A*11:02, B*44:02 (self) and B*44:03). Instead, this binding to the B*27:04, B*27:05 and B*27:06 appeared to match better with the 163EW eplet shared with the target B*40:06. (A) The initial serum sample. (B) The MFI of the B*38:01 is 6967 and the elution efficiency (percentage MFI of eluate compared to initial serum sample MFI) is 43%. The average MFI and elution efficiency of the 19 alleles expressing the 80I is 11614 and 61%.

For the B‐locus, one key difference between the standard and Explex panels was between alleles that have either the 80T and 80I residues. The 80I or 80T residues subdivide the Bw4 group and are thought to be important killer cell immunoglobulin‐like receptor (KIR) ligands [10]. The Bw4‐expressing B38 associated split can be divided into 80I‐expressing alleles (B*38:01) and 80T‐expressing alleles (B*38:02); this can lead to strong reactivity to the B*38:01 but not to the B*38:02 (Table 2, Figure 1B, Figure S2). The same amino acid location was also associated with poor correlation between the B*15:01 and the B*15:24 (Table 2, Figures S2 and S12) because, while both belong to the B62 associated split, the B*15:24 is part of the Bw4 80I group, whereas the rest of the B62 are part of the Bw6 group. Therefore, differences between the B*15:01 and B*15:24 occurred in individuals who were either Bw6 homozygotes or who expressed a Bw4 with an 80T as opposed to an 80I residue. The 80I eplet which distinguishes between the B*38:01 and B*38:02 was confirmed by AXE with a cell type expressing B*38:01 with an MFI of 6967 and elution efficiency of 43% (Figure 2). No antibodies to B*38:02 were eluted. The residual reactivity (percentage MFI of last wash control compared to initial serum sample MFI) for the B*38:01 was 0% (Figure S18).

Other differences in the B‐locus were attributed to unverified eplets including the 63NI, and 103L (Figures [Link], [Link], and S14). Differing reactivity was also seen within the B39 associated split, with 2.5 to 4‐fold differences in MFI between the B*39:01 and the B*39:02, and the B*39:01 and the B*39:06, in some individuals resulted in lower correlation compared to other bead pairs (Table 2, Figures S3 and S12). One sera specimen showed differences in MFI between the B*39:01 (strong, MFI 11703) and B*39:06 (questionable, MFI 2756) which was found to react to the 158T (antibody‐verified) eplet when absorbed and eluted against a B*39:01‐expressing cell (Figure S20). Of interest, when absorbed and eluted against a B*39:06‐positive target, the eluate to the 158T was detectable, but at a significantly lower MFI (MFI) (Figure S21), suggesting that in this allosera against the 158T the avidity for the B*39:01 is higher than for the B*39:06. A difference was also noted in one individual who reacted to the B*39:04 but not to the rest of the B39 antigen group, with an antibody reactivity pattern to the 12M (non‐verified) Eplet (Table 2, Figures S2 and S17). In one sera sample, a difference in MFI was noted for the B*41:02 (MFI 4744), and a B*41:01 (MFI 850). Two absorption elution experiments against a B*41:01‐positive cell and a B*41:02‐positive cell, respectively confirmed binding and elution of the 177DT (unverified) eplet which includes both the B*41:02, B*41:01 but also includes the B*08:01, the B*42:01 and the B*42:02 (Figures S22 and S23). In all three investigations (initial sera and 2 AXEs), the antibody to B*41:01 was detectable, although the MFI was significantly lower than the MFI of the antibody to B*41:02 (Figure S24).

**FIGURE 3:**
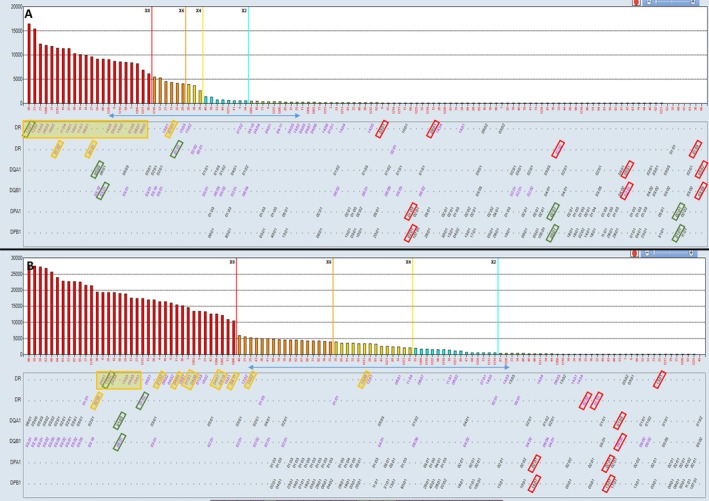
(A) Patient immunised against DRB1*12:01 has developed antibodies to the 70D antibody‐verified eplet (yellow) which includes the DRB1*14:03 but not the DRB1*14:02 or other DR14 alleles. Self‐typing (red), immunising alleles (green). The difference between the DRB1*14:03 (positive on Explex panel) and DRB1*14:02 (negative on standard panel) is indicated with a blue arrow. (B) Patient typed as DRB1*13:01 was immunised against DRB1*15:02, and developed antibodies to the 70QT antibody‐verified eplet (yellow) including the DRB1*14:02 and DRB1*14:06 but not the DRB1*14:03 or other DR14 alleles. The difference between the DRB1*14:03 (negative on standard) and DRB1*14:02 (positive on Explex panel) is indicated with a blue arrow.

The additional DRB1 beads in the Explex panel correlated well with the beads in the standard panel, with the exception of DRB1*14:03, DRB1*14:04 and DRB1*14:05 (Table 3, Figure S5) with differences relating to the antibody‐verified 11STS eplet and the 70R/70D/70QT eplets. The DR1404 is an associated split, and two DRB1*14:04 (70R)‐positive individuals developed antibodies to the alleles that share the 11STS eplet, which includes the remaining DR14 alleles on the panels, as well as the 70D with heightened reactivity to the DRB1*14:03 (Figure S14). The reactivity to the 70D was also seen in individuals who were not DR14‐positive, such as a patient who was DRB1*03:01 (70Q) and DRB1*09:01 (70R) (Figure 3A). Two DRB1*13:01 (70D) homozygotes developed antibodies to alleles that express the 70QT (antibody‐verified) eplet, including DRB1*14:02 and DRB1*14:06 (Figure 3B). In summary, differences in DR reactivity in the Explex and standard kit were seen with DRB1*14:03, DRB1*14:04 and DRB1*14:05, due to reactivity patterns expected of associated splits and antibody‐verified eplets within the DR14 group.

**FIGURE 4:**
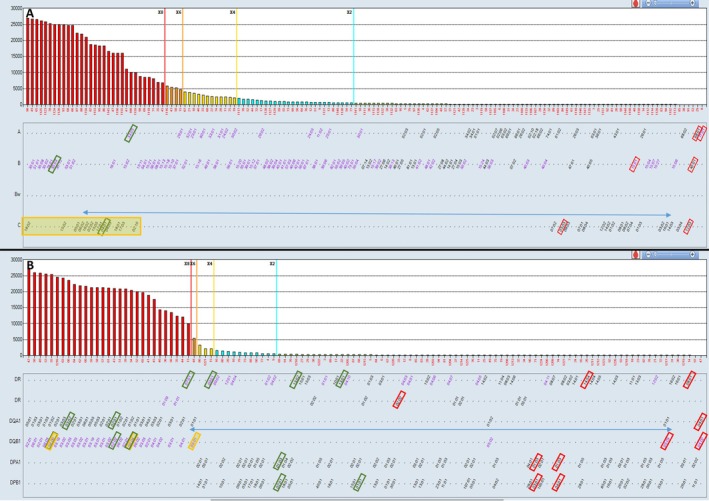
(A) Patient immunised against C*04:01 has developed antibodies to alleles that share the 80K antibody‐verified eplet (yellow), including the C*16:02 and not the C*16:01. Self‐typing (red), immunising alleles (green). The difference between the C*16:02 (positive on the Explex panel) and C*16:01 (negative on the standard panel) is indicated with the blue arrow. (B) Patient who is typed as DQB1*05:03 has developed an antibody to alleles that share the q57V antibody‐verified eplet (yellow) that includes the DQB1*05:01, but not the DQB1*05:03. Self‐typing (red), immunising alleles (green). The difference between the DQB1*05:01 (positive on the standard panel) and DQB1*05:03 (negative on the Explex panel) is indicated with a blue arrow. This patient had 2 prior transplants, and while DQ typing from the initial transplant is missing, given the donor's DR13 typing, the patient was likely to be immunised against either a DQB1*06:03, DQB1*06:04 or DQB1*06:09.

**FIGURE 5:**
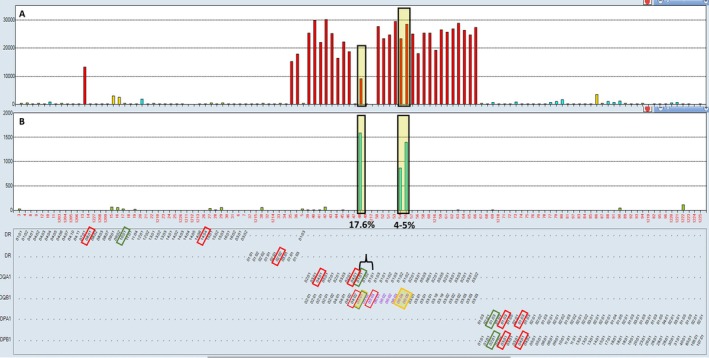
AXE confirms the q57V antibody‐verified eplet from Figure 4B. Antibody to the q57V (yellow) is adsorbed on the donor cell DQB1*05:01/DQA1*01:01 typing. The self‐typing is in red and the typing of the target cell used for the AXE protocol is in green. Of note, only the DQB1*05:01 elutes, not the DQB1*05:02 or DQB1*05:03 (which do not express the q57V) and the difference in indicated with a bracket. Although the eluate MFI for DQB1*06:04 and DQB1*06:09 is similar to the DQB1*05:01 (454, 528 and 512, respectively), the elution efficiency is lower because an additional antibody targeting the DQ6 antibody is likely also present in the original sera (thus leading to a lower elution efficiency). (A) The initial serum sample. (B) The elution efficiency (percentage MFI of eluate compared to initial sample MFI) is 17.6%.

The Explex kit has two additional DQ beads. The DQB1*05:03 was useful in a single case in our cohort (Table 4, Figure S6). This individual was DQB1*05:03‐positive, and developed an antibody to the DQB1*05:01, after sensitisation with a DQ6 allele and due to an antibody targeting the q57V (antibody‐verified) eplet (Figure 4B). This eplet was confirmed by AXE against 2 surrogate donor cells expressing the DQB1*05:01/DQA1*01:01, with an MFI of 1591 and elution efficiency of 18% (Figure 5). The other DQ5 alleles did not absorb or elute and the residual reactivity in the last wash was 0% (Figure S18). This q57V eplet confirmed again with a second AXE DQB1*05:01/DQA1*01:01, and had an MFI of 512 and elution efficiency of 9% upon retesting on Luminex (Figure S25). Again, the other DQ5 alleles did not absorb/elute and the residual activity in the last wash was 0%.

At the C‐locus, the closest related bead strategy had difficulty in predicting reactivity to Cw16 and Cw8 alleles. The C‐locus is divided into two major groups, the HLA‐C1 with an amino acid sequence from amino acids 76–83 of VSLRNLRG, and HLA‐C2 with an amino acid sequence VNLRKLR, which have differing specificity for the KIR2DL2/3, and KIR2DL1, respectively [11] and these HLA‐C1/HLA‐C2 groupings distinguish between the C*16:01 (HLA‐C1) and the C*16:02 (HLA‐C2). In 6/12 cases when the C*16:01 was positive, the C*16:02 was also positive with an antibody targeting the whole Cw16 antigen group (Table 5, Figure S8). However, in the other 6/12 cases, the C*16:02 was negative. In these cases, the patient's C‐locus alleles were in the HLA‐C1 group, and they were immunised with a C‐locus typing that belonged the HLA‐C2 group (Figure 4A, Figure S15). Apart from Cw16, the standard kit had limitations in predicting the reactivity to Cw8 alleles (Table 5, Figure S8). This is because of the 138K residue (discussed above in relation to the A*02:18), which distinguishes the Cw5, C*08:02 and C*08:04, from the C*08:01.

A low‐level antibody to the C*07:01 allele was detected in one patient whose self‐typing was C*07:02 after receiving a C*07:01‐positive transplant (Figure S16). One other patient whose self‐typing was C*04:01 developed an antibody to the C*04:03. This reactivity was confirmed on an AXE (Figure S26), with the C*04:03 having an elution efficiency of 40% (MFI 4678) and the C*04:01 having an elution efficiency of 0% (MFI 0). For other patients, variability was seen in other alleles (C*01:02/C*01:03 and C*14:03/C*14:02) that was not explained by known immunisation or eplet mismatches, and raised the possibility that some variability could be related to spurious beads activity [12].

The DPB1*02:01 was inaccurate in predicting the DBP1*02:02 reactivity because the two are separate serotypes and, as anticipated, there was no concordance between these two alleles (Table 6, Figure S9). Reactivity to DPB1*02:02 occurred in one individual who developed antibodies to the 55EAE eplet (Figure S16). This patient also had antibodies to DPB1*30:01 but not to the DPB1*13:01 (which was self), although they are considered to be in the same serotype [8]. This was confirmed by AXE against a cell expressing DPB1*30:01 with a post‐elution MFI of 4315 (elution efficiency 24.7%) compared to the DPB1*13:01 which had a post‐elution MFI of 0. Minimal residual reactivity was seen in the last wash (Figure S28). Although poor correlation was also identified between DPB1*04:01 and DPB1*40:01, neither allele had a reactivity that was greater than 5000 MFI, and additional results would be required to confirm this. Finally, any differences noted between the DPB1*04:02/DPA1*01:03 and DPB1*105:01/DPA1*02:01 beads were likely explained by antibodies to the differing DP alpha chains on the beads.

We subsequently evaluated the difference in target antigen densities on the beads in the standard and Explex kits to determine any potential effect on our results. In general, the median antigen density for the beads in the standard kit LS1A04 SAG1 Lot 14 was lower (23,840) when compared to LS1AEX01 Lot 6 (26,466) (*p* < 0.0001). The median antigen density for the beads in LS2A01 SAG2 lot 16 was substantially higher (27,334) when compared to LS2AEX01 Lot 6 (18,120) (*p* < 0.0001). Some intra‐kit variability in antigen density existed within all four kits. This can contribute to a bias or systemic error and affect the line of best fit in correlation figures but does not affect the *R* and *R* ^2^ statistics. For example, the line of best fit for C*07:02 and C*07:04 (Figure S7) shows stronger binding to C*07:02 compared to C*07:04, which may reflect the antigen density of C*07:02 (21,520) compared to C*07:04 (15,368). However, differences in antigen density did not explain the variable reactivity among alleles in the B39 allele group; the B*39:01, B*39:02, B*39:04, B*39:05, B*39:06, B*39:13 had an antigen density corresponding with 24,113, 27,262, 27,394, 28,035 and 27,158, respectively.

In summary, the standard beads kit displayed good correlation, sensitivity and specificity in predicting the reactivity of most beads in the Explex kit when using a closest related bead strategy. However, several Explex beads reactivities were not accurately predicted based on the standard kit alone. The alleles on the Explex kit that were thought to be most informative in our population include the DRB1*14:04, DRB1*14:05, DRB1*14:03, DQB1*05:03, B*38:02, DPB1*02:02, C*08:02 and C*16:02.

### 3.1 Eplet Pattern Strategy‐Predicting Reactivity to Alleles on the Explex Kit Based on Eplet Analysis of the Standard Kit

Many differences between the closest related beads could be predicted based on antibody‐verified eplets. As such, an evaluation of all antibody‐verified eplets was performed looking for eplets which were predicted to split antigen groups or serotypes. 17 such Class I eplets were identified that were predicted to split antigen groups or serotypes (Table S9). The B*48:02 was grouped with the B72 associated split based on recent re‐classification [8]. Of the 79 antibody‐verified Class II eplets evaluated, 15 eplets were identified that had a pattern of reactivity that also split serotypes (Table S10). A further eight DP eplets also had a pattern of reactivity which would be expected to include alleles that are not included in the standard panel of beads (Table S11). However, only six of these included DP alleles that were in different P‐groups from the beads in the standard panel.

We evaluated these eplets (Tables S9–S11) to determine if an eplet pattern of reactivity on a standard bead panel could predict the likely outcome for an allele on the extended panel. We reviewed all standard SAB Class I and Class II runs performed in the New South Wales Transplantation and Immunogenetics Laboratory between 1st May 2023 and 1st September 2023, a total of 2087 unique samples. Samples that evidenced possible reactivity to the eplets highlighted above were chosen for further testing. 62 samples which had a reactivity pattern consistent with these 18 Class I eplets were identified. Despite each of these eplets being antibody‐verified, no samples were identified that exhibited reactivity to a 65QKR, 71TTS, or 193PV Class I eplets. There was only one example of a potential 80TLR and 177KT, and only two examples of potential 21H, 80N and 138K, respectively. The most common Class I eplets that were predicted to split alleles within an allele group were the 80K and 82LR, both of which had greater than 10 samples identified, of which the first 10 were selected for testing so that these eplets were not over‐represented in our cohort.

When samples representing these potential Class I eplets were assessed with Explex, the eplet pattern was replicated in 49/62 samples (79%) (Table 7). Of interest, it was possible to predict the 80I pattern which distinguishes between the B*15:24/B62 and the B*38:01/B*38:02 in 5/5 cases, the 80K which divides the C*16:01/C*16:02 in 10/10 cases, and the 82LR which delineates the B*15:24/B62 in 10/10 cases. Unfortunately, the 138K which distinguishes the C*08:01/C*08:02 was only confirmed in 1/2 samples, likely because the C*05:01 is the only bead for this Eplet is on the standard panel. Explex testing did not replicate all cases of possible 21H, 73TVS, 80N and 177KT that were predicted based on the standard kit alone, although fewer samples were available to test for these eplets.

**TABLE 7:** Class I samples confirmed using Explex and prediction of bead reactivity at 2000 and 4000 MFI cut‐off.

Eplet	Eplet pattern replicated on Explex (%)	Prediction of Explex beads at 2000 MFI cutoff (%)	Prediction of Explex beads at 4000 MFI cutoff (%)
21H	0/2 (0)	0/1 (0)	0/1 (0)
73TVS	1/3 (33)	1/2 (50)	1/1 (100)
76ANT	5/9 (55)	4/8 (50)	2/5 (40)
76ESN	7/8 (87.5)	3/7 (43)	2/5 (40)
76VRN	5/6 (83.3)	2/5 (40)	2/5 (40)
80I	5/5 (100)	3/4 (75)	2/3 (67)
80K	10/10 (100)	7/9 (77)	5/7 (71)
80N	1/2 (50)	1/2 (50)	1/2 (50)
80TLR	1/1 (100)	1/2 (50)	NA
82LR	10/10 (100)	8/10 (80)	8/8 (100)
131S	3/3 (100)	1/3 (33)	1/2 (50)
138K	1/2 (50)	0/2 (0)	0/1 (0)
177KT	0/1 (0)	0/1 (0)	NA
Total	49/62 (79)	31/56 (55)	24/40 (60)

To confirm the difference in reactivity between B*55:01 and B*55:04 was due to the 131S, an AXE against a cell typed at a B*55:01 was performed (Figure S27). The post‐elution sample had a B*55:01 MFI of 2958 (elution efficiency of 31%), the B*55:02 MFI of 2765 (elution efficiency of 37%), and the B*55:04 MFI of 0 (elution efficiency of 0%). The 109 amino acid residue was identified to be crucial for this reactivity, as reactivity to all alleles that expressed both 131S and 109L were confirmed by AXE, whereas the B*35:02 (131S + 109F) did not elute.

We next investigated whether eplet patterns could be used to accurately predict Explex bead MFIs above or below set cutoffs (MFI 2000 and 4000, respectively), including both expected positive and negative beads in Table S9. Eplet patterns from the standard panel were included in this analysis if the majority of beads in that eplet were above these MFI respective cutoffs. At 2000 and 4000 MFI cutoffs, the positivity or negativity of beads on the Explex kit were accurately predicted in 31/56 cases (55%) and 24/40 cases (60%), respectively. Most inaccuracies occurred when an Explex bead that was anticipated to be negative was unexpectedly positive, likely due to the sera reacting to other additional eplets on that bead and accounting for the positive reaction. Occasionally, differences were due to beads that were anticipated to be positive but fell below the MFI cut‐offs. When focusing on the 82LR, 80K and 80I, which caused problems in the ‘closest bead strategy’, the standard kit alone accurately predicted positive and negative beads at the 2000 MFI cutoff and 4000 MFI cutoff in 18/23 and 15/18 cases, respectively.

Of note, there were substantially fewer samples showing reactivity to the 23 Class II eplets selected, with only 26 samples identified during the same period of testing. No samples were identified for the 11STS, 16Y, r37YV, 57S, r57V, 67LQ, 70QT, 74R, 69E or rp67IE eplets. When samples representing these potential Class II eplets were assessed with Explex, the eplet pattern was replicated in 25/26 (96%) samples (Table 8). However, when 2000 and 4000 MFI cutoffs were applied, the MFI of beads on the Class II Explex kit was accurately predicted to be above/below this cutoff in 13/22 cases (59%) and 8/15 cases (53%), respectively. The accuracy was not superior for the available eplets that delineated DR14 reactivity (70D, 70DA, 70R, rq70RK/R). There were too few examples of the q57V/125SQ and 56A/85GPM to establish their role in predicting DQ5 reactivity and DPB1*02:02 reactivity, respectively. In summary, using the Class II standard panel, it was possible to accurately predict whether this pattern would be replicated on Explex beads, although there was some difficulty in predicting whether the MFI of particular beads would be above or below various cutoffs.

**TABLE 8:** Class II samples confirmed using Explex and prediction of bead reactivity at 2000 and 4000 MFI cut‐off.

Eplet	Eplet pattern replicated on Explex (%)	Prediction of Explex beads at 2000 MFI cutoff (%)	Prediction of Explex beads at 4000 MFI cutoff (%)
70D	1/1 (100)	1/1 (100)	1/1 (100)
70DA	2/2 (100)	0/1 (0)	0/1 (0)
70R	1/1 (100)	1/1 (100)	0/1 (0)
q57V	2/2 (100)	2/2 (100)	1/1 (100)
125SQ	1/1 (100)	0/1 (0)	NA
35FV	0/1 (0)	0/1 (0)	0/1 (0)
56A	1/1 (100)	1/1 (100)	1/1 (100)
56E	1/1 (100)	NA	NA
56EE	1/1 (100)	NA	NA
84DEAV	7/7 (100)	2/6 (33)	1/2 (50)
85GPM	1/1 (100)	1/1 (100)	0/1 (0)
96K	1/1 (100)	1/1 (100)	1/1 (100)
rq70RK/R	6/6 (100)	4/6 (66)	3/5 (60)
Total	25/26 (96)	13/22 (59)	8/15 (53)

This group of samples was curated based on what appeared to be probable patterns of eplet reactivity on the standard panel. We attempted the same analysis on the highly‐sensitised renal transplant waiting list cohort. However, our initial utilisation of the matchmaker software to identify these eplets (Tables S9–S11) encountered problems in this cohort of highly sensitised individuals as multitude of potential eplets may be proposed by the matchmaker software when individuals are highly sensitised. To address this challenge, we adjusted our approach of identified potential eplets by defining an eplet based on both positive and negative reactivity (Tables S9–S11). All Class II eplets had negative beads represented on the standard panel (Tables S10 and S11). For the Class I eplets, the C*15:02 was utilised as the negative bead for 65QKR, the B*13:02 was utilised as the negative bead for 71TTS and 76ESN, the C*05:01 was the negative bead for 73TVS and 76VRN, the B*15:13 was the negative bead for 80N, and B*07:02 the negative bead for 131S.

With this approach, 22 out of 48 Class I samples were identified with an eplet that could divide serotypes. When an eplet was proposed from reviewing the standard panel, the expected positive beads were confirmed on the Explex kit in the majority of instances (27/32, 84%) (Table 9). These included the 138K, which contributed the most errors because it relies entirely on the C*05:01 as the sole positive bead on the standard panel. When excluding the 138K, if an eplet was predicted on the standard Class I panel, the expected positive beads were confirmed by Explex in 25/27 instances (93%). Unfortunately, in this cohort, the standard panel could not accurately predict negative beads in the Explex kit. Only 7/22 (32%) instances were correct, and this did not improve when excluding the 138K (5/17, 29%).

**TABLE 9:** Predicting positive and negative beads on Class I Explex using eplets proposed on standard panel.

Eplet	Correct positive beads Explex (%)	Correct negative beads on Explex (%)
73TVS	1/1 (100)	1/1 (100)
76ANT	5/5 (100)	1/5 (20)
76VRN	1/1 (100)	1/1 (100)
80I	5/6 (83)	1/6 (17)
80K	8/8 (100)	NA
80TLR	1/2 (50)	NA
82LR	2/2 (100)	1/2 (50)
131S	2/2 (100)	0/2 (0)
138K	2/5 (40)	2/5 (40)
Total	27/32 (84)	7/22 (32)

When evaluating Class II, 22 out of 50 samples were identified with an eplet that could divide serotypes. When an eplet was predicted based on the standard panel, the expected positive beads were present on Explex in the majority of instances (19/22, 86%) (Table 10). The two proposed eplets which contributed to the errors were the 16Y and the 125SQ. An eplet analysis was correct in predicting negative beads in 25/31 (81%) of instances.

**TABLE 10:** Predicting positive and negative beads on Class II Explex using eplets proposed on standard panel.

Eplet	Correct positive beads on Explex (%)	Correct negative beads on Explex (%)
16Y	0/2 (0)	0/2 (0)
r57V	NA	6/6 (100)
70D	1/1 (100)	1/1 (100)
70DA	1/1 (100)	1/1 (100)
70R	4/4 (100)	3/4 (75)
74R	NA	2/2 (100)
q57V	NA	1/1 (100)
125SQ	0/1 (0)	0/1 (100)
56E	4/4 (100)	4/4 (100)
56EE	1/1 (100)	1/1 (100)
84DEAV	2/2 (100)	2/2 (100)
96K	2/2 (100)	1/2 (50)
rq70RK/R	4/4 (100)	3/4 (75)
Total	19/22 (86)	25/31 (81)

For this highly sensitised cohort, it was not possible to predict the 138K, 16Y or 125SQ eplets based on the standard panel. If these eplets which are ‘difficult to predict’ were excluded, an eplet predicted from the standard panel was replicated on the corresponding Explex beads in the vast majority of cases. However, negative bead reactivity on Explex can only be predicted with modest accuracy for Class II, and cannot be predicted accurately for Class I. Predicting which Explex beads will be negative was likely more challenging in this cohort due to other reactivity patterns leading to unanticipated positive results on the Explex kit in this highly sensitised population.

## 4 Discussion

This evaluation of the Explex kit involved a comparison to the closest related bead in the standard kit, and a comparison to an eplet pattern predicted by the standard kit beads. In evaluating the closest bead strategy, the majority of Explex beads correlated well with their closest related beads in the standard kit. The most common difference at the A‐locus was when an antibody bound the A*02:18 but not A*02:01, after patients were exposed to HLA‐C alleles that share the same K138 residue as the A*02:18. These patients also had differing reactivity between the C*08:01 and C*08:02/C*08:04. At the B‐locus, we identified a difference in the B*38:01 and B*38:02 (Bw4 80I and Bw4 80T groups, respectively), as well as the B*15:01 and B*15:24 (B62 Bw6 and B62 Bw4 80I, respectively). At DRB1, differences were noted with DRB1*14:03, DRB1*14:04 and DRB1*14:05. A difference was also noted between the DQB1*05:03 and DQB1*05:01. Differences were noted in the C*16:01 and C*16:02 that belong to the HLA‐C1 and HLA‐C2 groups, respectively.

Although the above differences were discovered, several of these may be overcome by analysing possible eplet patterns on the standard kit. In a cohort of specimens curated based on their likely eplet reactivity on the standard kit, the eplet pattern of reactivity was replicated on Explex beads in 78% of cases for Class I and 96% of cases for Class II. Furthermore, the eplet patterns such as the 80I, 80K, 82LR, rq70RK/R that resulted in differences in nearest bead strategy could reliably predict reactivity to B*38:02, B*15:24, C*16:01 and DR14 in the majority of cases. Although it may be possible to predict whether an eplet is present using the standard panel alone, it was more challenging to predict whether Explex beads were above or below MFI cutoffs. In most cases this was because other antibody reactivities may also contribute to reactivity leading to stronger than anticipated reactivity or the MFI may be weaker than the cutoff. Although single antigen bead assays are intended to be qualitative in nature, MFI cutoffs are often used in laboratories to determine unacceptable alleles.

When eplet analysis of standard kits was performed on a separate cohort of highly sensitised patients, 64% of potential Class I eplets were replicated on Explex, and 86% of Class II eplets were replicated on Explex. However, prediction of Explex beads that were anticipated to be negative beads for Class I was not accurate for this cohort. Our current tools in predicting responsible eplets in complex sera have limitations, and occasionally alleles that are anticipated to be negative will be positive due to alternative eplets. In these circumstances, additional testing of these alleles either by Explex or supplementary kits can be helpful. Although this additional testing can improve the accuracy of the virtual crossmatch, one potential drawback is the increase in unacceptable antigens and matched panel reactive antibody for these individuals, in addition to financial and workload implications for laboratories.

Inaccuracy in correctly predicting eplets may be related to the number of beads representing the specific eplet in the standard kit, with greater difficulty predicting eplets when there are too few or too many beads. Insufficient beads (such as in the case of the 138K) can make it difficult to predict an eplet, and too many beads could lead to the possibility of multiple eplets being responsible for the same pattern of antibody reactivity. In addition, the accuracy of eplet predictions was lower in the highly sensitised populations due to other potential different eplets leading to similar reactivity patterns.

There are several strengths to our current study. As far as we are aware, this is the first evaluation of additional recombinant HLA beads in an Australian population. The Australian population has a large migrant population with many ethnic diversities, making it a valuable population to study and is reasonably comparable to other large jurisdictions with populations of mixed ethnicity. Furthermore, our study involved a comprehensive bead by bead and eplet by eplet investigation.

All studies of single antigen bead technologies must address limitations related to this technology. When evaluating the antigen density between the kits, significant differences in the antigen density of two beads could lead to differing results. This contributes to a systemic error or bias, and R/R^2^ statistics are not typically affected. W6/32 and FJ normalisation tools can help address this problem. One limitation of our study is that we did not perform these normalisations. Instead, to overcome the risk of this affecting our data on accuracy, we utilised stringent criteria to separate positive and negative results (with any result with an MFI 1000‐4000 considered indeterminate). To reduce the effect of interassay variability we also excluded samples that had greater than log2 differences in NC/PC values. Finally, non‐specific reactivity has been noted with single antigen bead assays due to denatured HLA molecules, natural antibodies, or tags used to in the manufacturing of recombinant HLA and this may lead to spurious reactivity in either the standard or Explex kits. This may explain some variability between C*01:02 and C*01:03, C*14:03 and C*14:02 which could not be explained by eplet mismatches or known immunisation.

Our results cohere with initial data from Osoegawa et al. [8], which identified distinct serological reactivity for A*02:05/A*02:01, A*02:01/A*02:18, B*15:01/B*15:24, C*08:01/C*08:02/C*08:04, C*16:01/C*16:02 in a similar number of patients undergoing testing with supplemental kits. Our correlation analysis identified some individuals with 2–4 fold differences in MFI for the B*39:01 and B*39:02. This coheres with the fact that the B3901 and B3902 are WHO recognised associated antigens, with differences in critical residual positions 63 and 67 [13]. No categorical differences between B3901 and B3902 were noted, in our cohort. However, this may be due to the fact that no patients in our cohort were typed as a B*39:02 or sensitised to a B*39:02. We also identified a difference in the serological specificity between the B*39:04 and the rest of the B39 antigen group in one individual due to a non‐verified (12M) eplet. The 12M is characterised by an alanine at residue 11 (as opposed to a serine) and a methionine at residue 12 (as opposed to a valine). Although these residues are considered inaccessible to solvent, differing serological reactivity has also been described by Ogawa et al. and presumed to be due to conformational changes in more accessible epitopes [14].

One difference between our data and that from Osoegawa et al. is that we identified a difference between B*38:01 and B*38:02 based on the 80I/80T residues, whereas Osoegawa et al. predict the B*38:02 to be a ‘FULL’ member of the B‐3801 group. No categorical differences (positive vs. negative) were noted in our cohort for the B*39:01 and B*39:06. However, differences in MFI strength between the B*39:01 (MFI 11703) and B*39:06 (MFI 2756, a ‘FULL’ member of the B3901 group) were noted in sera that bound to the 158T (antibody‐verified Eplet). The B*39:01 and B*39:06 differ at the 95 and 97 amino acid residues. Our data also showed absorption/elution evidence for the 177DT, a hitherto unverified eplet. Allosera that bound to this eplet bound to both the B*41:02 and B*41:01, but the B*41:01 was weaker in MFI, suggesting less antibody avidity for the B*41:01 allele. The alleles again differ at the 95 and 97 amino acid residues (leucine vs. serine, and tryptophan vs. arginine), which are distant from the 177DT. However, the 95 and 97 amino acid residues constitute parts of the C, E and F pockets of the peptide‐binding groove [15], and it is possible that the avidity of antibodies that target the 177DT and 158T eplets may be influenced by differences in peptide repertoire.

We also identified a case of a patient who was DQB1*05:03 who developed antibodies to DQB1*05:01 (a ‘FULL’ member of the DQ5 group). Although novel, these specific differences were confirmed in our cohort by absorption/elution studies. We also noted a difference between the DPB1*13:01 and DPB1*30:01, which has been attributed to differences in exon 3, (residue 96 lysine vs. arginine) in other cases [16]. We investigated this possibility, but the antibody reactivity could not be attributed to this residue. Instead, the reactivity was confirmed by an AXE that demonstrated the 55EAE eplet pattern that includes the DPB1*30:01, but not the DPB1*13:01.

We were able to confirm differing reactivity to the C*04:03 and C*04:01 using cell‐based assays. Of interest, the elution against the C*04:03 cell eluted antibodies to 8/9 alleles that express the 21H eplet; the MFIs for the elute of these were strong (> 3000 MFI) but could not explain all the C‐locus reactivity seen. The eluate also bound to 23/26 beads that represent the 11AV non‐verified eplet, that had a broader range of MFI (500–5900). Elute did not bind to the C*14:03, C*07:04, C*07:02 or the B*14:02 which differ at 11, 95, 99 and 66–71 amino acid residues from the remainder of the alleles that express either the 11AV and 21H. Given the proximity of these relevant amino acids, it is possible that this represents a conformational peptide‐dependent epitope, that has yet to be mapped. Therefore, while we can confirm a difference between C*04:03 and C*04:01, we have not definitively proven that it is related to either 21H or 11AV. Recently, structural dimorphism at the epitope 103L + 109L versus 103V + 109L was found by AXE to distinguish between the B*55:01 and B*55:04 [17]. This was investigated but did not explain the pattern of reactivity in our patient, as the B*48:01 and C‐locus alleles that are 103L + 109L were negative. Instead, AXE in our patient confirmed reactivity to alleles that expressed both 131S and 109L, which excludes the B*35:02 in the Explex panel. Of note, the original paper that identified the 131S eplet did not assess the reactivity against B*35:02 [18]. Based on our findings and the existing literature, we conclude that more than one pattern of antibody reactivity and structural epitopes distinguish between the B*55:01 and B*55:04.

The limitations of our study include the fact that this is a single centre study, rather than sampling various international populations. Second, we performed a very targeted analysis. For example, for the closest bead strategy, we chose to analyse a highly sensitised population of patients on the renal transplant waiting list (PRA > 95%). The reason for choosing this population was because they are more likely to have anti‐HLA antibodies and therefore would be a higher yield population when attempting to correlate between the Explex and standard kits. It could be argued that the correlation between different beads could be artificially elevated by chance because the cohort is highly sensitised. However, an examination of completely unrelated beads, such as the DPB1*02:01 and DPB1*02:02 showed a Pearson correlation *R* of −0.01 and *R* ^2^ of 0 (Table 6), indicating that the study design identified scenarios when no association was present. For further comprehensive evaluation, it would be informative to repeat this exercise in a population with a lower degree of sensitisation (70%–95%) to determine if the correlations differed in this population, and lower degrees of sensitisation especially to identify patterns of non‐specific reactivity that might occur on Explex kits as seen elsewhere [12].

Our eplet analysis was confined to eplets which are predicted to lead to differences between alleles that share the same serotype. This is because the ‘closest related bead’ strategy should already accurately predict reactivity within the eplets that define a serotype. Although this is the most suitable group of eplets to analyse, the results of our eplet analysis have not been generalised to all eplets or non‐verified eplets. Finally, our Class II eplet analysis was limited to 26 samples, and it would be important to confirm the findings in a larger cohort.

Finally, the study's conclusions are primarily drawn from statistical correlation of MFI values between standard and Explex beads. However, one limitation of this study design is that absorption‐elution experiments (AXE) were not performed on all samples to confirm all reactivity patterns. Unexplained variability such as to the C*01:02/C*01:03, that could not be explained by either sensitisation history nor known antibody‐binding patterns were presumed, but not proven, to be related to non‐specific binding. Instead, we have relied on prior published literature which detail serotypes and antibody‐verified eplets. Nonetheless, one strength of our study is that some of the more critical observations such as the antibody reactivity to the 80I, q57V, 138K eplets were confirmed by AXE.

This study should prompt other laboratories to consider whether these findings are consistent in their own local populations, as it is possible that different ethnic groups may benefit from different alleles in the Explex or supplementary kits. Comparing local allele frequencies with the alleles in Tables S9–S11 may be helpful to assess potential utility of these additional beads. Many Explex bead specificities are not frequent in all populations. The most common of these alleles worldwide would be the DQB1*05:03 (2.6% of Caucasoids and 8.8% of API [3]). However, only one individual had differing reactivity for the DQB1*05:03 and DQB1*05:01, potentially because the antibody‐verified eplet with single amino acid difference may not be frequently immunogenic. Other frequent alleles include the B*38:02 (1.8% of API), DRB1*14:03 (0.1% of API), the DRB1*14:04 (6.3% of API), and DRB1*14:05 (0.4% of API), but which are only present in < 0.01% of the European Caucasoid population [3]. The C*08:02 (2.5% of Caucasoids) and to a lesser degree the C*16:02 (0.4% of Caucasoids and 1.9% of API) are commonly seen in various populations worldwide [3]. Ideally, standard Luminex standard kits should be modified to include some of these helpful beads and remove ‘unnecessary’ beads. Indeed, this work and that of Osoegawa et al. [8], provides the groundwork for further rationalising of standard panels by removing beads that have excellent correlation with other beads in the standard panel.

With regards to the application of our findings, it is important to recognise the limitations of our current technologies in virtual crossmatch. Our study shows that in many cases, bead reactivity for unrepresented alleles can be predicted based on the standard panel, either by using a closest related bead or by looking at possible eplet patterns. The data in this study also indicate scenarios where this estimation may be inaccurate: for example, particular eplets which split antigen groups and have too few or too many beads in the standard kit. Another example is highly sensitised populations, as the accuracy of predicting eplet patterns may be reduced in the context of multiple other antibody specificities. In our population, this data indicates that Explex kits are useful in the circumstance when additional alleles are not accurately predicted by the closest related allele on the standard panel, and when eplet reactivity is difficult to accurately characterise due to complex sera.

## Author Contributions

G.J.C. was responsible for the conception, design, analysis, interpretation and drafting the work. R.L.C., D.Q., F.G., was responsible for acquisition and analysis. P.G., A.T., S.K. and L.G. were responsible for analysis, interpretation. R.P.C., L.C.S. were responsible for analysis, interpretation and supervision. All above authors were responsible for critical review of the work, final approval of the published version and agree to be accountable for all aspects of the publication.

## Funding

Support for this research was provided in‐kind by One Lambda in the form of diagnostic kits. No additional external funding was received.

## Conflicts of Interest

Explex kits were provided by One Lambda. One Lambda was not involved in the design of this study nor composition of this publication. The other authors declare no conflicts of interest.

## Supporting information


**Figure S1:** Correlation and amino acid alignment for A*02:01/A*02:05, A*02:01/A*02:10, A*02:01/A*02:18, A*26:01/A*26:03.

**Figure S2:** Correlation and amino acid alignment for B*15:01/B*15:24, B*38:01/B*38:02, B*39:01/B*39:02, B*39:01/B*39:02.

**Figure S3:** Correlation and amino acid alignment for B*39:01/B*39:06, B*39:01/B*39:13, B*50:01/B*40:05, B*41:01/B*41:02.

**Figure S4:** Correlation and amino acid alignment for B*15:03/B*48:02, B*55:01/B*55:04, B*56:01/B*56:03, B*15:01/B*15:20.

**Figure S5:** Correlation and amino acid alignment for DRB1*14:02/B*14:03, DRB1*14:01/DRB1*14:04, DRB1*14:01/DRB1*14:05.

**Figure S6:** Correlation and amino acid alignment for DQB1*05:01/DQA1*01:01 and DQB1*05:03/DQA1*01:01.

**Figure S7:** Correlation and amino acid alignment for C*01:02/C*01:03, C*04:01/C*04:03, C*07:02/C*07:01, C*07:02/C*07:04.

**Figure S8:** Correlation and amino acid alignment for C*08:01/C*08:02, C*08:01/C*08:04, C*14:02/C*14:03, C*16:01/C*16:02.

**Figure S9:** Correlation and amino acid alignment for Explex/Standard panel DP alleles.

**Figure S10:** Luminex profiles with reactivity to alleles sharing the 138K eplet.

**Figure S11:** Luminex profiles with varying reactivity to alleles within the A2 antigen group, and the A26 antigen group.

**Figure S12:** Luminex profiles with varying reactivity to alleles within the B62 antigen group, and the B39 antigen group.

**Figure S13:** Luminex profiles with reactivity to alleles sharing the 12M eplet, and 63NI eplet.

**Figure S14:** Luminex profiles with reactivity to alleles sharing the 103L eplet, and the 11STS eplet.

**Figure S15:** Luminex profiles with reactivity to alleles sharing the 80K eplet.

**Figure S16:** Luminex profile with varying reactivity to alleles within the Cw7 antigen group, and Luminex profile with reactivity to alleles sharing the 55EAE eplet.

**Figure S17:** Luminex profile with reactivity to alleles sharing the 12M eplet.

**Figure S18:** AXE experiment confirming the reactivity to alleles sharing the 80I eplet.

**Figure S19:** AXE experiment confirming the reactivity to alleles sharing the 138K eplet.

**Figure S20:** AXE experiment confirming the reactivity to alleles sharing the 158T eplet.

**Figure S21:** AXE experiment confirming the reactivity to alleles sharing the 158T eplet.

**Figure S22:** AXE experiment confirming the reactivity to alleles sharing the 177DT eplet.

**Figure S23:** AXE experiment confirming the reactivity to alleles sharing the 177DT eplet.

**Figure S24:** MFI of serum and AXE experiments eluates targeting the 177DT.

**Figure S25:** AXE experiment confirming the reactivity to alleles sharing the q57V eplet.

**Figure S26:** AXE experiment confirming the different reactivity to alleles within the Cw4 antigen group.

**Figure S27:** AXE experiment confirming the reactivity to alleles sharing the combination of 131S/109L eplets.

**Figure S28:** AXE experiment confirming the reactivity to alleles sharing the 55EAE eplet.

**Table S1:** Correlation between A locus Explex beads and their closest related bead on the standard panel. POS_POS is when both beads are > 4000, POS_NEG is when the MFI for standard bead > 4000 and MFI for Explex bead is < 1000, NEG_POS is when the MFI for standard bead is < 1000 and MFI for Explex bead is > 4000, NEG_NEG is when MFI for both SABS are < 1000. Questionable is when the MFI for one or both beads is between 1000 and 4000.
**Table S2:** Correlation between B locus Explex beads and their closest related bead on the standard panel. POS_POS is when both beads are > 4000, POS_NEG is when the MFI for standard bead > 4000 and MFI for Explex bead is < 1000, NEG_POS is when the MFI for standard bead is < 1000 and MFI for Explex bead is > 4000, NEG_NEG is when MFI for both SABS are < 1000. Questionable is when the MFI for one or both beads is between 1000 and 4000.
**Table S3:** Correlation between DR locus Explex beads and their closest related bead on the standard panel. POS_POS is when both beads are > 4000, POS_NEG is when the MFI for standard bead > 4000 and MFI for Explex bead is < 1000, NEG_POS is when the MFI for standard bead is < 1000 and MFI for Explex bead is > 4000, NEG_NEG is when MFI for both SABS are < 1000. Questionable is when the MFI for one or both beads is between 1000 and 4000.
**Table S4:** Correlation between DQ locus Explex beads and their closest related bead on the standard panel. POS_POS is when both beads are > 4000, POS_NEG is when the MFI for standard bead > 4000 and MFI for Explex bead is < 1000, NEG_POS is when the MFI for standard bead is < 1000 and MFI for Explex bead is > 4000, NEG_NEG is when MFI for both SABS are < 1000. Questionable is when the MFI for one or both beads is between 1000 and 4000.
**Table S5:** Correlation between C locus Explex beads and their closest related bead on the standard panel. POS_POS is when both beads are > 4000, POS_NEG is when the MFI for standard bead > 4000 and MFI for Explex bead is < 1000, NEG_POS is when the MFI for standard bead is < 1000 and MFI for Explex bead is > 4000, NEG_NEG is when MFI for both SABS are < 1000. Questionable is when the MFI for one or both beads is between 1000 and 4000.
**Table S6:** Correlation between DP locus Explex beads and their closest related bead on the standard panel. POS_POS is when both beads are > 4000, POS_NEG is when the MFI for standard bead > 4000 and MFI for Explex bead is < 1000, NEG_POS is when the MFI for standard bead is < 1000 and MFI for Explex bead is > 4000, NEG_NEG is when MFI for both SABS are < 1000. Questionable is when the MFI for one or both beads is between 1000 and 4000.
**Table S7:** Demographics and known plausible sensitisation history of highly sensitised cohort.
**Table S9:** Class I eplets predicted to split antigen groups or serotypes.
**Table S8:** Age, sex and sensitisation history of highly sensitised renal patient cohort.
**Table S10:** Class II eplets predicted to split antigen groups or serotypes.
**Table S11:** DP eplets predicted to react with DP beads on the Explex panel. *DPB1*105:01 and DPB1*04:02 are in the same P‐group.

## Data Availability

The data that support the findings of this study are available on request from the corresponding author. The data are not publicly available due to privacy or ethical restrictions.
