# Supplementary figures and images for: Standard Single Antigen HLA Luminex Panels Predict Most, but Not All, Antibody Reactivity Against Alleles in Extended Panels
Source: HLA. 2026 Jul 15;108(1):e70797. doi: 10.1111/tan.70797 (PMC13370519; doi:10.1111/tan.70797)

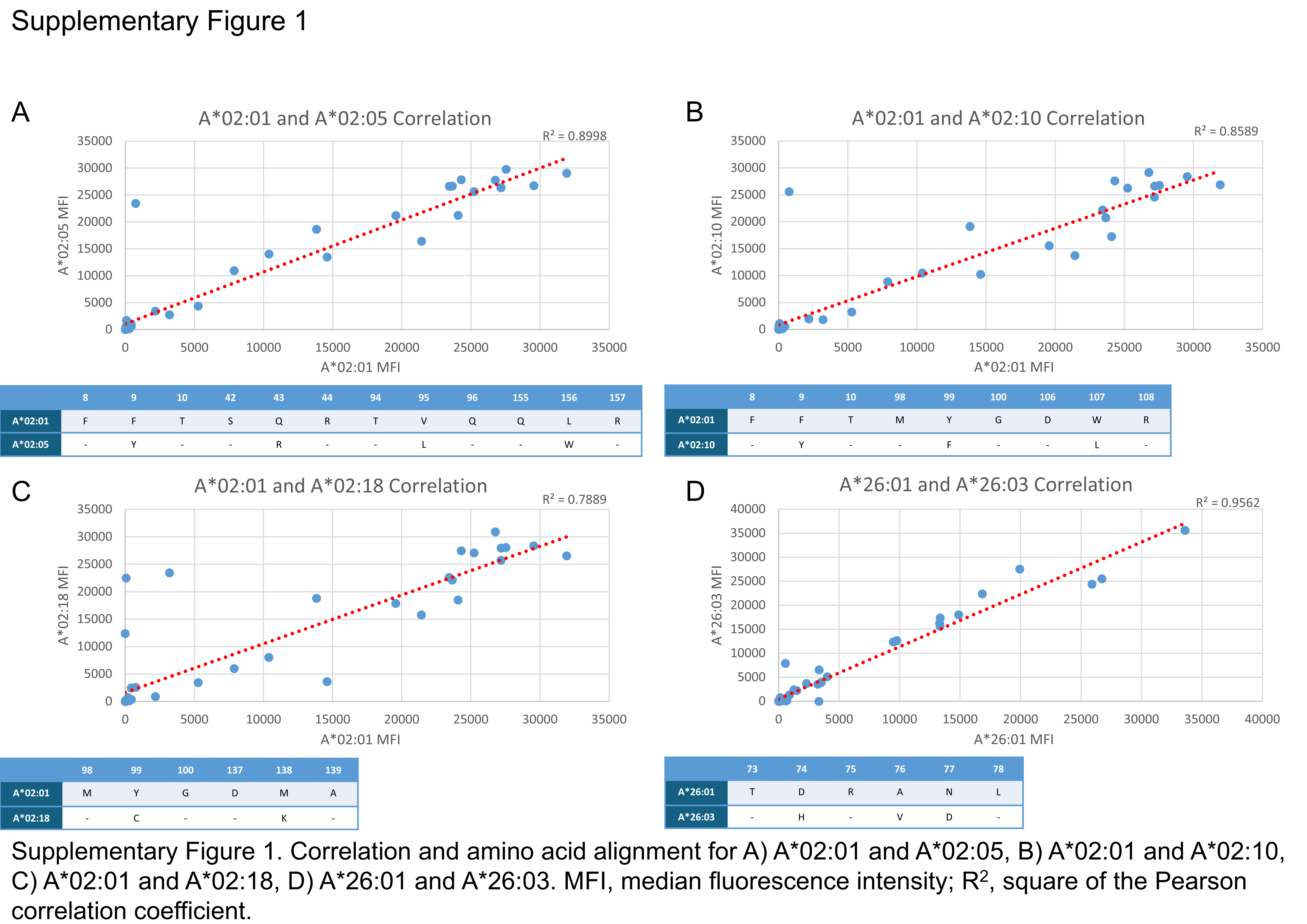

Supplement: Supplementary file 1 — Figure S1: Correlation and amino acid alignment for A*02:01/A*02:05, A*02:01/A*02:10, A*02:01/A*02:18, A*26:01/A*26:03. [file TAN-108-e70797-s002.tif]

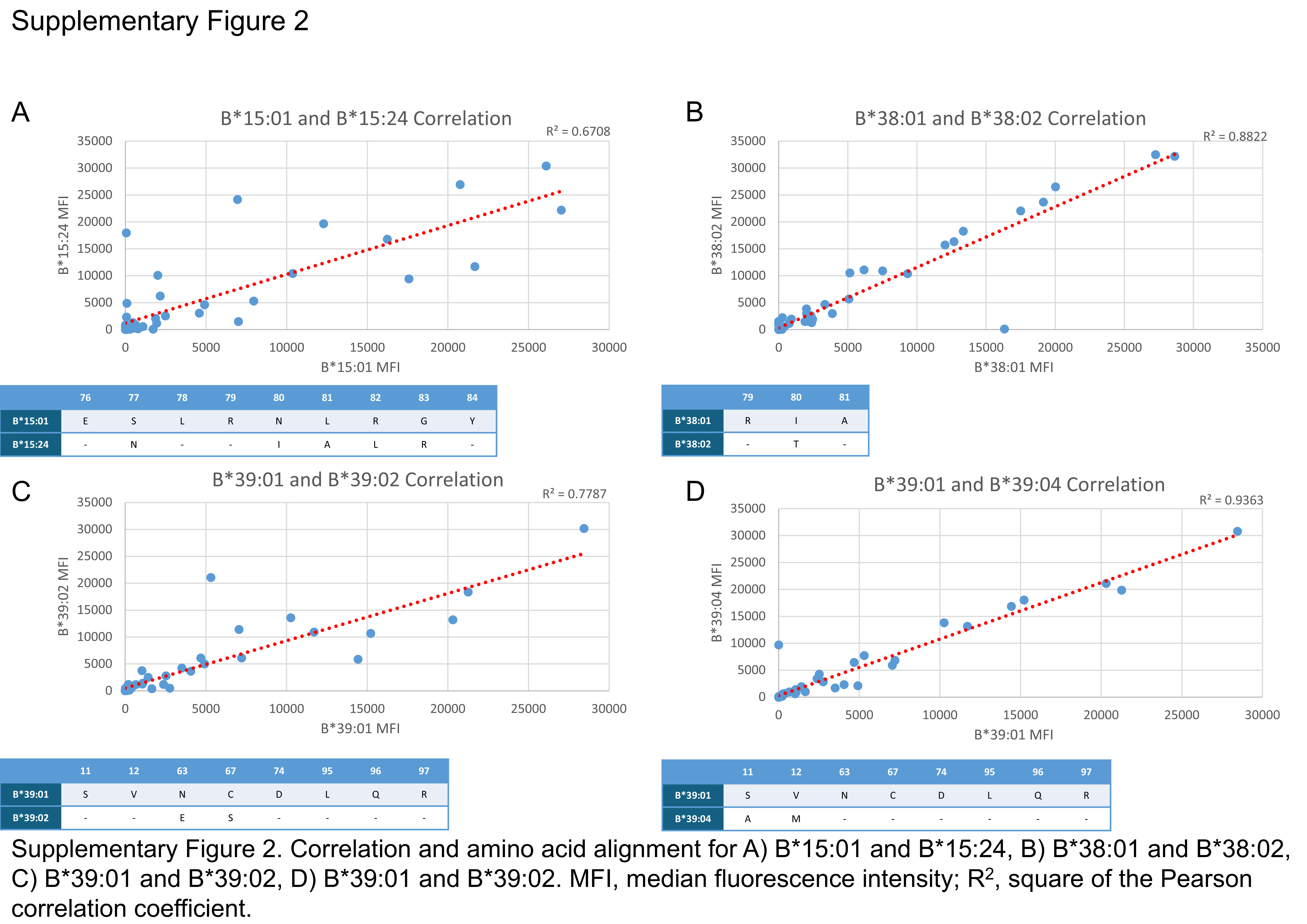

Supplement: Supplementary file 2 — Figure S2: Correlation and amino acid alignment for B*15:01/B*15:24, B*38:01/B*38:02, B*39:01/B*39:02, B*39:01/B*39:02. [file TAN-108-e70797-s007.tif]

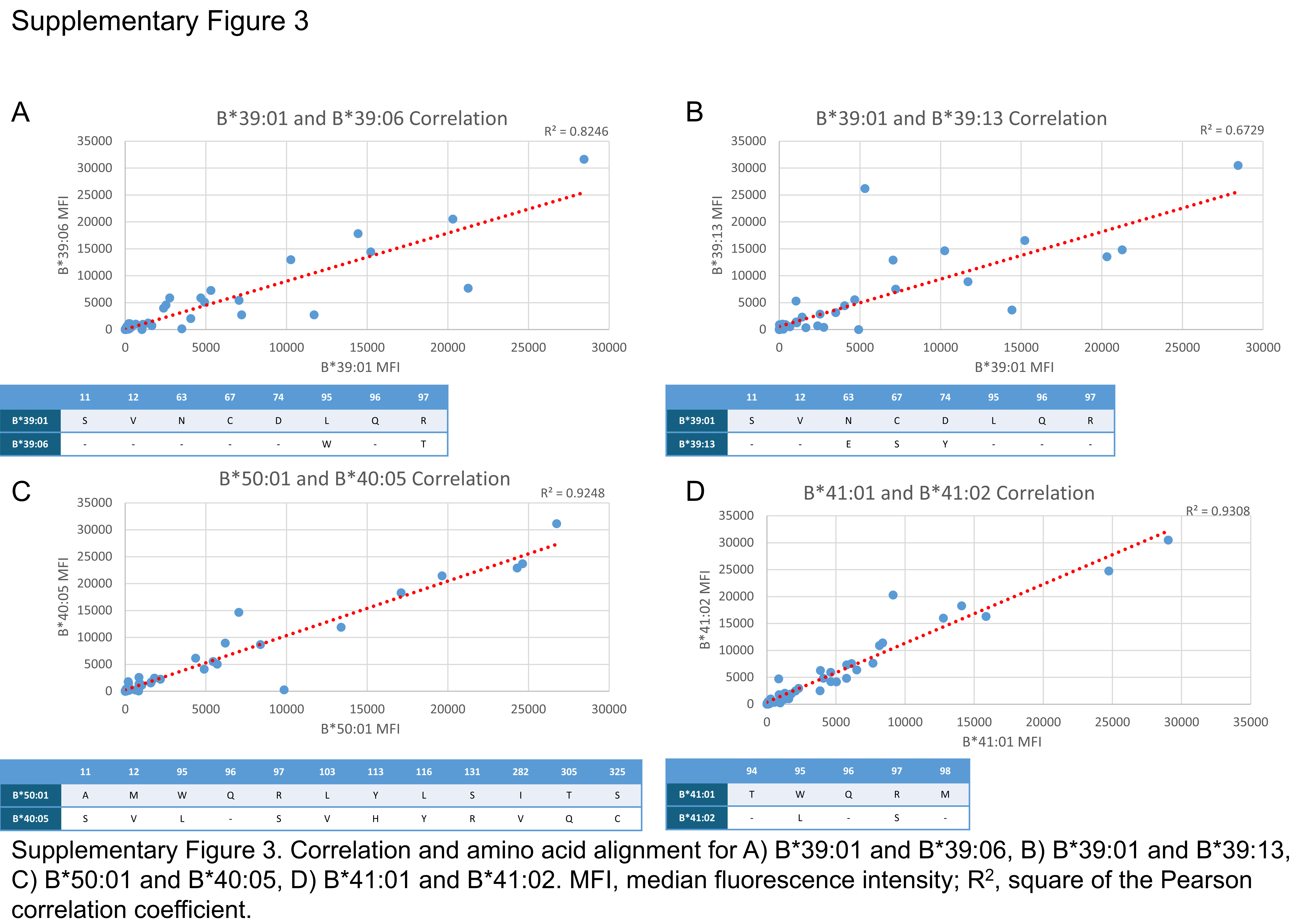

Supplement: Supplementary file 3 — Figure S3: Correlation and amino acid alignment for B*39:01/B*39:06, B*39:01/B*39:13, B*50:01/B*40:05, B*41:01/B*41:02. [file TAN-108-e70797-s015.tif]

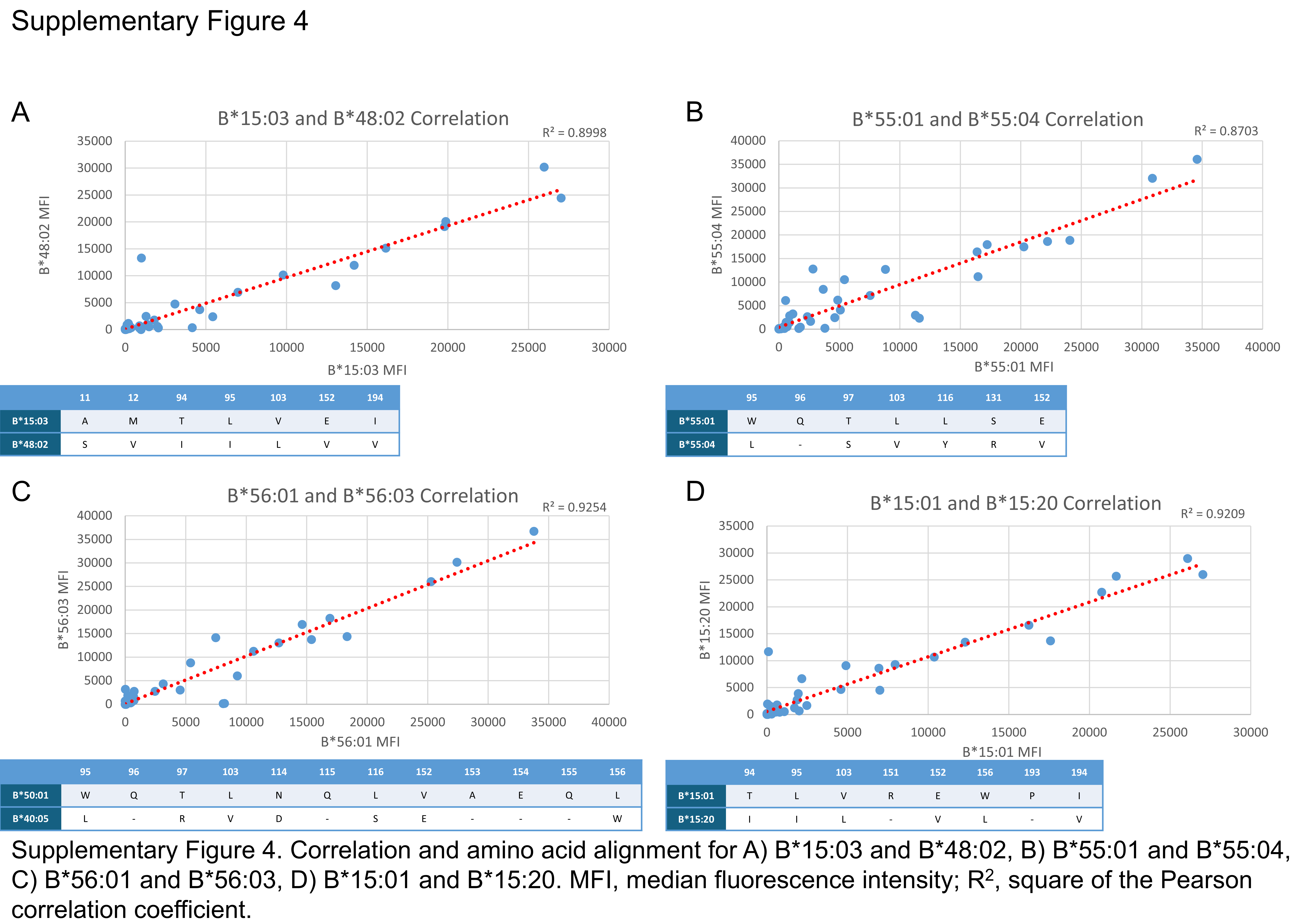

Supplement: Supplementary file 4 — Figure S4: Correlation and amino acid alignment for B*15:03/B*48:02, B*55:01/B*55:04, B*56:01/B*56:03, B*15:01/B*15:20. [file TAN-108-e70797-s009.tif]

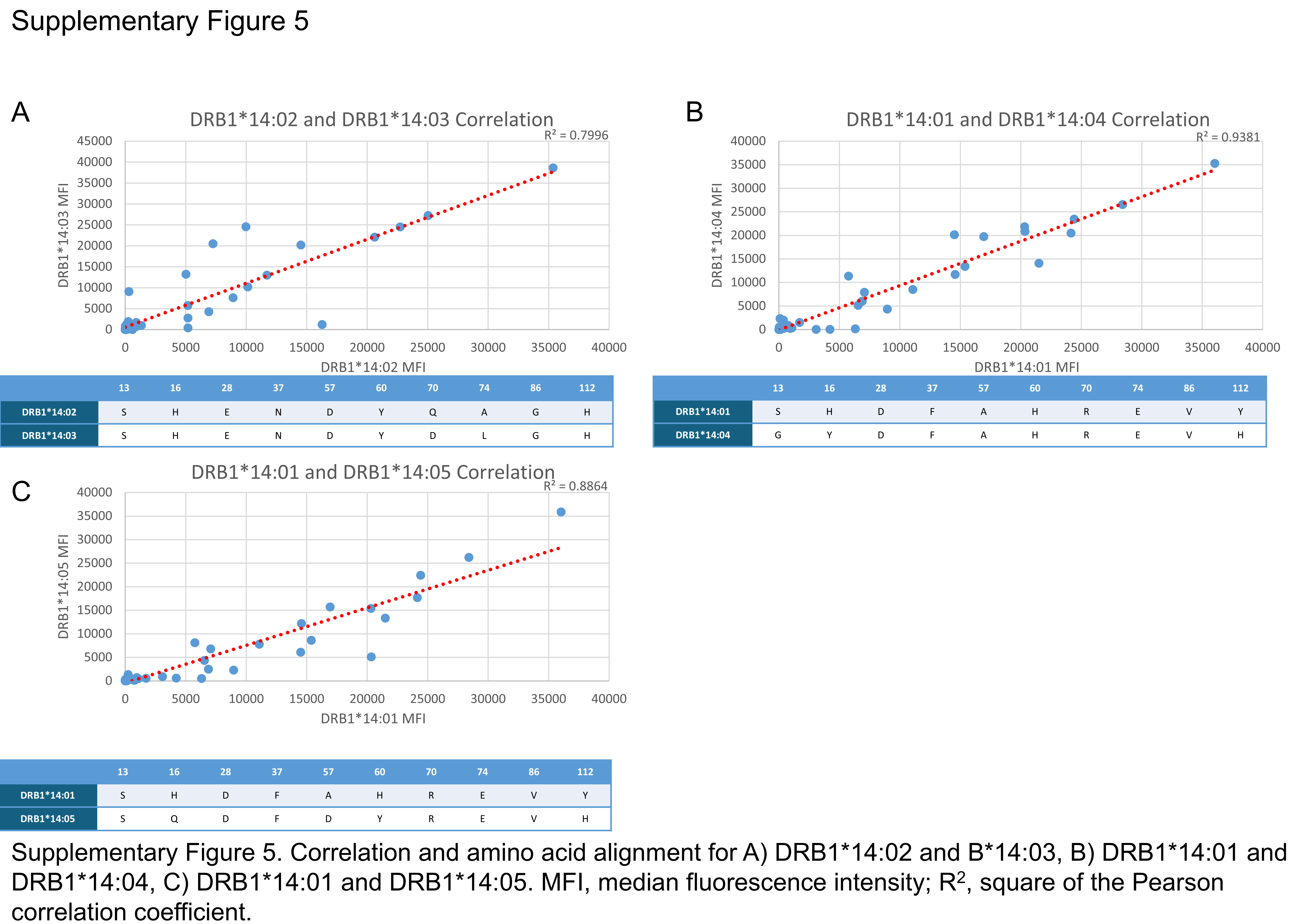

Supplement: Supplementary file 5 — Figure S5: Correlation and amino acid alignment for DRB1*14:02/B*14:03, DRB1*14:01/DRB1*14:04, DRB1*14:01/DRB1*14:05. [file TAN-108-e70797-s028.tif]

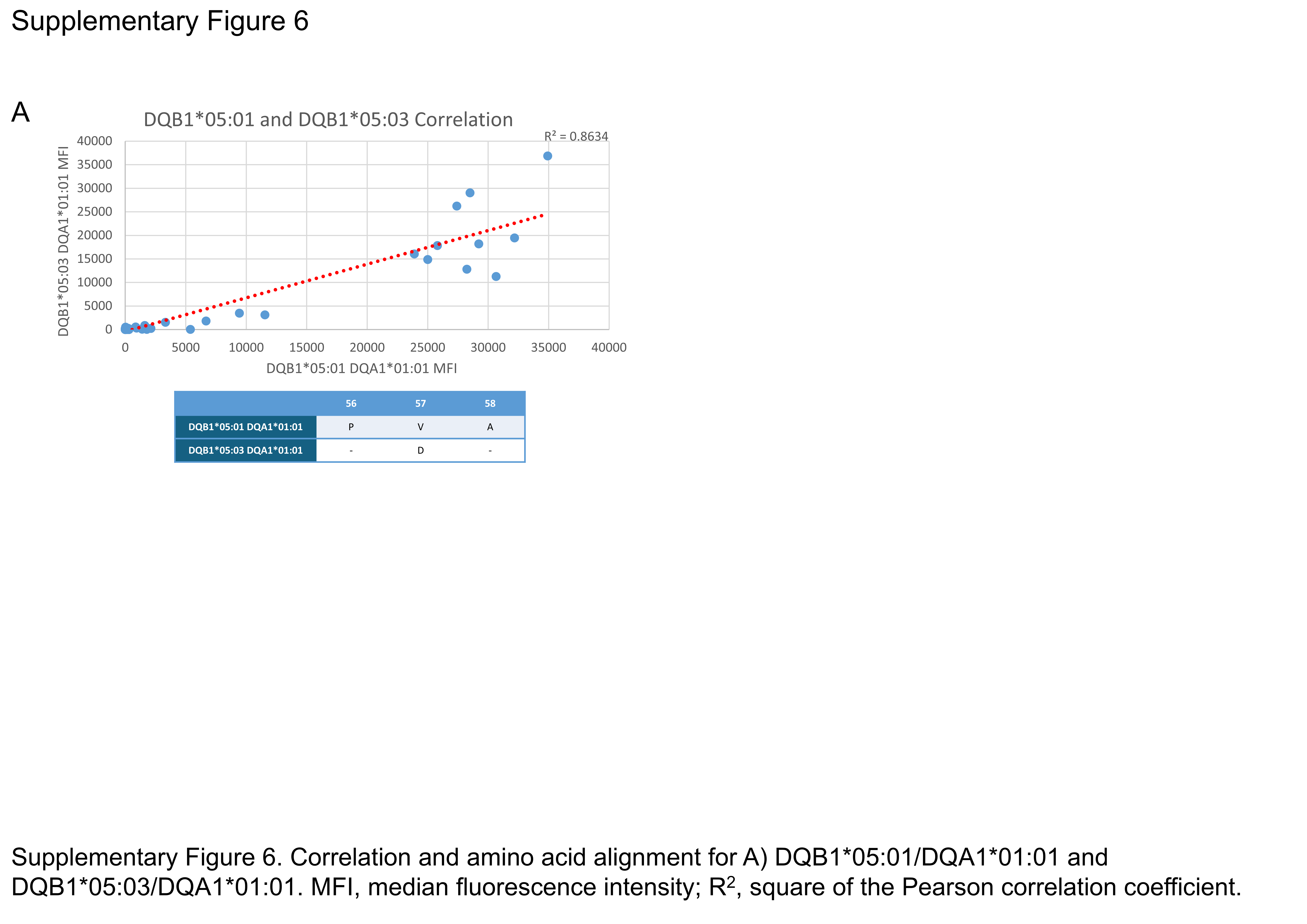

Supplement: Supplementary file 6 — Figure S6: Correlation and amino acid alignment for DQB1*05:01/DQA1*01:01 and DQB1*05:03/DQA1*01:01. [file TAN-108-e70797-s025.tif]

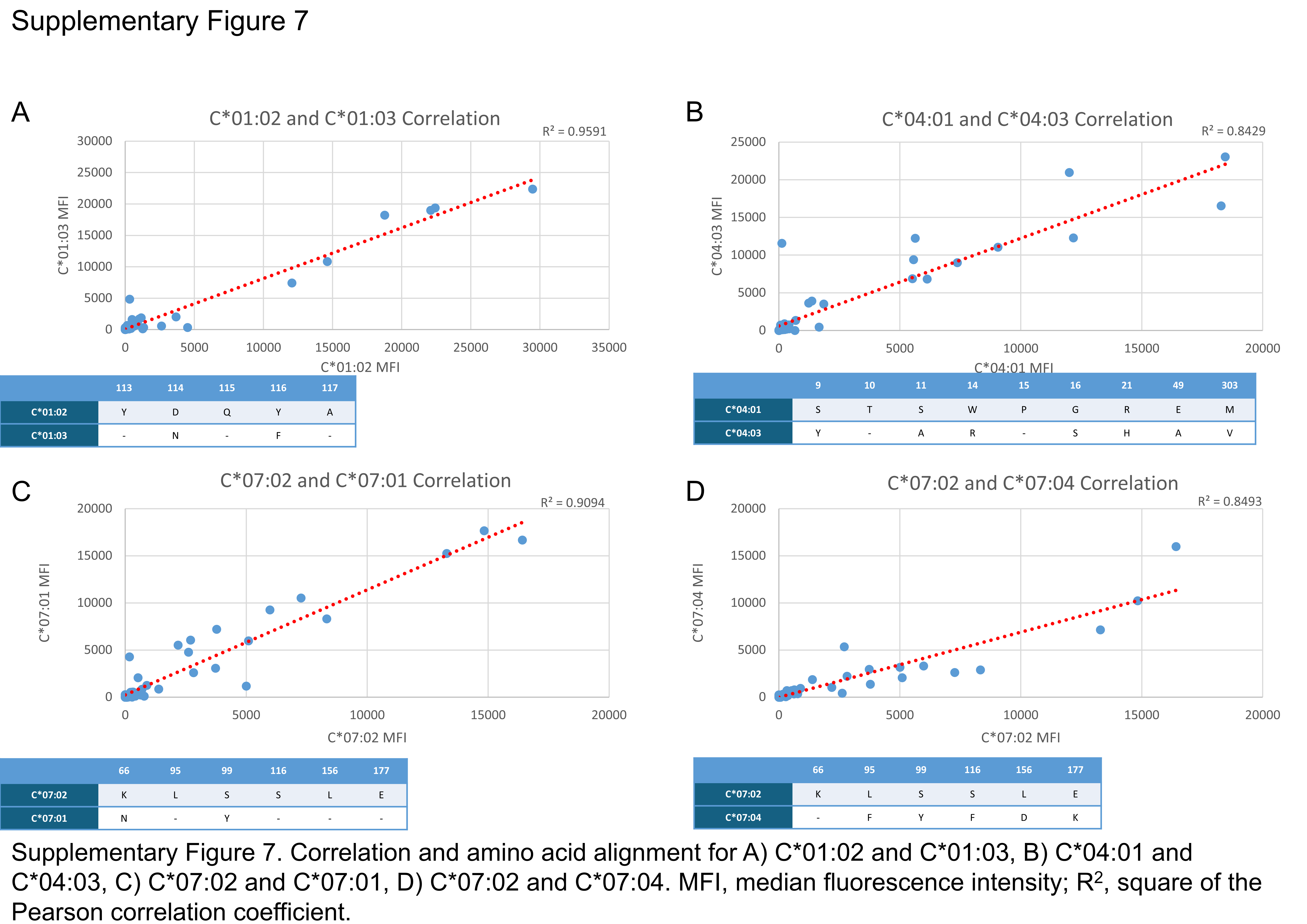

Supplement: Supplementary file 7 — Figure S7: Correlation and amino acid alignment for C*01:02/C*01:03, C*04:01/C*04:03, C*07:02/C*07:01, C*07:02/C*07:04. [file TAN-108-e70797-s014.tif]

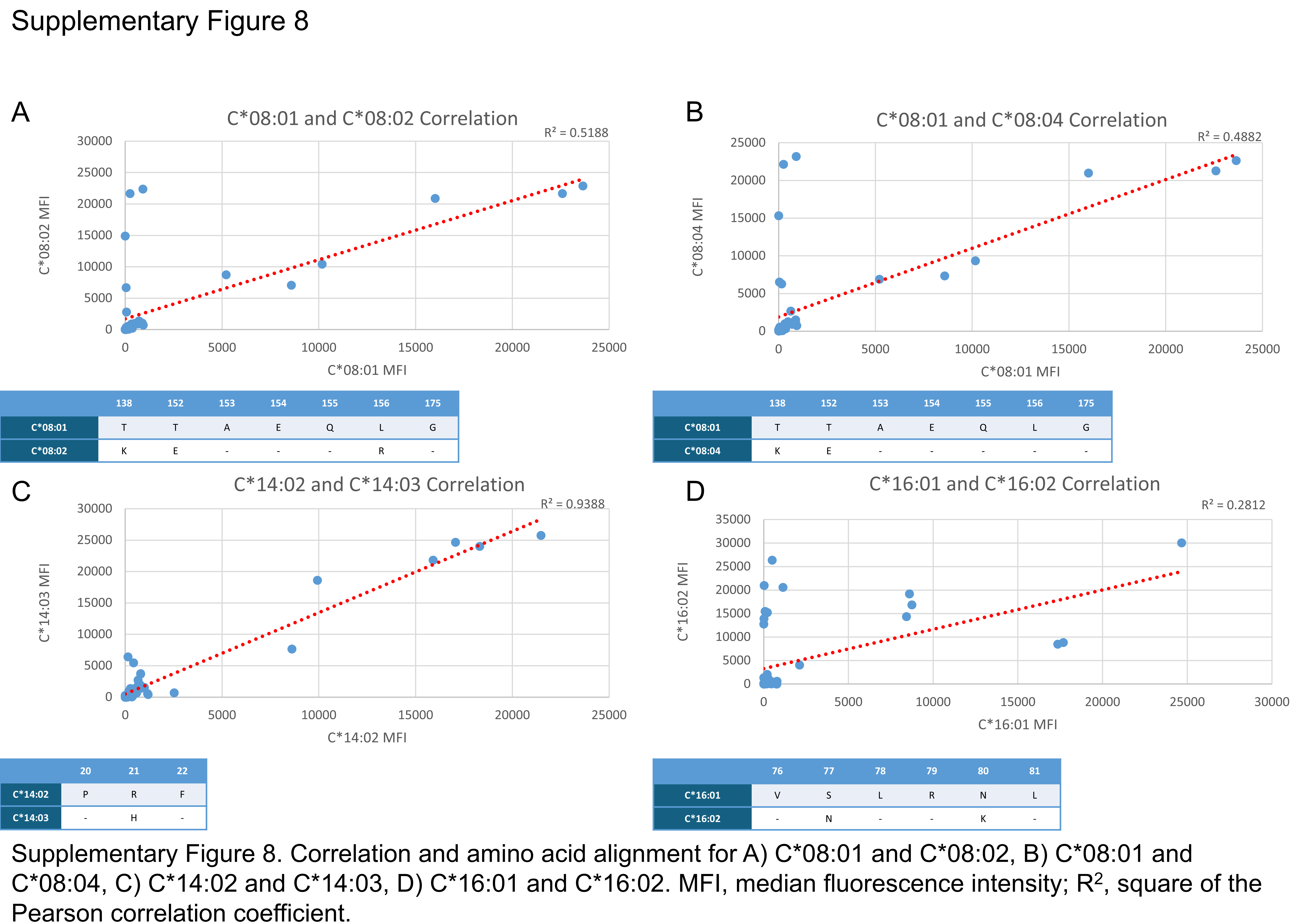

Supplement: Supplementary file 8 — Figure S8: Correlation and amino acid alignment for C*08:01/C*08:02, C*08:01/C*08:04, C*14:02/C*14:03, C*16:01/C*16:02. [file TAN-108-e70797-s018.tif]

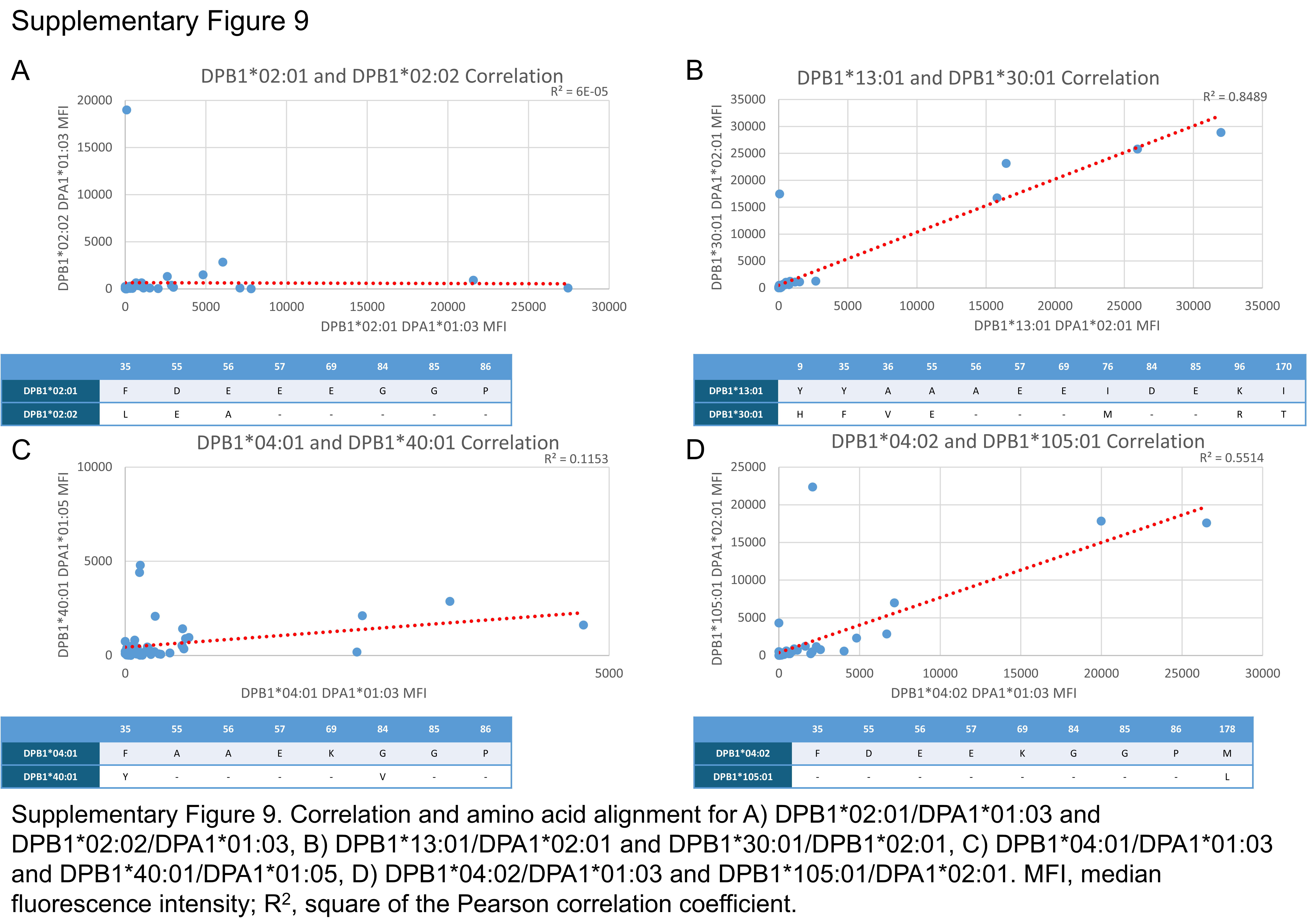

Supplement: Supplementary file 9 — Figure S9: Correlation and amino acid alignment for Explex/Standard panel DP alleles. [file TAN-108-e70797-s001.tif]

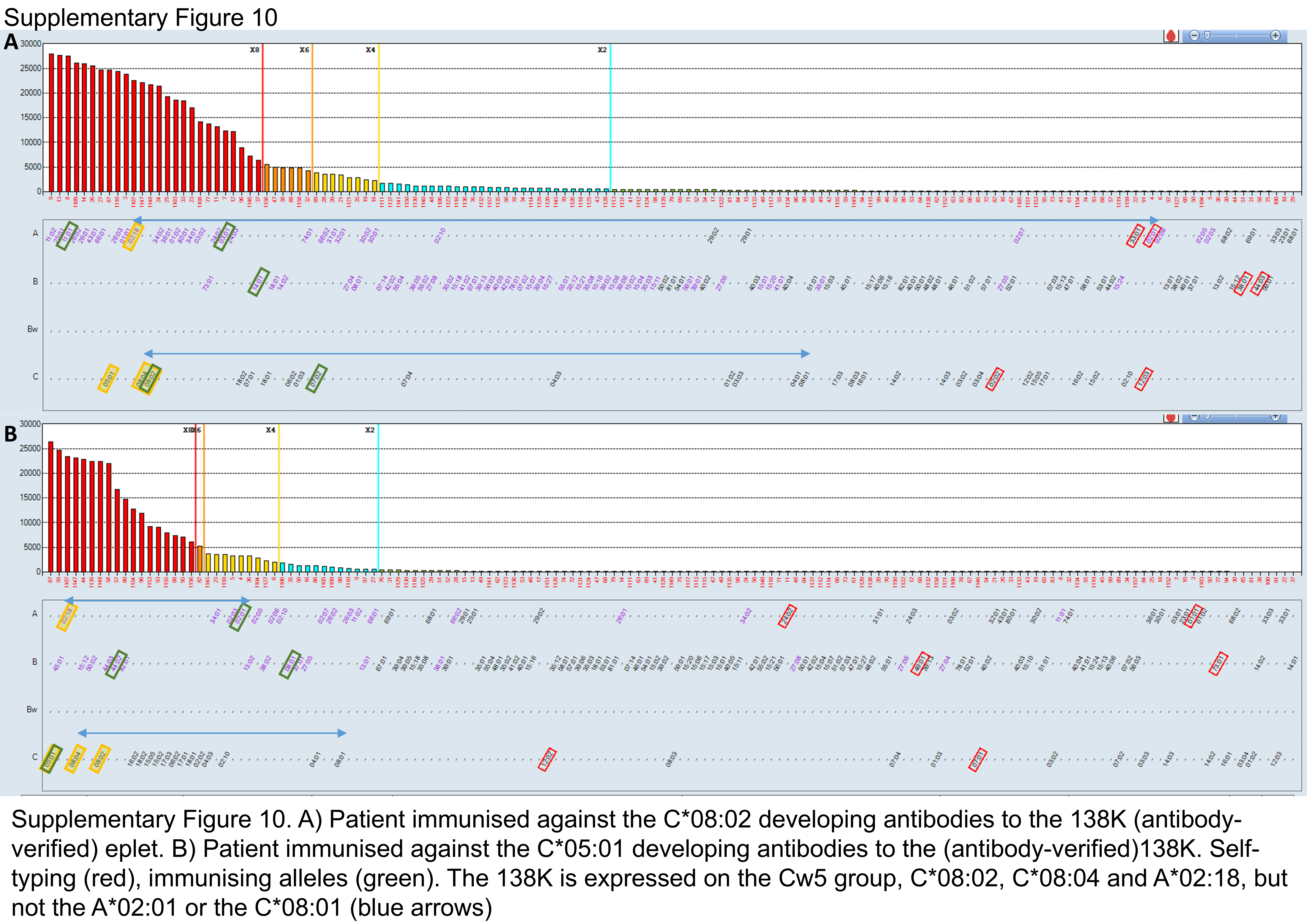

Supplement: Supplementary file 10 — Figure S10: Luminex profiles with reactivity to alleles sharing the 138K eplet. [file TAN-108-e70797-s016.tif]

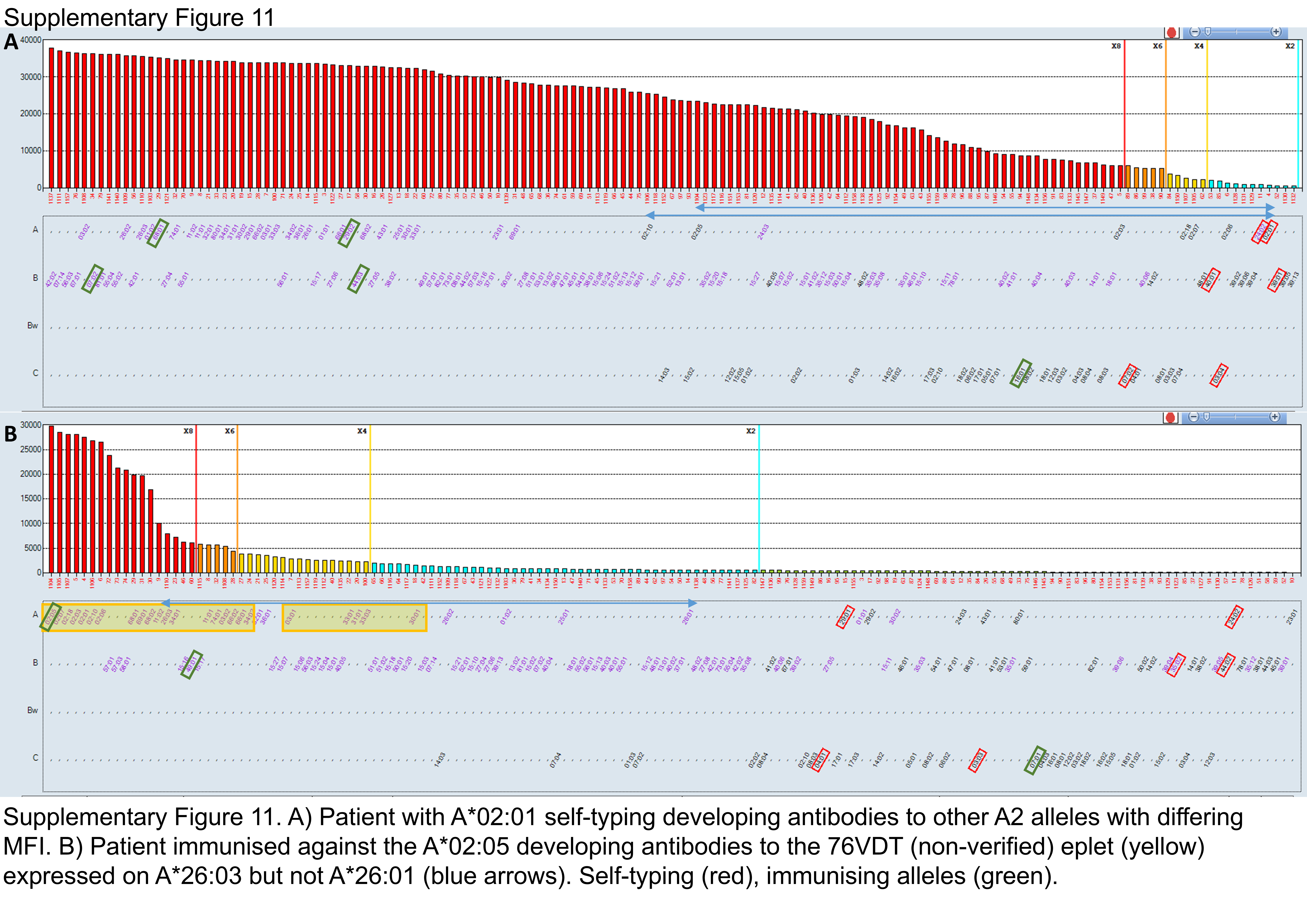

Supplement: Supplementary file 11 — Figure S11: Luminex profiles with varying reactivity to alleles within the A2 antigen group, and the A26 antigen group. [file TAN-108-e70797-s003.tif]

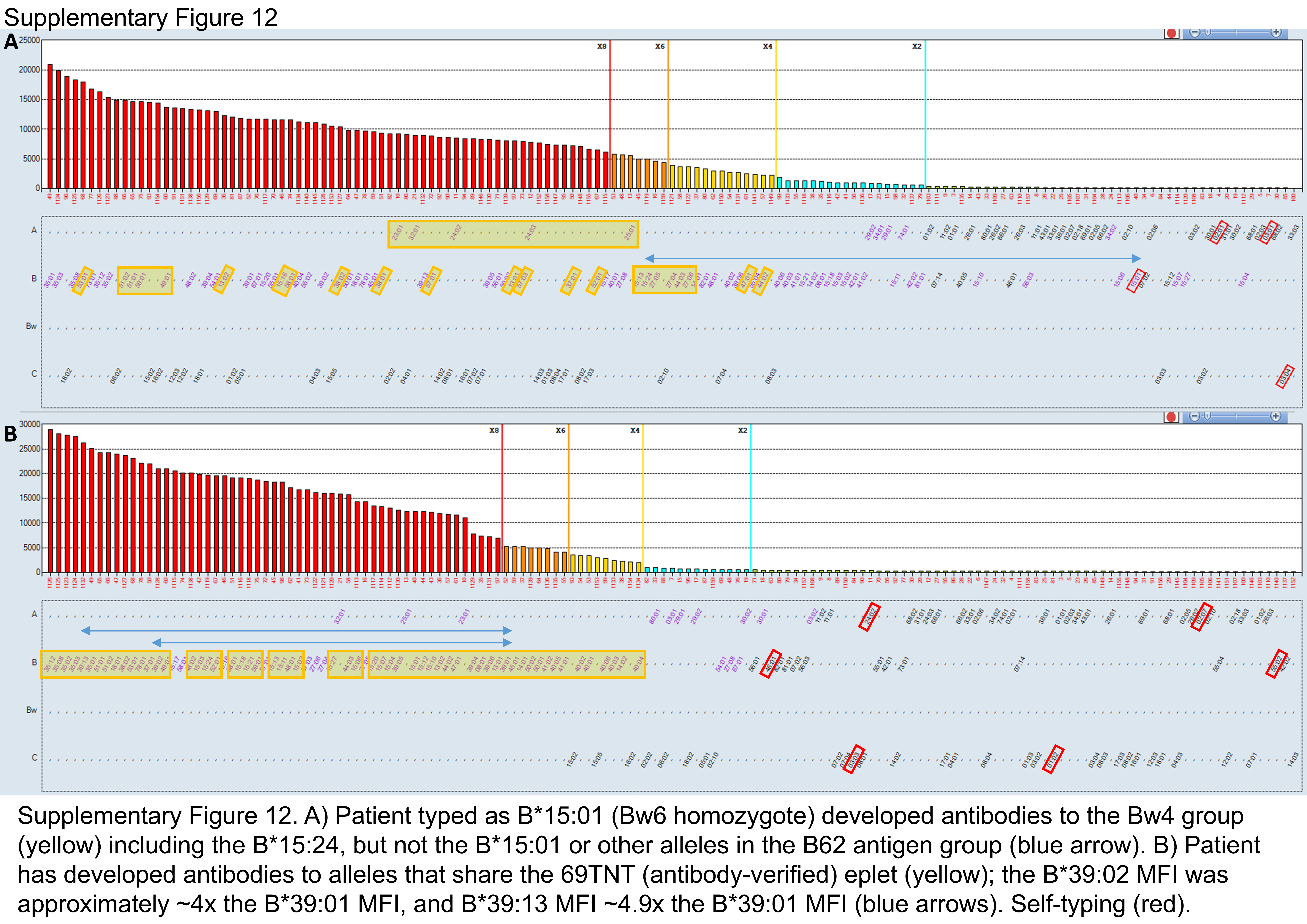

Supplement: Supplementary file 12 — Figure S12: Luminex profiles with varying reactivity to alleles within the B62 antigen group, and the B39 antigen group. [file TAN-108-e70797-s010.tif]

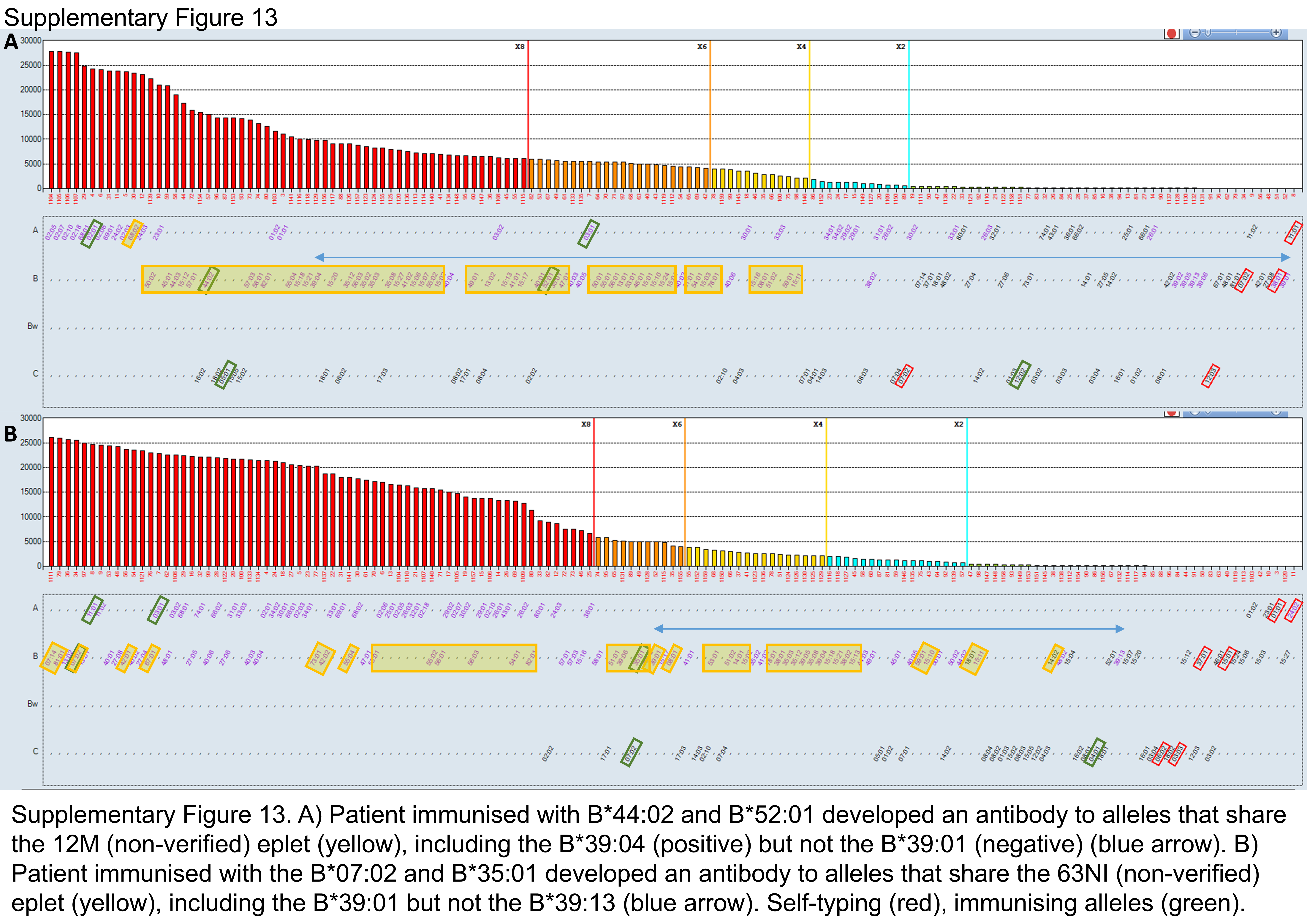

Supplement: Supplementary file 13 — Figure S13: Luminex profiles with reactivity to alleles sharing the 12M eplet, and 63NI eplet. [file TAN-108-e70797-s023.tif]

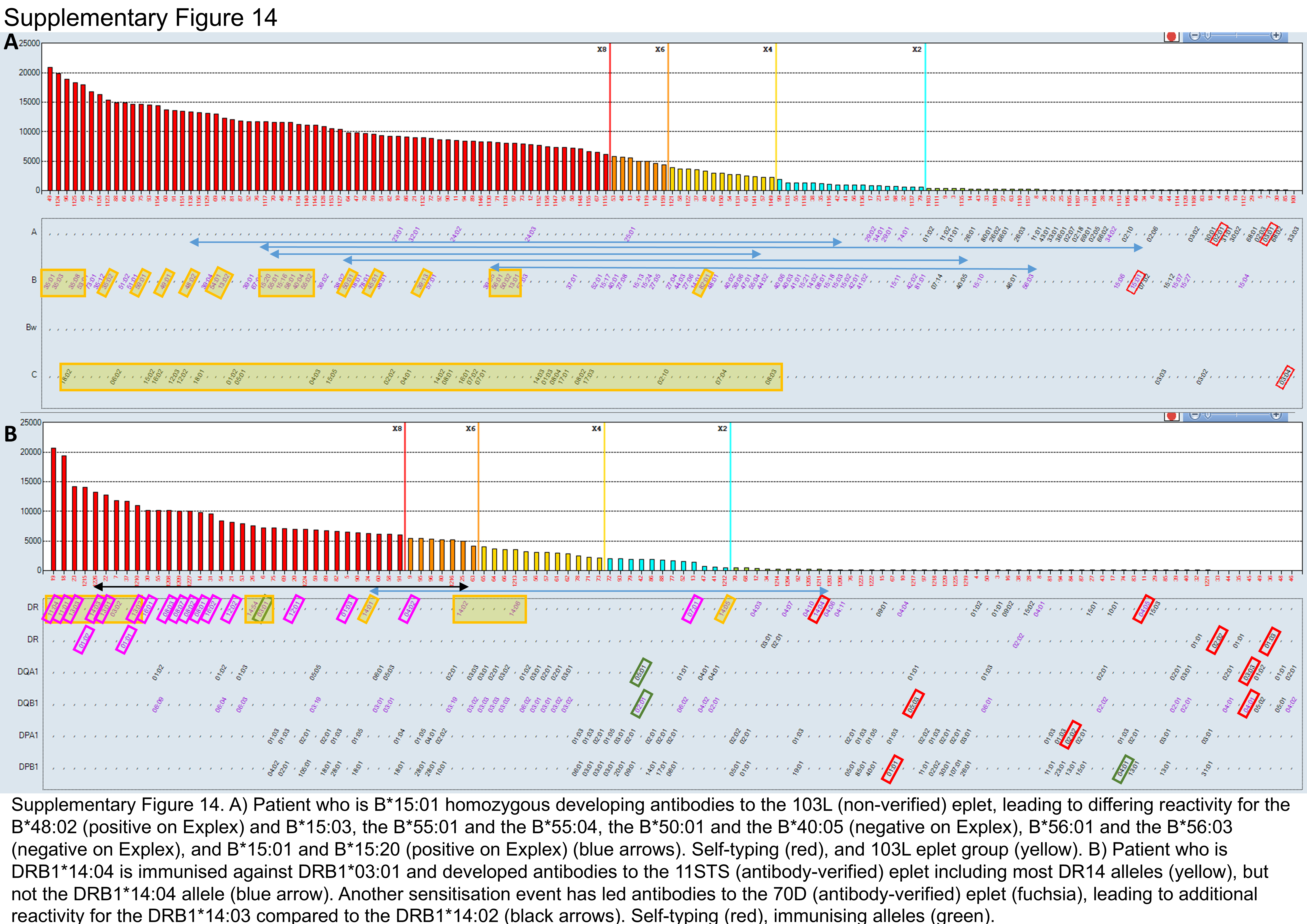

Supplement: Supplementary file 14 — Figure S14: Luminex profiles with reactivity to alleles sharing the 103L eplet, and the 11STS eplet. [file TAN-108-e70797-s027.tif]

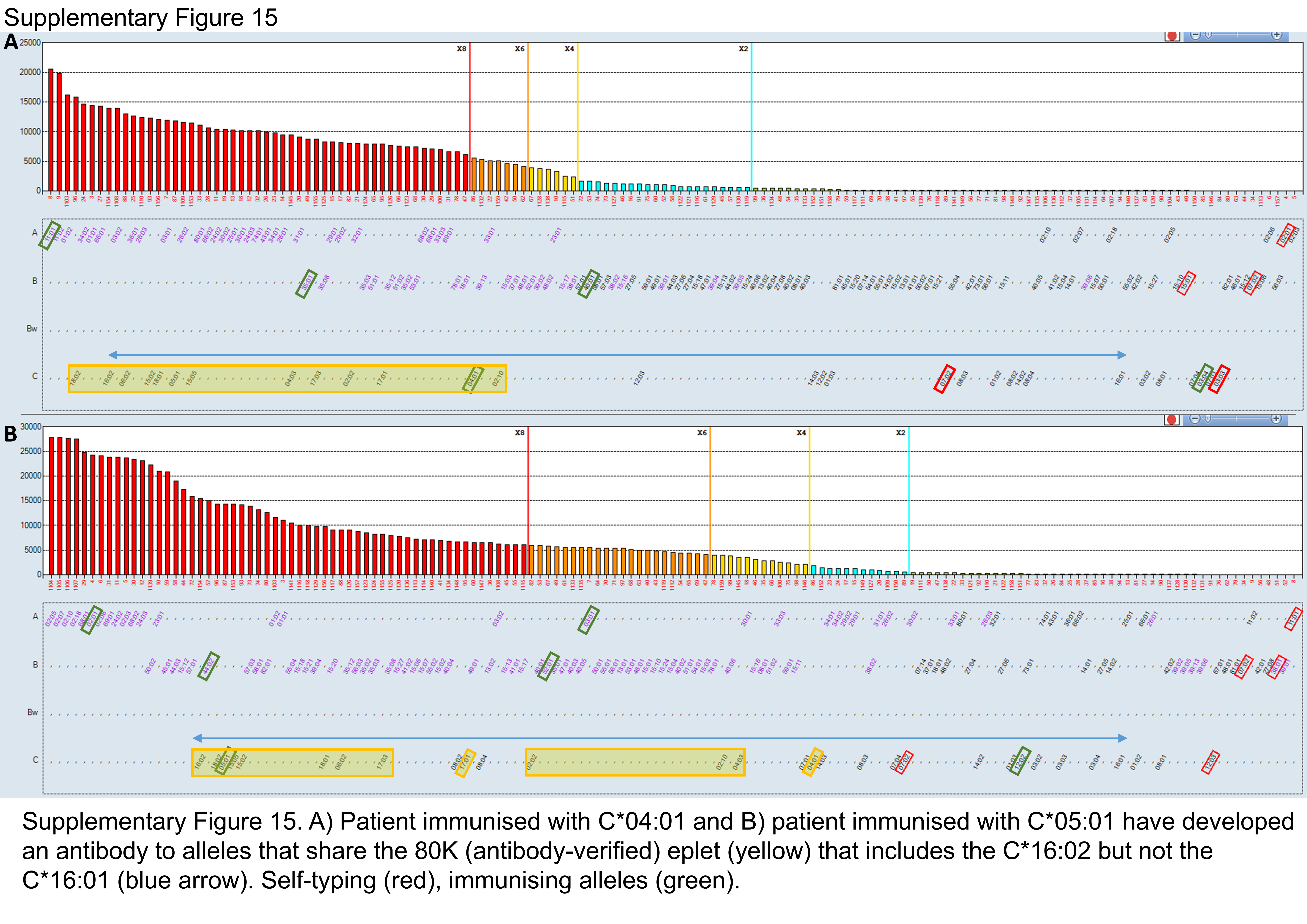

Supplement: Supplementary file 15 — Figure S15: Luminex profiles with reactivity to alleles sharing the 80K eplet. [file TAN-108-e70797-s013.tif]

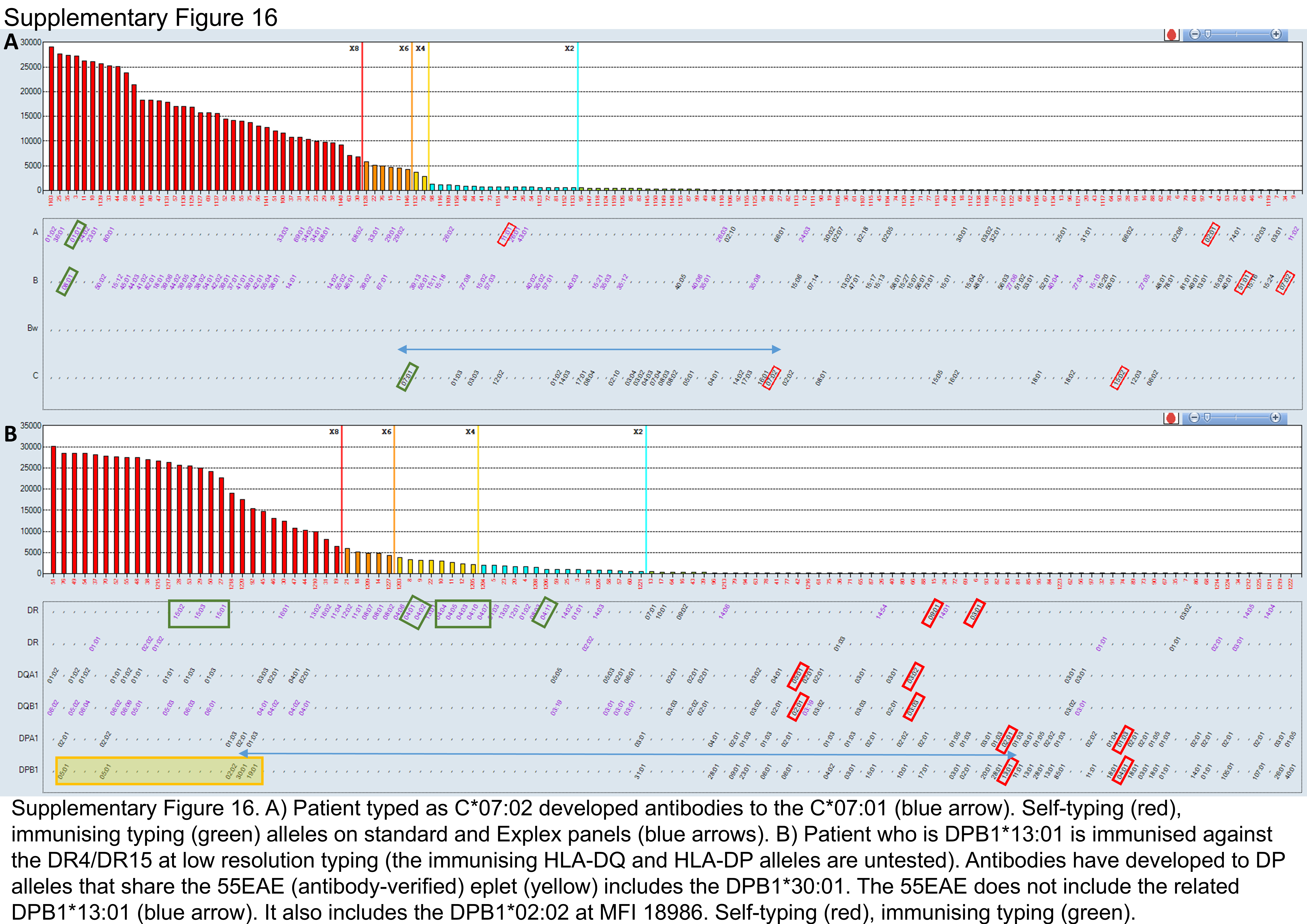

Supplement: Supplementary file 16 — Figure S16: Luminex profile with varying reactivity to alleles within the Cw7 antigen group, and Luminex profile with reactivity to alleles sharing the 55EAE eplet. [file TAN-108-e70797-s005.tif]

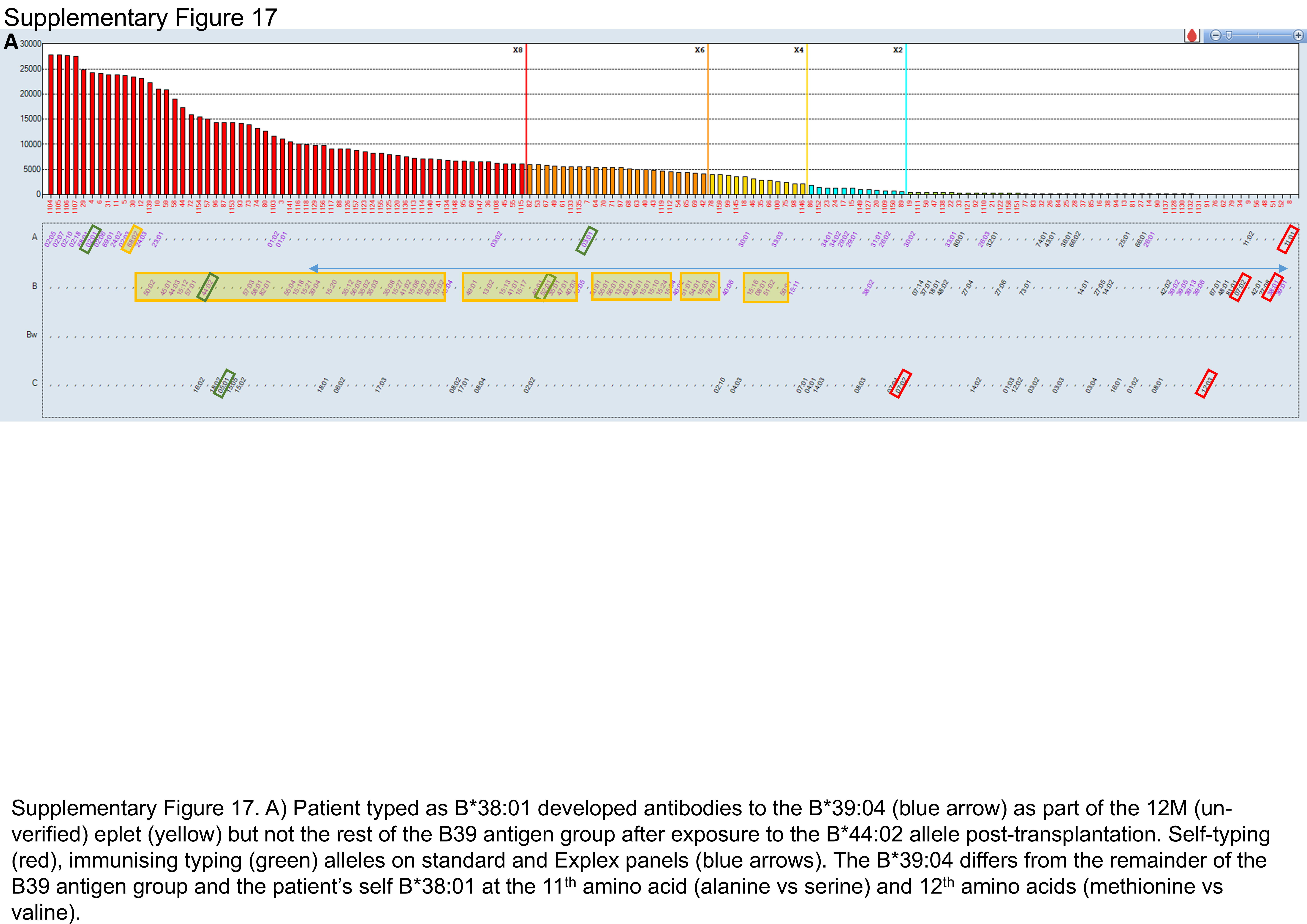

Supplement: Supplementary file 17 — Figure S17: Luminex profile with reactivity to alleles sharing the 12M eplet. [file TAN-108-e70797-s020.tif]

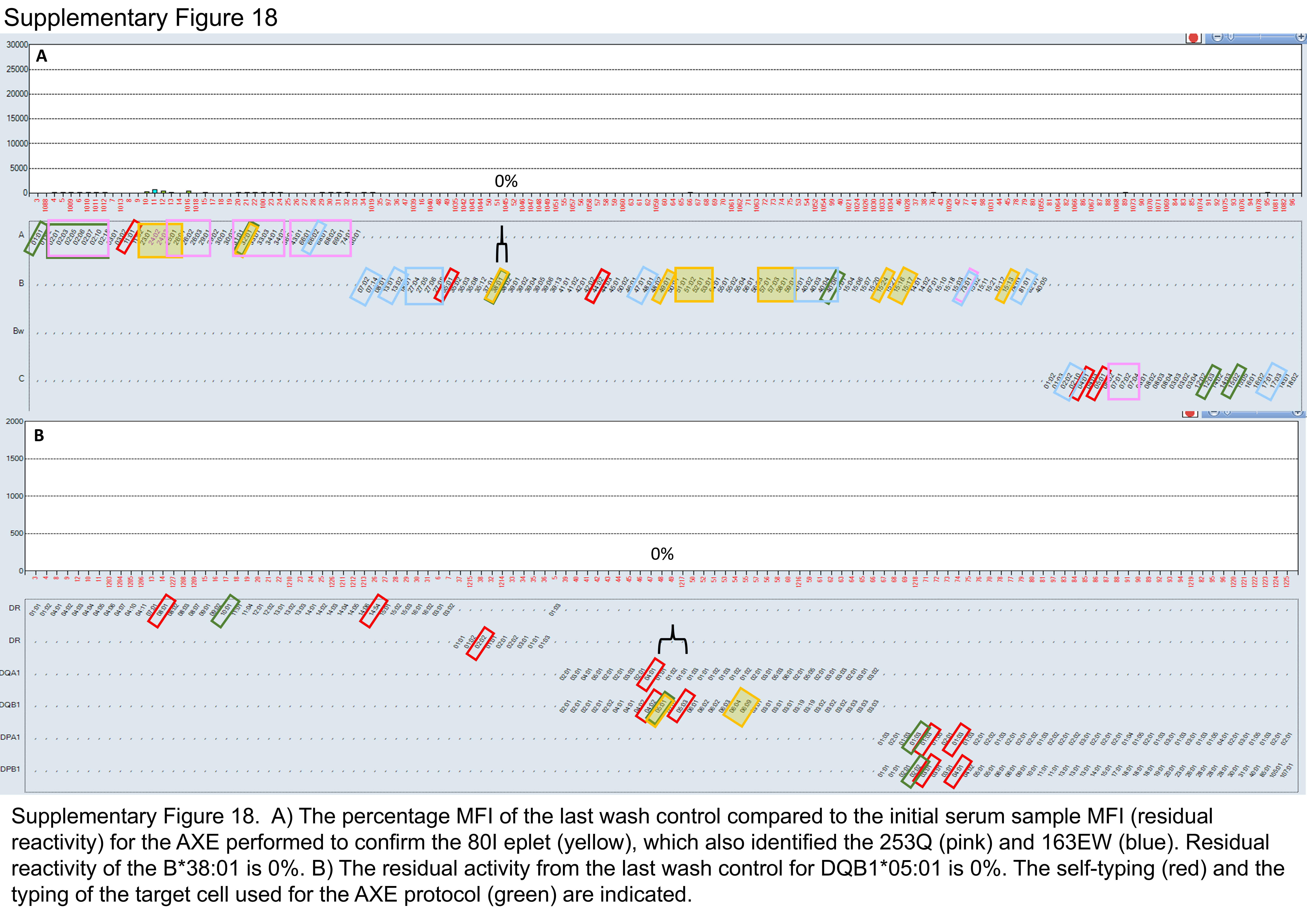

Supplement: Supplementary file 18 — Figure S18: AXE experiment confirming the reactivity to alleles sharing the 80I eplet. [file TAN-108-e70797-s011.tif]

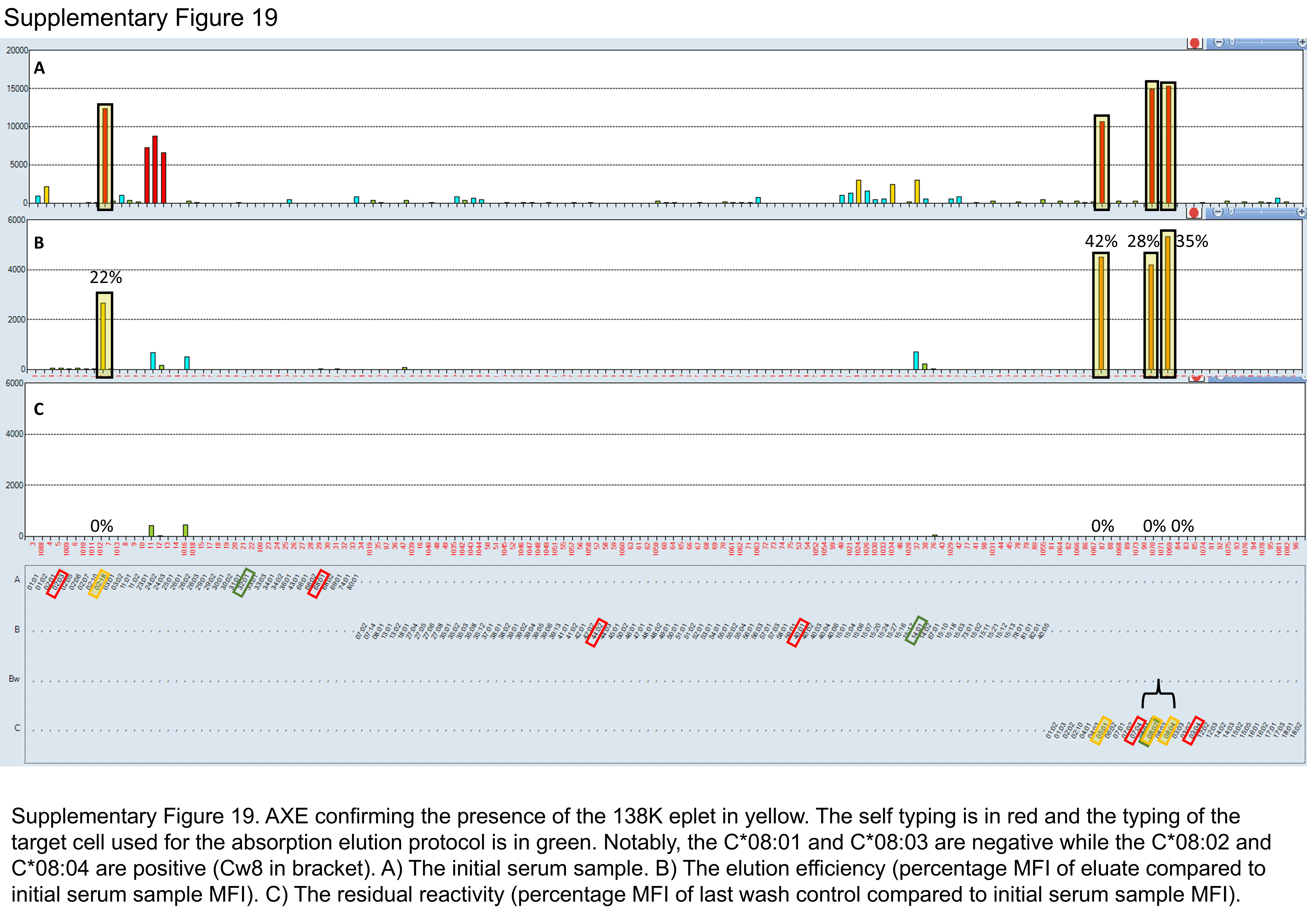

Supplement: Supplementary file 19 — Figure S19: AXE experiment confirming the reactivity to alleles sharing the 138K eplet. [file TAN-108-e70797-s029.tif]

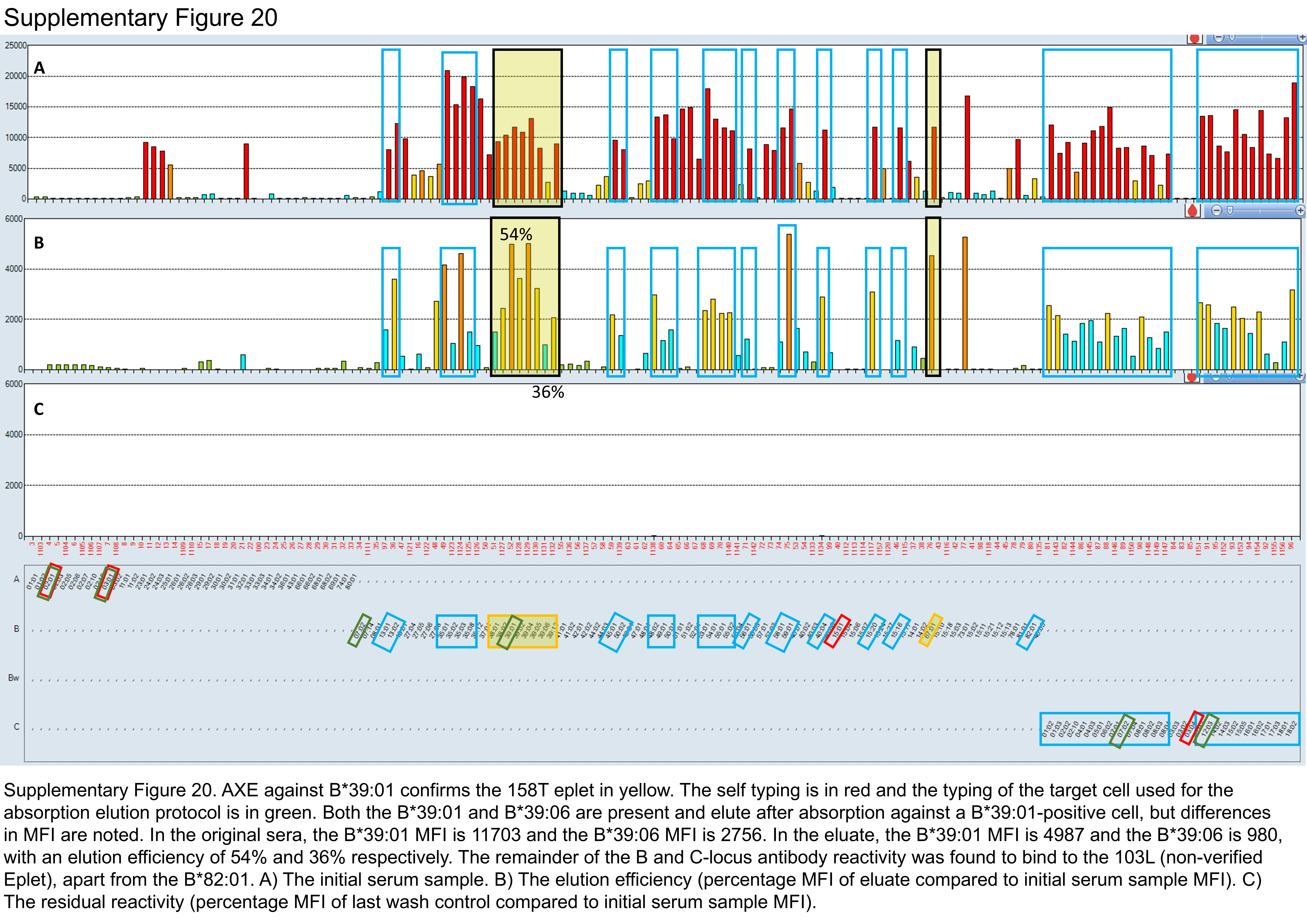

Supplement: Supplementary file 20 — Figure S20: AXE experiment confirming the reactivity to alleles sharing the 158T eplet. [file TAN-108-e70797-s006.tif]

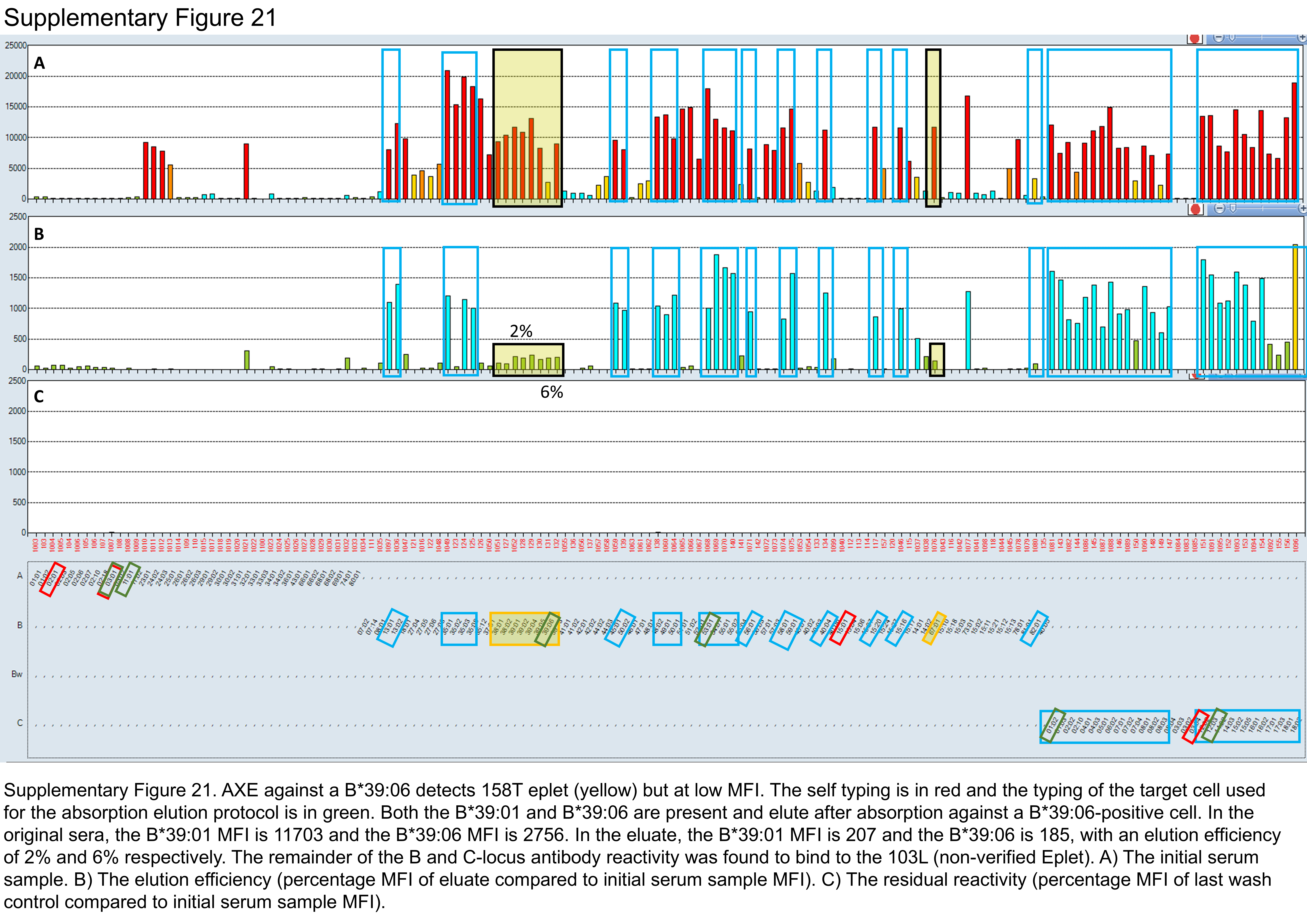

Supplement: Supplementary file 21 — Figure S21: AXE experiment confirming the reactivity to alleles sharing the 158T eplet. [file TAN-108-e70797-s021.tif]

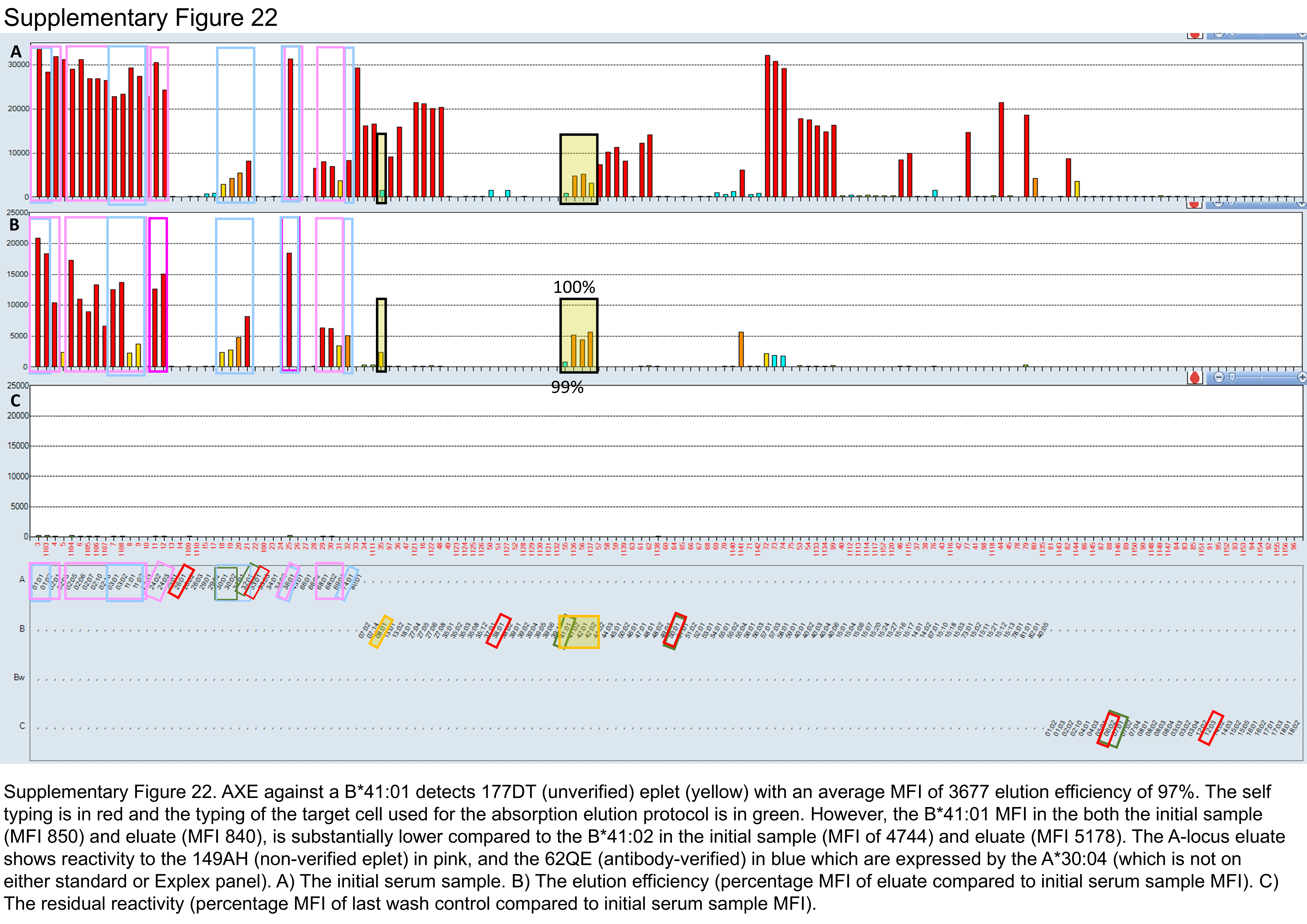

Supplement: Supplementary file 22 — Figure S22: AXE experiment confirming the reactivity to alleles sharing the 177DT eplet. [file TAN-108-e70797-s026.tif]

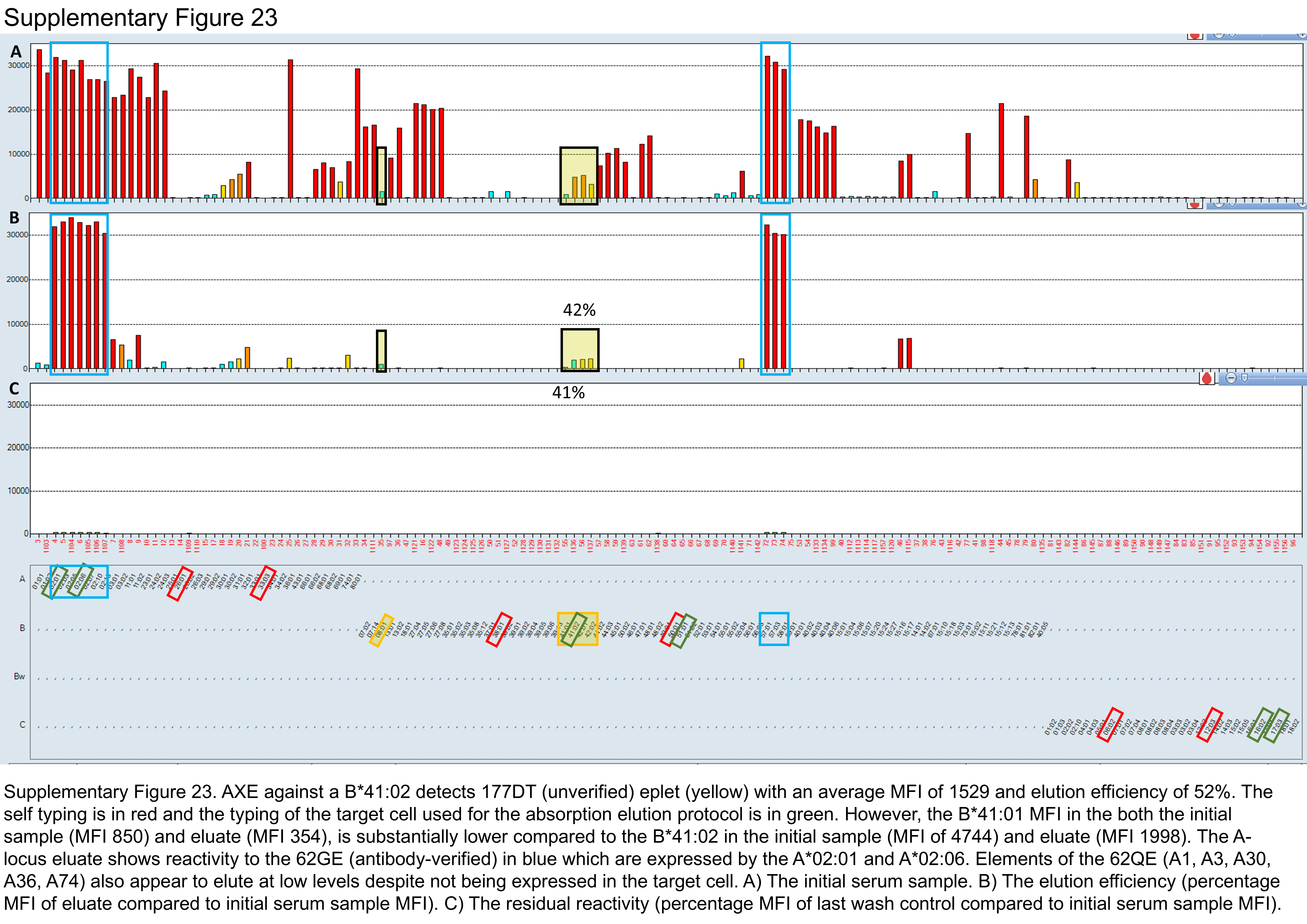

Supplement: Supplementary file 23 — Figure S23: AXE experiment confirming the reactivity to alleles sharing the 177DT eplet. [file TAN-108-e70797-s012.tif]

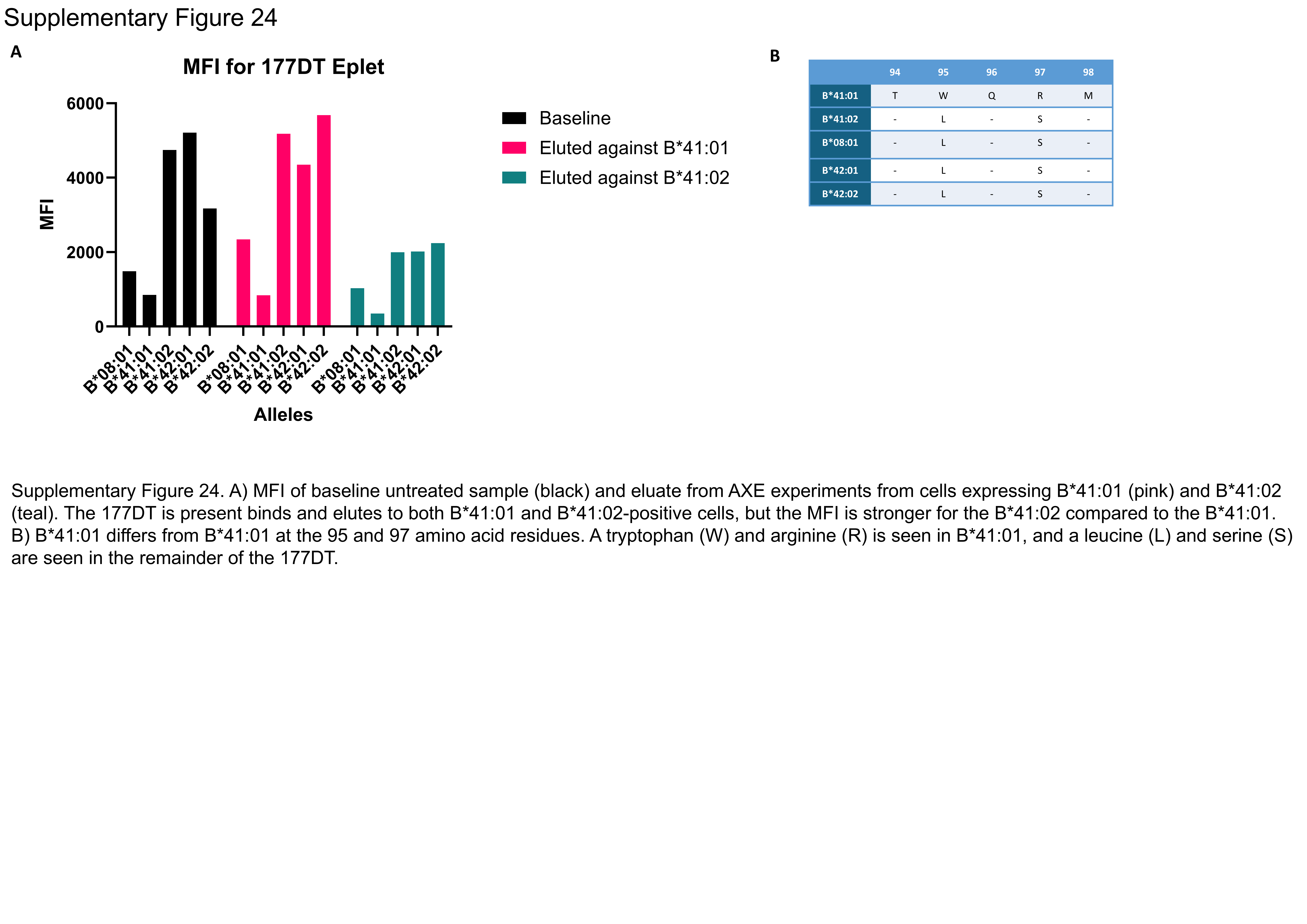

Supplement: Supplementary file 24 — Figure S24: MFI of serum and AXE experiments eluates targeting the 177DT. [file TAN-108-e70797-s008.tif]

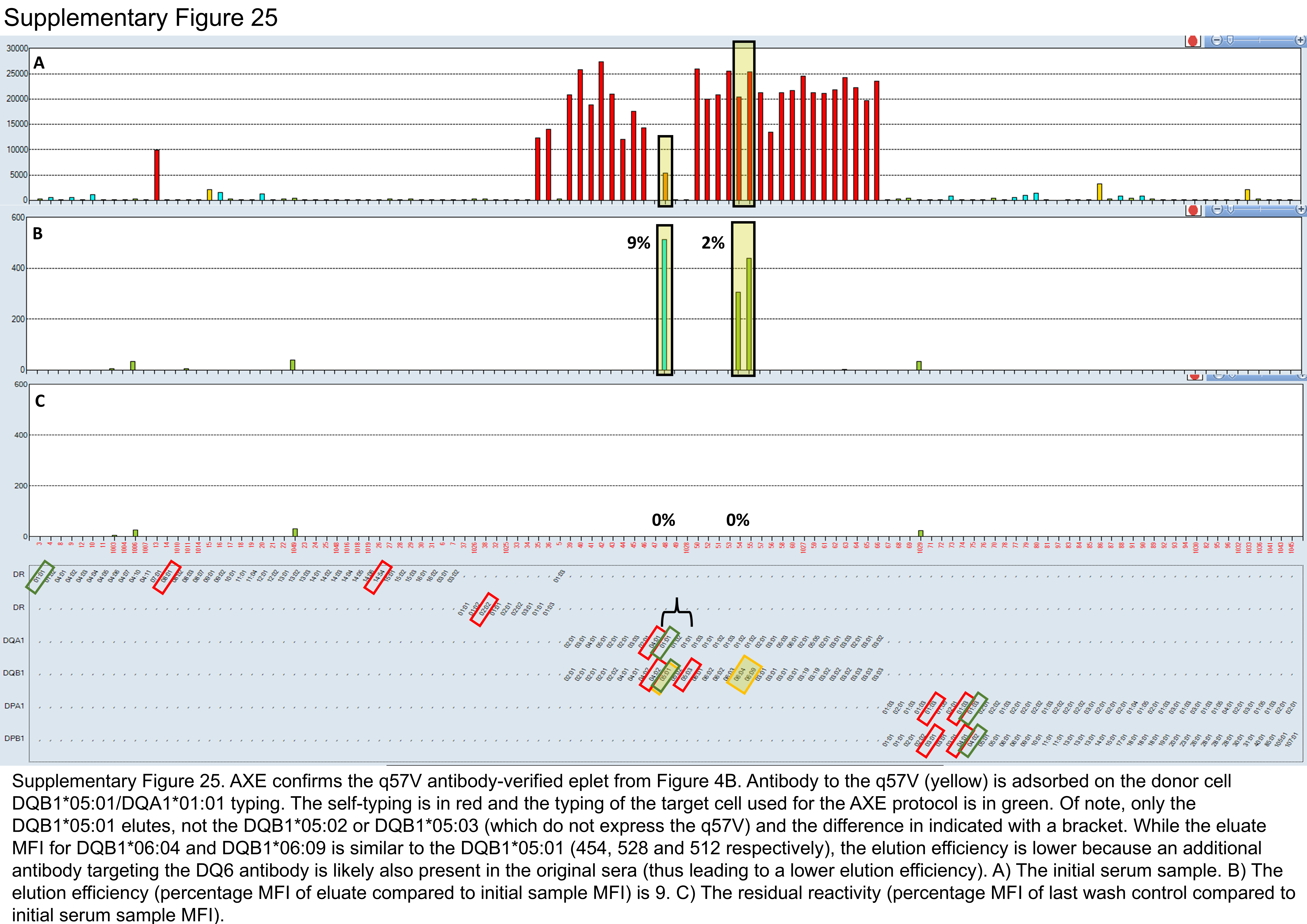

Supplement: Supplementary file 25 — Figure S25: AXE experiment confirming the reactivity to alleles sharing the q57V eplet. [file TAN-108-e70797-s022.tif]

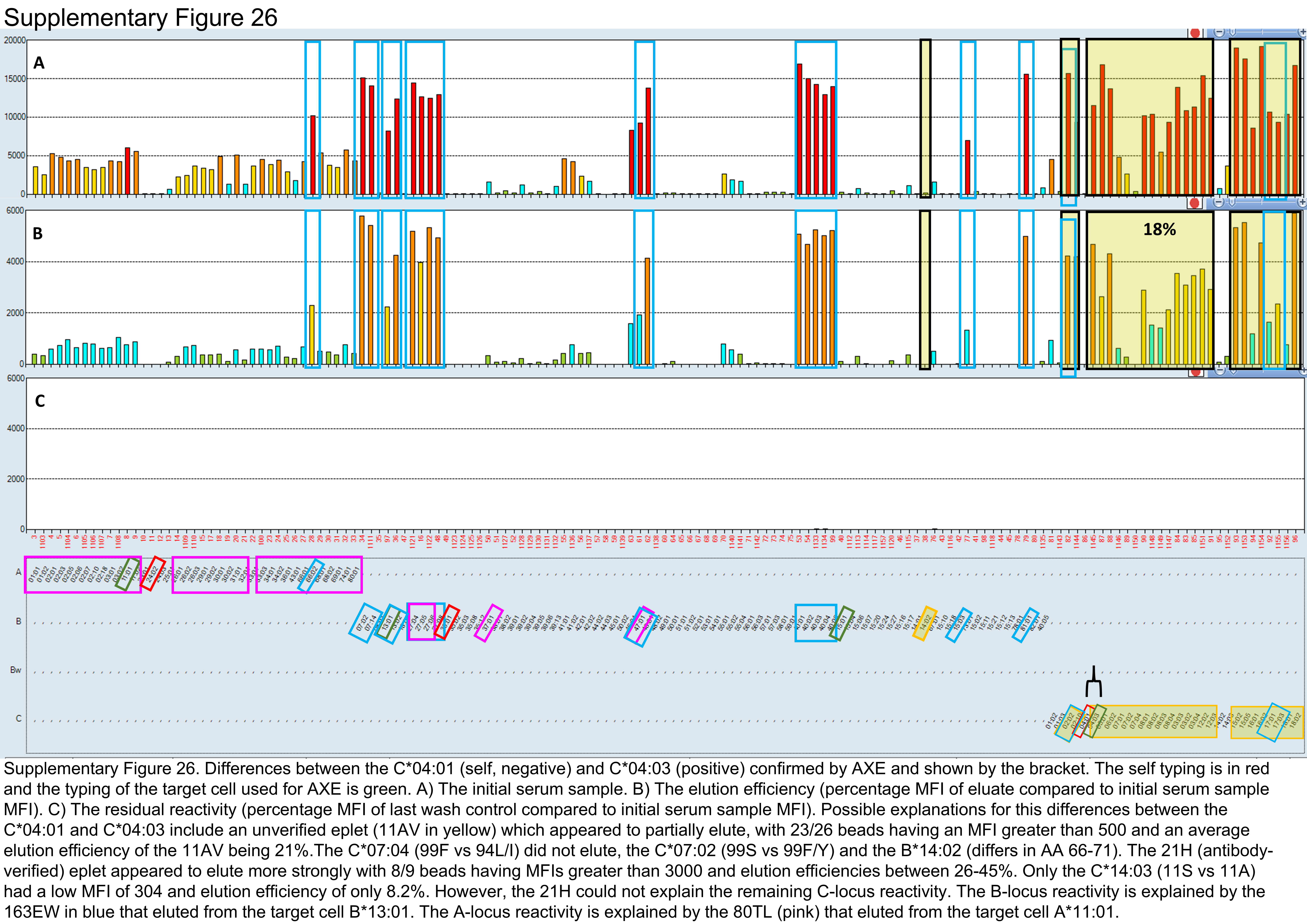

Supplement: Supplementary file 26 — Figure S26: AXE experiment confirming the different reactivity to alleles within the Cw4 antigen group. [file TAN-108-e70797-s017.tif]

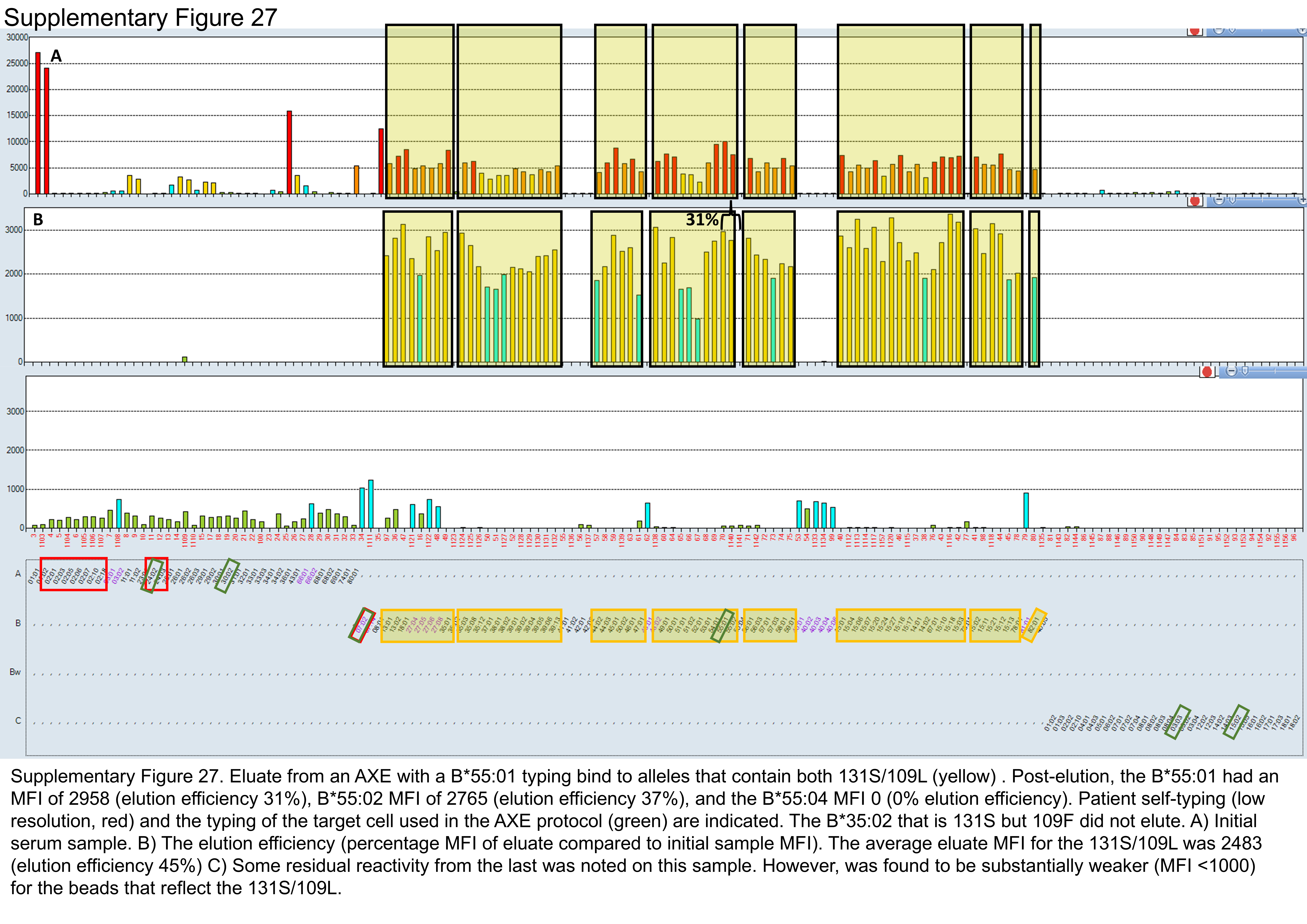

Supplement: Supplementary file 27 — Figure S27: AXE experiment confirming the reactivity to alleles sharing the combination of 131S/109L eplets. [file TAN-108-e70797-s004.tif]

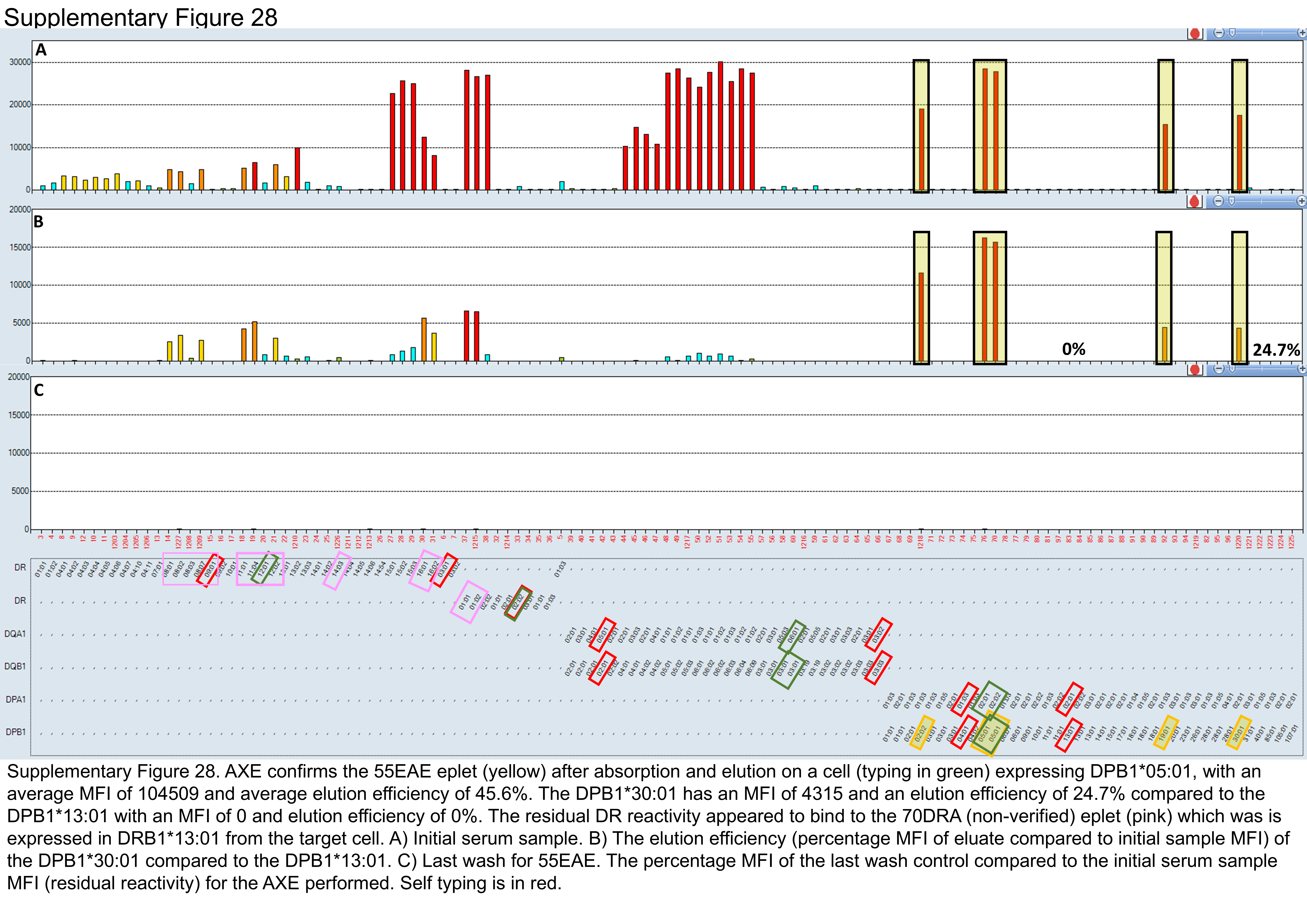

Supplement: Supplementary file 28 — Figure S28: AXE experiment confirming the reactivity to alleles sharing the 55EAE eplet. [file TAN-108-e70797-s024.tif]
