# Supplementary material for: Standard Single Antigen HLA Luminex Panels Predict Most, but Not All, Antibody Reactivity Against Alleles in Extended Panels
Source: HLA. 2026 Jul 15;108(1):e70797. doi: 10.1111/tan.70797 (PMC13370519; doi:10.1111/tan.70797)
Supplement: Supplementary file 29 — Table S1: Correlation between A locus Explex beads and their closest related bead on the standard panel. POS_POS is when both beads are > 4000, POS_NEG is when the MFI for standard bead > 4000 and MFI for Explex bead is < 1000, NEG_POS is when the MFI for standard bead is < 1000 and MFI for Explex bead is > 4000, NEG_NEG is when MFI for both SABS are < 1000. Questionable is when the MFI for one or both beads is between 1000 and 4000. Table S2: Correlation between B locus Explex beads and their closest related bead on the standard panel. POS_POS is when both beads are > 4000, POS_NEG is when the MFI for standard bead > 4000 and MFI for Explex bead is < 1000, NEG_POS is when the MFI for standard bead is < 1000 and MFI for Explex bead is > 4000, NEG_NEG is when MFI for both SABS are < 1000. Questionable is when the MFI for one or both beads is between 1000 and 4000. Table S3: Correlation between DR locus Explex beads and their closest related bead on the standard panel. POS_POS is when both beads are > 4000, POS_NEG is when the MFI for standard bead > 4000 and MFI for Explex bead is < 1000, NEG_POS is when the MFI for standard bead is < 1000 and MFI for Explex bead is > 4000, NEG_NEG is when MFI for both SABS are < 1000. Questionable is when the MFI for one or both beads is between 1000 and 4000. Table S4: Correlation between DQ locus Explex beads and their closest related bead on the standard panel. POS_POS is when both beads are > 4000, POS_NEG is when the MFI for standard bead > 4000 and MFI for Explex bead is < 1000, NEG_POS is when the MFI for standard bead is < 1000 and MFI for Explex bead is > 4000, NEG_NEG is when MFI for both SABS are < 1000. Questionable is when the MFI for one or both beads is between 1000 and 4000. Table S5: Correlation between C locus Explex beads and their closest related bead on the standard panel. POS_POS is when both beads are > 4000, POS_NEG is when the MFI for standard bead > 4000 and MFI for Explex bead is < 1000, NEG_POS is when th [file TAN-108-e70797-s019.zip › tan70797-sup-0030-Tables.docx]

SUPPLEMENTARY TABLE 1. Correlation Between A Locus Explex Beads and their Closest Related Bead on the Standard Panel. POS_POS is when both beads are >4000, POS_NEG is when the MFI for Standard Bead >4000 and MFI for Explex Bead is <1000, NEG_POS is when the MFI for Standard Bead is <1000 and MFI for Explex Bead is >4000, NEG_NEG is when MFI for both SABS are <1000. Questionable is when the MFI for one or both beads is between 1000-4000.

| **Standard Bead** | **Explex Bead** | **POS_POS** | **POS_NEG** | **NEG_POS** | **NEG_NEG** | **TOTAL** | **QUESTIONABLE** |
| --- | --- | --- | --- | --- | --- | --- | --- |
| A*01:01 | A*01:02 | 14 | 0 | 0 | 25 | 48 | 9 |
| A*02:01 | A*02:05 | 18 | 0 | 1 | 25 | 48 | 4 |
| A*02:01 | A*02:07 | 17 | 0 | 0 | 26 | 48 | 5 |
| A*02:01 | A*02:10 | 17 | 0 | 1 | 26 | 48 | 4 |
| A*02:01 | A*02:18 | 16 | 0 | 2 | 24 | 48 | 6 |
| A*03:01 | A*03:02 | 14 | 0 | 0 | 26 | 48 | 8 |
| A*26:01 | A*26:02 | 12 | 0 | 0 | 25 | 48 | 11 |
| A*26:01 | A*26:03 | 12 | 0 | 1 | 27 | 48 | 8 |

SUPPLEMENTARY TABLE 2. Correlation Between B Locus Explex Beads and their Closest Related Bead on the Standard Panel. POS_POS is when both beads are >4000, POS_NEG is when the MFI for Standard Bead >4000 and MFI for Explex Bead is <1000, NEG_POS is when the MFI for Standard Bead is <1000 and MFI for Explex Bead is >4000, NEG_NEG is when MFI for both SABS are <1000. Questionable is when the MFI for one or both beads is between 1000-4000.

| **Standard Bead** | **Explex Bead** | **POS_POS** | **POS_NEG** | **NEG_POS** | **NEG_NEG** | **TOTAL** | **QUESTIONABLE** |
| --- | --- | --- | --- | --- | --- | --- | --- |
| B*07:02 | B*07:14 | 16 | 0 | 0 | 23 | 48 | 9 |
| B*15:01 | B*15:04 | 11 | 0 | 0 | 28 | 48 | 9 |
| B*15:01 | B*15:06 | 13 | 0 | 0 | 27 | 48 | 8 |
| B*15:01 | B*15:07 | 13 | 0 | 0 | 27 | 48 | 8 |
| B*15:16 | B*15:17 | 15 | 0 | 0 | 19 | 48 | 14 |
| B*15:10 | B*15:18 | 13 | 0 | 0 | 24 | 48 | 11 |
| B*15:01 | B*15:20 | 13 | 0 | 1 | 24 | 48 | 10 |
| B*15:02 | B*15:21 | 13 | 0 | 0 | 26 | 48 | 9 |
| B*15:01 | B*15:24 | 11 | 0 | 2 | 24 | 48 | 11 |
| B*15:01 | B*15:27 | 13 | 0 | 0 | 27 | 48 | 8 |
| B*27:05 | B*27:04 | 19 | 0 | 0 | 15 | 48 | 14 |
| B*27:05 | B*27:06 | 18 | 0 | 0 | 19 | 48 | 11 |
| B*35:01 | B*35:02 | 14 | 0 | 0 | 27 | 48 | 7 |
| B*35:01 | B*35:03 | 14 | 0 | 0 | 28 | 48 | 6 |
| B*35:01 | B*35:08 | 13 | 0 | 0 | 28 | 48 | 7 |
| B*35:01 | B*35:12 | 14 | 0 | 0 | 27 | 48 | 7 |
| B*38:01 | B*38:02 | 13 | 1 | 0 | 21 | 48 | 13 |
| B*39:01 | B*39:02 | 12 | 0 | 0 | 24 | 48 | 12 |
| B*39:01 | B*39:04 | 11 | 0 | 1 | 25 | 48 | 11 |
| B*39:01 | B*39:05 | 11 | 0 | 0 | 25 | 48 | 12 |
| B*39:01 | B*39:06 | 10 | 0 | 0 | 23 | 48 | 15 |
| B*39:01 | B*39:13 | 11 | 1 | 0 | 25 | 48 | 11 |
| B*40:02 | B*40:03 | 19 | 0 | 0 | 20 | 48 | 9 |
| B*40:02 | B*40:04 | 19 | 0 | 0 | 20 | 48 | 9 |
| B*50:01 | B*40:05 | 13 | 1 | 0 | 23 | 48 | 11 |
| B*41:01 | B*41:02 | 17 | 0 | 1 | 18 | 48 | 12 |
| B*42:01 | B*42:02 | 12 | 0 | 0 | 24 | 48 | 12 |
| B*15:03 | B*48:02 | 9 | 1 | 1 | 27 | 48 | 10 |
| B*45:01 | B*50:02 | 19 | 0 | 0 | 24 | 48 | 5 |
| B*55:01 | B*55:02 | 14 | 0 | 0 | 20 | 48 | 14 |
| B*55:01 | B*55:04 | 13 | 0 | 1 | 20 | 48 | 14 |
| B*56:01 | B*56:03 | 12 | 2 | 0 | 27 | 48 | 7 |

SUPPLEMENTARY TABLE 3. Correlation Between DR Locus Explex Beads and their Closest Related Bead on the Standard Panel. POS_POS is when both beads are >4000, POS_NEG is when the MFI for Standard Bead >4000 and MFI for Explex Bead is <1000, NEG_POS is when the MFI for Standard Bead is <1000 and MFI for Explex Bead is >4000, NEG_NEG is when MFI for both SABS are <1000. Questionable is when the MFI for one or both beads is between 1000-4000.

| **Standard Bead** | **Explex**  **Bead** | **POS_POS** | **POS_NEG** | **NEG_POS** | **NEG_NEG** | **TOTAL** | **QUESTIONABLE** |
| --- | --- | --- | --- | --- | --- | --- | --- |
| DRB1*04:03 | DRB1*04:06 | 8 | 0 | 0 | 28 | 50 | 14 |
| DRB1*04:03 | DRB1*04:07 | 7 | 0 | 0 | 29 | 50 | 14 |
| DRB1*04:01 | DRB1*04:10 | 7 | 0 | 0 | 30 | 50 | 13 |
| DRB1*04:03 | DRB1*04:11 | 7 | 0 | 0 | 30 | 50 | 13 |
| DRB1*08:01 | DRB1*08:02 | 15 | 0 | 0 | 28 | 50 | 7 |
| DRB1*08:01 | DRB1*08:03 | 15 | 0 | 0 | 26 | 50 | 9 |
| DRB1*08:01 | DRB1*08:07 | 16 | 0 | 0 | 27 | 50 | 7 |
| DRB1*13:01 | DRB1*13:02 | 15 | 0 | 0 | 28 | 50 | 7 |
| DRB1*14:02 | DRB1*14:03 | 13 | 1 | 1 | 28 | 50 | 7 |
| DRB1*14:01 | DRB1*14:04 | 17 | 2 | 0 | 26 | 50 | 5 |
| DRB1*14:01 | DRB1*14:05 | 15 | 2 | 0 | 26 | 50 | 7 |
| DRB1*14:02 | DRB1*14:06 | 11 | 0 | 0 | 33 | 50 | 6 |
| DRB3*02:02 | DRB3*02:01 | 7 | 0 | 0 | 36 | 50 | 7 |
| DRB5*01:01 | DRB5*01:02 | 18 | 0 | 0 | 26 | 50 | 6 |

SUPPLEMENTARY TABLE 4. Correlation Between DQ Locus Explex Beads and their Closest Related Bead on the Standard Panel. POS_POS is when both beads are >4000, POS_NEG is when the MFI for Standard Bead >4000 and MFI for Explex Bead is <1000, NEG_POS is when the MFI for Standard Bead is <1000 and MFI for Explex Bead is >4000, NEG_NEG is when MFI for both SABS are <1000. Questionable is when the MFI for one or both beads is between 1000-4000.

| **Standard Bead** | **Explex Bead** | **POS_POS** | **POS_NEG** | **NEG_POS** | **NEG_NEG** | **TOTAL** | **QUESTIONABLE** |
| --- | --- | --- | --- | --- | --- | --- | --- |
| DQB1*03:01 DQA1*02:01 | DQB1*03:19 DQA1*02:01 | 15 | 0 | 0 | 27 | 50 | 8 |
| DQB1*05:01 DQA1*01:01 | DQB1*05:03 DQA1*01:01 | 10 | 1 | 0 | 31 | 50 | 8 |

SUPPLEMENTARY TABLE 5. Correlation Between C Locus Explex Beads and their Closest Related Bead on the Standard Panel. POS_POS is when both beads are >4000, POS_NEG is when the MFI for Standard Bead >4000 and MFI for Explex Bead is <1000, NEG_POS is when the MFI for Standard Bead is <1000 and MFI for Explex Bead is >4000, NEG_NEG is when MFI for both SABS are <1000. Questionable is when the MFI for one or both beads is between 1000-4000.

| **Standard Bead** | **Explex Bead** | **POS_POS** | **POS_NEG** | **NEG_POS** | **NEG_NEG** | **TOTAL** | **QUESTIONABLE** |
| --- | --- | --- | --- | --- | --- | --- | --- |
| C*01:02 | C*01:03 | 6 | 1 | 1 | 31 | 48 | 9 |
| C*02:02 | C*02:10 | 9 | 0 | 0 | 26 | 48 | 13 |
| C*04:01 | C*04:03 | 10 | 0 | 1 | 32 | 48 | 5 |
| C*07:02 | C*07:01 | 7 | 0 | 1 | 30 | 48 | 10 |
| C*07:02 | C*07:04 | 3 | 0 | 0 | 33 | 48 | 12 |
| C*08:01 | C*08:02 | 6 | 0 | 4 | 33 | 48 | 5 |
| C*08:01 | C*08:03 | 5 | 0 | 0 | 39 | 48 | 4 |
| C*08:01 | C*08:04 | 6 | 0 | 5 | 32 | 48 | 5 |
| C*12:03 | C*12:02 | 8 | 0 | 0 | 32 | 48 | 8 |
| C*14:02 | C*14:03 | 6 | 0 | 2 | 28 | 48 | 12 |
| C*15:02 | C*15:05 | 14 | 0 | 0 | 28 | 48 | 6 |
| C*16:01 | C*16:02 | 6 | 0 | 6 | 30 | 48 | 6 |
| C*17:01 | C*17:03 | 13 | 0 | 0 | 29 | 48 | 6 |
| C*18:02 | C*18:01 | 17 | 0 | 0 | 29 | 48 | 2 |

SUPPLEMENTARY TABLE 6. Correlation Between DP Locus Explex Beads and their Closest Related Bead on the Standard Panel. POS_POS is when both beads are >4000, POS_NEG is when the MFI for Standard Bead >4000 and MFI for Explex Bead is <1000, NEG_POS is when the MFI for Standard Bead is <1000 and MFI for Explex Bead is >4000, NEG_NEG is when MFI for both SABS are <1000. Questionable is when the MFI for one or both beads is between 1000-4000.

| **Standard Bead** | **Explex Bead** | **POS_POS** | **POS_NEG** | **NEG_POS** | **NEG_NEG** | **TOTAL** | **QUESTIONABLE** |
| --- | --- | --- | --- | --- | --- | --- | --- |
| DPB1*02:01 DPA1*01:03 | DPB1*02:02 DPA1*01:03 | 0 | 4 | 1 | 36 | 50 | 9 |
| DPB1*01:01 DPA1*01:03 | DPB1*26:01 DPA1*03:01 | 4 | 0 | 0 | 39 | 50 | 7 |
| DPB1*13:01 DPA1*02:01 | DPB1*30:01 DPA1*02:01 | 4 | 0 | 1 | 40 | 50 | 5 |
| DPB1*01:01 DPA1*01:03 | DPB1*31:01 DPA1*03:01 | 4 | 0 | 0 | 34 | 50 | 12 |
| DPB1*04:01 DPA1*01:03 | DPB1*40:01 DPA1*01:05 | 0 | 0 | 2 | 42 | 50 | 6 |
| DPB1*01:01 DPA1*01:03 | DPB1*85:01 DPA1*01:03 | 4 | 0 | 0 | 37 | 50 | 9 |
| DPB1*04:02 DPA1*01:03 | DPB1*105:01 DPA1*02:01 | 3 | 1 | 1 | 36 | 50 | 9 |
| DPB1*13:01 DPA1*02:01 | DPB1*107:01 DPA1*02:01 | 4 | 0 | 0 | 41 | 50 | 5 |

SUPPLEMENTARY TABLE 7. Demographics and Known Plausible Sensitisation History of Highly Sensitised Cohort

| **Demographics and Sensitisation History** | **Percentage (%)** |
| --- | --- |
| Median Age  (Interquartile Range)  [Range] | 51.5  (41-58)  [23-74] |
| Sex (Female) | 51.52 |
| Sensitisation by Pregnancy | 36.36 |
| Sensitisation by Transplant | 80.30 |
| Sensitisation by Transfusion | 65.15 |
| No Known Sensitisation Event | 3.03 |

SUPPLEMENTARY TABLE 9. Class I Eplets Predicted to Split Antigen Groups Or Serotypes

| **Eplet** | **Standard Beads** | **Explex beads** | **Positive**  **alleles** | **Negative alleles** |
| --- | --- | --- | --- | --- |
| **21H** | C*02:02, C*03:02, C*03:03, C*03:04, C*15:02 | C*02:10, C*04:03, C*14:03, C*15:05, | C*14:03 C*04:03 | C*14:02 C*04:01 |
| **65QKR** | B*46:01, C*01:02, C*02:02, C*03:02, C*03:03, C*03:04, C*04:01, C*05:01, C*06:02, C*07:02, C*08:01, C*12:03, C*14:02, C*16:01, C*17:01, C*18:02 | C*01:03, C*02:10, C*04:03, C*07:04, C*08:02, C*08:03, C*08:04, C*12:02, C*14:03, C*16:02, C*17:03, C*18:01 | C*07:02 C*07:04 | C*07:01 |
| **71TTS** | B*08:01, B*15:01, B*15:02, B*15:03, B*15:10, B*15:11, B*15:12, B*18:01, B*35:01, B*39:01, B*40:01, B*40:02, B*40:06, B*41:01, B*45:01, B*48:01, B*50:01, B*78:01 | B*15:04, B*15:06, B*15:07, B*15:18, B*15:20, B*15:21, B*15:27, B*35:02, B*35:03, B*35:08, B*35:12, B*39:02, B*39:04, B*39:05, B*39:06, B*39:13, B*40:03, B*40:04, B*40:05, B*41:02, B*48:02, B*50:02 | B62 | B*15:24 |
| **73TVS** | B*46:01, C*01:02, C*03:02, C*03:03, C*03:04, C*08:01, C*14:02, C*16:01 | C*01:03, C*08:02, C*08:03, C*08:04, C*14:03 | C*16:01 | C*16:02 |
| **76ANT** | A*01:01, A*26:01, A*29:01, A*29:02, A*36:01, A*43:01, A*80:01 | A*01:02, A*26:02 | A*26:01, A*26:02 | A*26:03 |
| **76ESN** | B*07:02, B*08:01, B*15:01, B*15:02, B*15:03, B*15:10, B*15:11, B*15:12, B*18:01, B*27:08, B*35:01, B*39:01, B*40:01, B*40:02, B*40:06, B*41:01, B*42:01, B*45:01, B*48:01, B*50:01, B*54:01, B*55:01, B*56:01, B*67:01, B*78:01, B*81:01, B*82:01 | B*07:14, B*15:04, B*15:06, B*15:07, B*15:18, B*15:21, B*15:21, B*15:27, B*35:02, B*35:03, B*35:08, B*35:12, B*39:02, B*39:04, B*39:05, B*39:06, B*39:13, B*40:03, B*40:04, B*40:05, B*41:02, B*42:02, B*48:02, B*50:02, B*55:02, B*55:04, B*56:03 | B62 | B*15:24 |
| **76VRN** | B*46:01, B*73:01, C*01:02, C*03:02, C*03:03, C*03:04, C*07:02, C*08:01, C*12:03, C*14:02, C*16:01 | C*01:03, C*07:01, C*07:04, C*08:02, C*08:03, C*08:04, C*12:02, C*14:03 | C*16:01 | C*16:02 |
| **80I** | A*23:01, A*24:02, A*24:03, A*25:01, A*32:01, B*15:13, B*15:16, B*38:01, B*49:01, B*51:01, B*51:02, B*52:01, B*53:01, B*57:01, B*57:03, B*58:01, B*59:01 | B*15:17, B*15:24 | B*15:24, B*38:01 | B62, B*38:02 |
| **80K** | C*02:02, C*04:01, C*05:01, C*06:02, C*15:02, C*17:01, C*18:02 | C*02:10, C*04:03, C*15:05, C*16:02, C*17:03, C*18:01 | C*16:02 | C*16:01 |
| **80N** | B*07:02, B*08:01, B*15:01, B*15:02, B*15:03, B*15:10, B*15:11, B*15:12, B*18:01, B*27:08, B*35:01, B*39:01, B*40:01, B*40:02, B*40:06, B*41:01, B*42:01, B*45:01, B*46:01, B*48:01, B*50:01, B*54:01, B*55:01, B*56:01, B*67:01, B*73:01, B*78:01, B*81:01, B*82:01, C*01:02, C*03:02, C*03:03, C*03:04, C*07:02, C*08:01, C*12:03, C*14:02, C*16:01 | B*07:14, B*15:04, B*15:06, B*15:07, B*15:18, B*15:20, B*15:21, B*15:27, B*35:02, B*35:03, B*35:08, B*35:12, B*39:02, B*39:04, B*39:05, B*39:06, B*39:13, B*40:03, B*40:03, B*40:05, B*41:02, B*42:02, B*48:02, B*50:02, B*55:02, B*55:04, B*56:03, C*01:03, C*07:01, C*07:04, C*08:02, C*08:03, C*08:04, C*12:02, C*14:03 | B62, C*16:01 | B*15:24, C*16:02. |
| **80TLR** | B*13:01, B*13:02, B*27:05, B*37:01, B*44:02, B*44:03, B*47:01 | B*27:04, B*27:06, B*38:02 | B*38:02 | B*38:01 |
| **82LR** | A*23:01, A*24:02, A*24:03, A*25:01, A*32:01, B*13:01, B*13:02, B*15:13, B*15:16, B*27:05, B*37:01, B*38:01, B*44:02, B*44:03, B*47:01, B*49:01, B*51:01, B*51:02, B*52:01, B*53:01, B*57:01, B*57:03, B*58:01, B*59:01 | B*15:17, B*15:24, B*27:04, B*27:06, B*38:02 | B*15:24 | B62 |
| **131S** | B*13:01, B*13:02, B*15:01, B*15:02, B*15:03, B*15:10, B*15:11, B*15:12, B*15:13, B*15:16, B*18:01, B*27:05, B*27:08, B*35:01, B*37:01, B*38:01, B*39:01, B*44:02, B*44:03, B*45:01, B*46:01, B*47:01, B*49:01, B*50:01, B*51:01, B*51:02, B*52:01, B*53:01, B*54:01, B*55:01, B*56:01, B*57:01, B*57:03, B*58:01, B*59:01, B*67:01, B*78:01, B*82:01 | B*15:04, B*15:06, B*15:07, B*15:17, B*15:18, B*15:20, B*15:21, B*15:24, B*15:27, B*27:04, B*27:06, B*35:02, B*35:03, B*35:08, B*35:12, B*38:02, B*39:02, B*39:04, B*39:05, B*39:06, B*39:13, B*48:02, B*50:02, B*55:02, B*56:03 | B*55:01, B*55:02 | B*55:04 |
| **138K** | C*05:01 | A*02:18, C*08:02, C*08:04 | A*02:18, C*08:02, C*08:04 | A2, C*08:01, C*08:03. |
| **177KT** | C*05:01, C*08:01 | C*07:04, C*08:02, C*08:03, C*08:04 | C*07:04 | C*07:01, C*07:02 |
| **193PV** | B*35:01, B*51:01, B*51:02, B*52:01, B*53:01, B*58:01, B*78:01, C*01:02, C*02:02, C*03:02, C*03:03, C*03:04, C*04:01, C*05:01, C*06:02, C*08:01, C*12:03, C*14:02, C*15:02, C*17:01, C*18:02 | B*15:20, B*35:02, B*35:03, B*35:08, B*35:12, B*48:02, C*01:03, C*02:10, C*04:03, C*08:02, C*08:03, C*08:04, C*12:02, C*14:03, C*15:05, C*17:03, C*18:01 | B*15:20 | B62 |

SUPPLEMENT TABLE 10. Class II Eplets Predicted to Split Antigen Groups Or Serotypes

| **Eplet** | **Standard Beads** | **Explex beads** | **Positive alleles** | **Negative alleles** |
| --- | --- | --- | --- | --- |
| **11STS** | DRB1*03:01, DRB1*03:02, DRB1*11:01, DRB1*11:04, DRB1*13:01, DRB1*13:03, DRB1*14:01, DRB1*14:02, DRB1*14:54 | DRB1*13:02, DRB1*14:03, DRB1*14:05, DRB1*14:06 | DRB1*14:01, DRB1*14:02, DRB1*14:03, DRB1*14:05, DRB1*14:06, DRB1*14:54 | DRB1*14:04 |
| **16Y** | DRB1*08:01, DRB1*12:01, DRB1*12:02 | DRB1*08:02, DRB1*08:03, DRB1*08:07, DRB1*14:04 | DRB1*14:04 | DRB1*14:01, DRB1*14:02, DRB1*14:03, DRB1*14:05, DRB1*14:06, DRB1*14:54 |
| **r37YV** | DRB1*04:01, DRB1*04:02, DRB1*04:03, DRB1*04:04, DRB1*04:05, DRB1*08:01, DRB1*11:01, DRB1*11:04, DRB1*13:03 | DRB1*04:07, DRB1*04:10, DRB1*04:11, DRB1*08:02, DRB1*08:03, DRB1*08:07 | DRB1*04:01, DRB1*04:02, DRB1*04:03, DRB1*04:04, DRB1*04:05, DRB1*04:07, DRB1*04:10, DRB1*04:11, DRB1*13:03 | DRB1*04:06, DRB1*13:01, DRB1*13:02 |
| **57S** | DRB1*04:05, DRB1*08:01, DRB1*13:03 | DRB1*04:10, DRB1*04:11, DRB1*08:03 | DRB1*04:05, DRB1*04:10, DRB1*04:11, DRB1*08:01, DRB1*08:03, DRB1*13:03 | DRB1*04:01, DRB1*04:02, DRB1*04:03, DRB1*04:04, DRB1*04:06, DRB1*04:07, DRB1*08:02, DRB1*08:07, DRB1*13:01, DRB1*13:02 |
| **r57V** | DRB1*07:01, DRB1*09:01, DRB1*12:01, DRB1*12:02, DRB3*01:01, DRB3*03:01 |  | DRB3*01:01, DRB3*03:01 | DRB3*02:01, DRB3*02:02 |
| **67QL** | DRB1*01:01, DRB1*01:02, DRB1*03:01, DRB1*03:02, DRB1*04:01, DRB1*04:03, DRB1*04:04, DRB1*04:05, DRB1*14:02, DRB3*01:01, DRB3*02:02, DRB3*03:01 | DRB1*04:06, DRB1*04:07, DRB1*04:10, DRB1*04:11, DRB1*14:06, DRB3*02:01 | DRB1*14:02, DRB1*14:06 | DRB1*14:01, DRB1*14:03, DRB1*14:04, DRB1*14:05, DRB1*14:54 |
| **70D** | DRB1*01:03, DRB1*04:02, DRB1*07:01, DRB1*08:01, DRB1*11:01, DRB1*11:04, DRB1*12:01, DRB1*12:02, DRB1*13:01, DRB1*13:03, DRB1*14:03, DRB1*16:01, DRB1*16:02, DRB5*01:01 | DRB1*08:02, DRB1*08:03, DRB1*08:07, DRB1*13:02, DRB5*01:02 | DRB1*04:02, DRB1*14:03, DRB5*01:01 DRB5*01:02 | DRB1*04:01, DRB1*04:03, DRB1*04:04, DRB1*04:05 DRB1*04:06, DRB1*04:07, DRB1*04:10, DRB1*04:11, DRB1*14:01, DRB1*14:02, DRB1*14:04, DRB1*14:05, DRB1*14:06, DRB1*14:54, DRB5*02:02 |
| **70DA** | DRB1*01:03, DRB1*04:02, DRB1*08:01, DRB1*11:01, DRB1*11:04, DRB1*12:01, DRB1*12:02, DRB1*13:01, DRB1*13:03, DRB1*16:01, DRB1*16:02, DRB5*01:01 | DRB1*08:02, DRB1*08:03, DRB1*08:07, DRB1*13:02, DRB1*14:03, DRB5*01:02 | DRB1*04:02, DRB1*14:03, DRB5*01:01, DRB5*01:02 | DRB1*04:01, DRB1*04:03, DRB1*04:04, DRB1*04:05 DRB1*04:06, DRB1*04:07, DRB1*04:10, DRB1*04:11, DRB1*14:01, DRB1*14:02, DRB1*14:04, DRB1*14:05, DRB1*14:06, DRB1*14:54, DRB5*02:02 |
| **70QT** | DRB1*01:01, DRB1*01:02, DRB1*04:01, DRB1*04:03, DRB1*04:04, DRB1*04:05, DRB1*14:02, DRB1*15:01, DRB1*15:02, DRB1*15:03, DRB5*02:02 | DRB1*04:06, DRB1*04:07, DRB1*04:10, DRB1*04:11, DRB1*14:06 | DRB1*04:01, DRB1*04:03, DRB1*04:04, DRB1*04:05 DRB1*04:06, DRB1*04:07, DRB1*04:10, DRB1*04:11,  DRB1*14:02, DRB1*14:06 | DRB1*04:02  DRB1*14:01, DRB1*14:03 DRB1*14:04, DRB1*14:05, DRB1*14:54 |
| **70R** | DRB1*09:01, DRB1*09:02, DRB1*10:01, DRB1*14:01, DRB1*14:54, DRB4*01:01, DRB4*01:03 | DRB1*14:04, DRB1*14:05 | DRB1*14:01, DRB1*14:04, DRB1*14:05, DRB1*14:54 | DRB1*14:02, DRB1*14:03 DRB1*14:06 |
| **74R** | DRB1*03:01, DRB1*03:02, DRB3*01:01 |  | DRB3*01:01 | DRB3*02:01, DRB3*02:02, DRB3*03:01 |
| **q57V** | DQB1*05:01, DQB1*06:04, DQB1*06:09 |  | DQB1*05:01, DQB1*06:04, DQB1*06:09 | DQB1*05:02, DQB1*05:03 DQB1*06:01, DQB1*06:02, DQB1*06:03 |
| **125SQ** | DQB1*05:01 | DQB1*05:03 | DQB1*05:01, DQB1*05:03 | DQB1*05:02 |
| **rp67IE** | DPB1*02:01, DPB1*09:01, DPB1*10:01, DPB1*13:01, DPB1*17:01, DPB1*19:01, DRB1*01:03, DRB1*04:02, DRB1*13:01 | DPB1*02:02, DPB1*30:01, DRB1*13:02 | DRB1*04:02, DRB1*13:01, DRB1*13:02 | DRB1*04:01, DRB1*04:03, DRB1*04:04, DRB1*04:05, DRB1*04:06, DRB1*04:07, DRB1*04:10, DRB1*04:11, DRB1*13:03 |
| **rq70RK/R** | DQB1*02:01, DQB1*02:02, DRB1*09:01, DRB1*09:02, DRB1*10:01, DRB1*14:01, DRB1*14:54, DRB4*01:01, DRB4*01:03 | DRB1*14:04, DRB1*14:05 | DRB1*14:01, DRB1*14:04, DRB1*14:05, DRB1*14:54 | DRB1*14:02, DRB1*14:03, DRB1*14:06 |

SUPPLEMENTARY TABLE 11. DP Eplets predicted to react with DP beads on the Explex panel. *DPB1*105:01 and DPB1*04:02 are in the same P-group.

| **Eplet** | **Standard Beads** | **Explex beads** | **Corresponding**  **Negative Eplet** |
| --- | --- | --- | --- |
| **35FV** | DPB1*02:01, DPB1*03:01, DPB1*04:02, DPB1*06:01, DPB1*09:01, DPB1*10:01, DPB1*14:01, DPB1*17:01, DPB1*18:01, DPB1*19:01, DPB1*20:01, DPB1*23:01 | DPB1*30:01, DPB1*105:01* | 35YA eplet |
| **56A** | DPB1*01:01, DPB1*04:01, DPB1*05:01, DPB1*11:01, DPB1*13:01, DPB1*15:01, DPB1*19:01, DPB1*23:01 | DPB1*02:02, DPB1*26:01, DPB1*30:01, DPB1*31:01, DPB1*40:01, DPB1*85:01, DPB1*107:01 | 56E eplet |
| **56E** | DPB1*02:01, DPB1*03:01, DPB1*04:02, DPB1*06:01, DPB1*09:01, DPB1*10:01, DPB1*14:01, DPB1*17:01, DPB1*18:01, DPB1*20:01, DPB1*28:01 | DPB1*105:01* | 56A eplet |
| **56EE** | DPB1*02:01, DPB1*04:02, DPB1*10:01, DPB1*18:01, DPB1*28:01 | DPB1*105:01* | 56A eplet + DPB1*03:01, DPB1*06:01, DPB1*09:01, DPB1*14:01, DPB1*17:01, DPB1*20:01 |
| **69E** | DPB1*02:01, DPB1*06:01, DPB1*09:01, DPB1*10:01, DPB1*13:01, DPB1*18:01, DPB1*19:01 | DPB1*02:02, DPB1*30:01 | 69K and 69R eplets |
| **84DEAV** | DPB1*01:01, DPB1*03:01, DPB1*05:01, DPB1*06:01, DPB1*09:01, DPB1*10:01, DPB1*11:01, DPB1*13:01, DPB1*14:01, DPB1*17:01, DPB1*19:01, DPB1*20:01 | DPB1*26:01, DPB1*30:01, DPB1*31:01, DPB1*85:01, DPB1*107:01 | 85GPM eplet |
| **85GPM** | DPB1*02:01, DPB1*04:01, DPB1*04:02, DPB1*15:01, DPB1*18:01, DPB1*23:01, DPB1*28:01 | DPB1*02:02, DPB1*40:01, DPB1*105:01* | 84DEAV eplet |
| **96K** | DPB1*01:01, DPB1*03:01, DPB1*05:01, DPB1*06:01, DPB1*09:01, DPB1*10:01, DPB1*11:01, DPB1*13:01, DPB1*14:01, DPB1*15:01, DPB1*18:01, DPB1*19:01, DPB1*20:01 | DPB1*26:01, DPB1*85:01, DPB1*107:01 | 96R eplet |
